# Synthesis and Development of 3-((2,4-Difluorophenyl)Amino)Propanoic Acid Derivatives as an Antiproliferative Medicinal Chemistry Scaffold Targeting Growth Factor Receptors

**DOI:** 10.3390/ph19030381

**Published:** 2026-02-27

**Authors:** Guoda Pranaitytė, Povilas Kavaliauskas, Vidmantas Petraitis, Rūta Petraitienė, Ramunė Grigalevičiūtė, Liudas Ivanauskas, Mindaugas Marksa, Gediminas Duda, Waldo Acevedo, Birutė Grybaitė, Vytautas Mickevičius

**Affiliations:** 1 Department of Organic Chemistry, Kaunas University of Technology, Radvilenu˛ Rd. 19, LT-50254 Kaunas, Lithuania; 2 Department of Microbiology and Immunology, University of Maryland School of Medicine, Baltimore, MD 21201, USA; 3 Biological Research Center, Lithuanian University of Health Sciences, LT-44307 Kaunas, Lithuania; 4 Center for Discovery and Innovation, Hackensack Meridian Health, Nutley, NJ 07110, USA; vidmantas.petraitis@hmh-cdi.org (V.P.); ruta.petraitiene@hmh-cdi.org (R.P.); 5 Department of Animal Nutrition, Lithuanian University of Health Sciences, LT-44307 Kaunas, Lithuania; 6 Department of Analytical and Toxicological Chemistry, Lithuanian University of Health Sciences, LT-50161 Kaunas, Lithuania; liudas.ivanauskas@lsmu.lt (L.I.);; 7 Instituto de Química, Facultad de Ciencias, Pontificia Universidad Católica de Valparaíso, Valparaíso 2373223, Chile; waldo.acevedo@pucv.cl; 8 Center for Interdisciplinary Research in Biomedicine, Biotechnology and Well-Being (CID3B), Pontificia Universidad Católica de Valparaíso, Valparaíso 2340025, Chile

**Keywords:** azoles, *β*-amino acid, hydrazones, 2,4-difluorophenyl moiety, antiproliferative activity

## Abstract

**Background/Objectives:** The development of novel small-molecule kinase inhibitors remains an important strategy in anticancer drug discovery. Receptor tyrosine kinases such as c-MET and HER2 are clinically relevant targets involved in tumor progression and resistance mechanisms. The aim of this study was to design, synthesize, and biologically evaluate a series of 3-[(2,4-difluorophenyl)amino]propanoic acid derivatives as potential antiproliferative agents and to explore their possible interactions with selected kinase targets. **Methods:** A series of ester, hydrazide, hydrazone, semicarbazide, triazolone, and triazolethione derivatives (2–21) were synthesized and structurally characterized by NMR, IR spectroscopy, and microanalysis. The compounds were evaluated for in vitro anticancer activity against A549 and Caco-2 human cancer cell lines. In addition, molecular docking studies were performed to investigate binding interactions with c-MET and HER2 receptor tyrosine kinases. Cytotoxicity toward non-transformed HEK293 cells was also assessed. **Results:** The synthesized derivatives demonstrated structure–activity relationships, with compounds **6b**, **7f**, **7g**, and **9** exhibiting the most pronounced antiproliferative effects, reducing cancer cell viability by approximately 50% in both tested cell lines. Molecular docking indicated that compound **9** displayed favorable predicted binding energies toward c-MET and HER2, forming hydrophobic and hydrogen-bond interactions within the active sites and showing overlapping contacts with native ligands and reference inhibitors. Active compounds also demonstrated cytotoxic effects in HEK293 cells comparable to those of doxorubicin and cisplatin. **Conclusions:** These results identify 3-[(2,4-difluorophenyl)amino]propanoic acid derivatives, particularly compound **9**, as promising scaffolds for further structural optimization toward the development of kinase-targeting antiproliferative agents.

## 1. Introduction

Cancer remains one of the leading causes of morbidity and mortality worldwide, accounting for an estimated 20 million newly diagnosed cases in 2022. Global epidemiological projections indicate that this burden will continue to rise, with annual cancer incidence expected to exceed approximately 33 million new cases by 2050 [1,2]. Among men, prostate, lung and colorectal cancer account for 48% of all diagnosed cases, while women are most commonly affected by breast, lung and colon cancer [3]. With the increasing number of oncological diseases worldwide, the demand for chemotherapeutic drugs is constantly growing, so the development of new bioactive compounds for the treatment of cancer remains relevant [4,5].

Conventional chemotherapeutic drugs generally exhibit low selectivity, targeting rapidly dividing cells rather than tumor-specific molecular pathways. Consequently, healthy proliferating tissues are damaged, leading to serious systemic adverse effects and treatment-limiting toxicities [6,7,8,9]. Moreover, the acquisition of genetic mutations can render cancer cells resistant to existing chemotherapeutic agents [10,11,12,13]. Despite continuous advances in anticancer drug discovery, the therapeutic benefit of many new agents is hindered by mutational diversity, disseminated disease, and adaptive resistance mechanisms. Consequently, there is a compelling need to develop innovative molecular scaffolds that can overcome these barriers and provide more effective strategies for cancer treatment.

**Figure 1:**
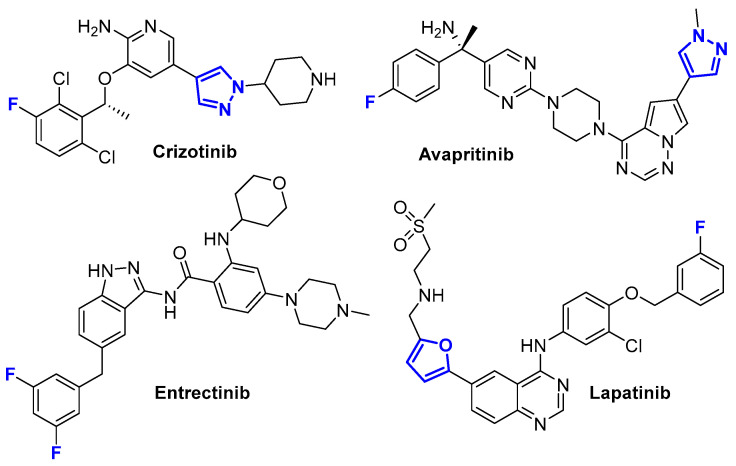
Anticancer molecules, containing aromatic fluorine or heterocyclic moiety [19,21].

Growth factor receptors are key molecular drivers of tumorigenesis and represent critical therapeutic targets in modern cancer therapy. Among the most clinically validated receptor families are the human epidermal growth factor receptor (HER/ErbB) axis, including EGFR and HER2, and the hepatocyte growth factor receptor c-MET [14,15]. Aberrant activation of these receptors through overexpression, mutational gain-of-function, gene amplification, or autocrine ligand stimulation leads to enhanced proliferative signaling, enhanced invasion, resistance to apoptosis, and metastatic progression [16]. Current chemotherapeutic and targeted agents exploit these crucial targets involved in neoplasm processes. EGFR inhibitors such as erlotinib, gefitinib, and osimertinib are widely used in non-small-cell lung cancer, while; HER2-targeted monoclonal antibodies and tyrosine kinase inhibitors—including trastuzumab, pertuzumab, and lapatinib—have transformed the treatment of HER2-positive breast cancer (Figure 1). Finally, and c-MET inhibitors like crizotinib and capmatinib block HGF/MET-driven oncogenic signaling. Mechanistically, these drugs suppress downstream oncogenic pathways such as RAS–RAF–MEK–ERK and PI3K–AKT–mTOR, ultimately inhibiting proliferation and promoting apoptosis. Targeting growth factor receptor pathways is therefore crucial, as these signaling nodes regulate essential hallmarks of cancer and contribute to therapeutic resistance, making them an important focus for the development of next-generation anticancer scaffolds and combination strategies [17,18,19,20].

Heterocyclic fragments are fundamental building blocks in modern drug design, owing to their ability to modulate physicochemical properties, enhance receptor binding, and improve pharmacokinetic profiles [21,22]. Heterocyclic structures have become central to modern anticancer drug discovery, as their diverse electronic and steric characteristics enable selective interactions with cancer-associated enzymes, receptors, and nucleic acid targets. Their natural prevalence in biological macromolecules—including nucleic acids, enzymes, and vitamin cofactors—further supports their capacity to disrupt oncogenic signaling networks and metabolic processes [23,24].

In recent decades, aromatic fluorine-containing compounds have gained increasing importance in the development of new therapeutic molecules [25,26]. Fluorine-containing heterocycles are often highly attractive structural motifs in targeted anticancer drug design, particularly against growth factor receptor–driven malignancies. The incorporation of fluorine atoms into heterocyclic scaffolds can profoundly influence molecular behavior by increasing lipophilicity and improving compatibility with hydrophobic pockets present within receptor tyrosine kinases. These interactions not only enhance binding affinity, but also contribute to improved conformational stability and optimal positioning of the active compound within the catalytic domain, thereby strengthening inhibitory potency [27,28,29]. Furthermore, fluorination can increase metabolic stability by blocking oxidative degradation, prolonging compound half-life, and improving pharmacokinetic performance. In addition, the introduction of fluorine atoms can fine-tune electronic distribution within the heterocycle, enabling more precise alignment with key amino acid residues involved in receptor signaling [30].

Building upon our previous study on 1-(2,4-difluorophenyl)-5-oxopyrrolidine-3-carboxylic acid derivatives, which demonstrated promising antiproliferative activity against triple-negative breast, prostate cancer, and melanoma cell lines in both 2D and 3D models [31], the 2,4-difluorophenyl fragment was selected for further structural exploration. Based on these findings, a new series of compounds incorporating the 2,4-difluorophenyl moiety, β-alanine backbones, hydrazone linkages, and heterocyclic substituents was designed and synthesized as potential antiproliferative agents. Their anticancer potential was initially assessed in vitro using A549 lung carcinoma and Caco-2 colorectal adenocarcinoma epithelial cell models, followed by in silico molecular modeling to further characterize receptor–ligand interactions and predict binding positions.

## 2. Results

### 2.1. Synthesis and Characterization of 3-[(2,4-Difluorophenyl)Amino]Propanoic Acid Derivatives

**Scheme 1:**
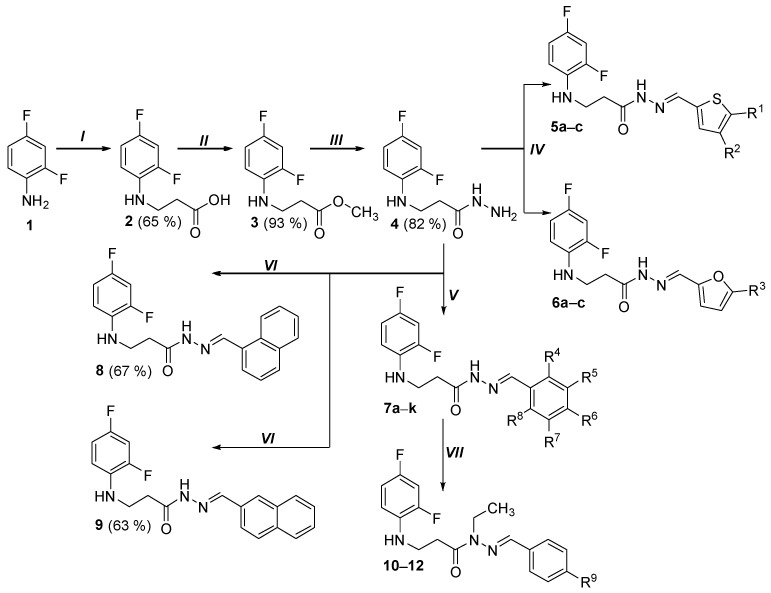
Synthesis of compounds **2**–**12**. **5a** (91%) R^1^, R^2^ = H; **5b** (91%) R^1^ = NO_2_, R^2^ = H; **5c** (90%) R^1^ = H, R^2^ = Br; **6a** (81%) R^3^ = H; **6b** (89%) R^3^ = NO_2_; **6c** (83%) R^3^ = Br; **7a** (89%) R^4^, R^5^, R^6^, R^7^, R^8^ = H; **7b** (93%) R^4^, R^5^, R^7^, R^5^ = H, R^6^ = F; **7c** (78%) R^5^, R^7^, R^8^ = H, R^4^, R^6^ = F; **7d** (86%) R^4^, R^5^, R^7^, R^8^ = H, R^6^ = Cl; **7e** (86%) R^4^, R^5^, R^7^, R^8^ = H, R^6^ = Br; **7f** (91%) R^4^, R^5^, R^7^, R^8^ = H, R^6^ = CH_3_; **7g** (87%) R^4^, R^5^, R^7^, R^8^ = H, R^6^ = OCH_3_; **7h** (75%) R^4^, R^8^ = H, R^5^, R^6^, R^7^ = OCH_3_; **7i** (92%) R^4^, R^5^, R^7^, R^8^ = H, R^6^ = N(CH_3_)_2_; **7j** (92%) R^4^, R^5^, R^7^, R^8^ = H, R^6^ = NO_2_; **7k** (87%) R^4^ = Cl; R^5^, R^6^, R^8^ = H, R^7^ = NO_2_; **10** (47%) R^9^ = H; **11** (46%) R^9^ = Cl; **12** (47%) R^9^ = CH_3_. Reagents and conditions: (***I***) acrylic acid, toluene, Δ, 24 h; (***II***) MeOH, H_2_SO_4_, Δ, 5h; (***III***) N_2_H_4_∙H_2_O, Δ, 20 h, *i*-PrOH; (***IV***) corresponding heterocyclic aldehyde, *i*-PrOH, Δ, 1 h; (***V***) corresponding aromatic aldehyde, *i*-PrOH, Δ, 1 h; (***VI***) corresponding naphthaldehyde, *i*-PrOH, Δ, 2 h; (***VII***) CH_3_CH_2_I, KOH, K_2_CO_3_, DMF, RT, 1 h.

An analysis of scientific literature has revealed that *β*-amino acids [32,33,34,35,36,37,38], hydrazides and hydrazones [39,40] are widely used in the synthesis of biologically active compounds, and fluorine atoms in an aromatic ring often affect the selectivity and activity of medicinal compounds [39,40]. Aromatic fluorine fragment is also found in the structures of medicinal molecules intended for the treatment of cancer [21,28]. The reaction of 2,4-difluoroaniline (**1**) with acrylic acid produced the starting substrate 3-[(2,4-difluorophenyl)amino]propanoic acid (**2**), in order to synthesize molecules with aromatic fluorine moiety and *β*-amino acid fragment. Hydrazides play an important role as intermediate organic compounds in the creation of potential therapeutic molecules [39]. Since hydrazides are more easily obtained from esters than from acids, 3-[(2,4-difluorophenyl)amino]propanoic acid (**2**) was treated with methanol in the presence of a catalytic amount of sulfuric acid in the reaction mixture, resulting in methyl 3-[(2,4-difluorophenyl)amino]propanoate (**3**). Dissolving esters in 2-propanol and adding hydrazine monohydrate excess to the reaction mixture yielded 3-[(2,4-difluorophenyl)amino]propanehydrazide (**4**) (Scheme 1). When analyzing the ^1^H NMR spectra of aminopropanoic acid **2**, a broad singlet characteristic of the -COOH group proton can be observed at 12.26 ppm. The presence of this group is also confirmed by the peak at 173.28 ppm in ^13^C NMR spectra, which is characteristic of the carbonyl carbon. In the aromatic part of ^13^C NMR spectra, due to two fluorine atoms, a splitting of the carbon atom signals in the interval 103.01–154.4 ppm is visible. It is important to note that due to the interaction of carbon atoms with fluorine nuclei, the signals of the carbon atoms of the aromatic ring can be split to provide four peaks instead of single peak [41].

It has been found that hydrazone moieties attached to heterocyclic systems are characterized by increased biological activity of target compounds [42], so in order to investigate which structural fragments will determine the best biological activity, a series of hydrazide **4** condensation reactions with heterocyclic, aromatic aldehydes were performed. The products **5a**–**c**, **6a**–**c**, **7a**–**k**, **8**, **9** of these reactions were obtained by dissolving the starting compound **4** in 2-propanol, then adding the corresponding aldehyde and stirring the reaction in 1 to 2 h at the boiling temperature of the mixture (Scheme 1).

**Scheme 2:**
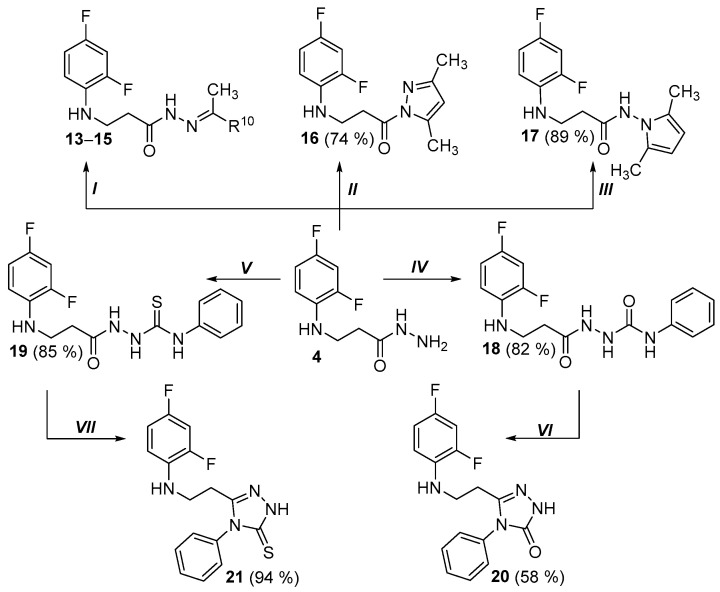
Synthesis of compounds **13**–**21**. **13** (72%) R^10^ = CH_3_; **14** (70%) R^10^ = C_2_H_5_; **15** (73%) R^10^ = 4-H_2_N-C_6_H_4_. Reagents and conditions: (***I***) acetone (**13**), ethyl methyl ketone (**14**) or 4-aminoacetophenone (**15**), *i*-PrOH (**15**), Δ, 3 h; (***II***) pentane-2,4-dione, *i*-PrOH, HCl, Δ, 2h; (***III***) hexane-2,5-dione, *i*-PrOH, CH_3_COOH, Δ, 2 h; (***IV***) PhNCO, MeOH, Δ, 5 h; (***V***) PhNCS, MeOH, Δ, 1h; (***VI***) 4% NaOH, Δ, CH_3_COOH pH 6, 2 h; (***VII***) 4% NaOH, Δ, CH_3_COOH pH 6, 1.5 h.

In the case of hydrazones **13** and **14**, acetone and ethyl methyl ketone in these reactions was both solvent and reagent, compound **15** was obtained carrying out the reaction in 2-propanol in the presence of 4-aminoacetophenone (Scheme 2). Hydrazones **5a**–**c**, **6a**–**c**, **7a**–**k**, **8**, **9** and **14**, **15** in DMSO-*d*_6_ solution exist as a mixture of *E/Z* isomers as indicated by the proton signals of azomethine fragment and the amide -NH group in ^1^H NMR spectra, each of which is visible as a set of two singlets with a signal intensity ratio of 60:40. This pattern in the spectra is characteristic of hydrazone compounds due to the presence of -CO-NH- fragment in the molecules and the restricted rotation around this bond. Based on the results of X-ray structural analysis of similar compounds performed earlier, it can be stated that of the possible geometric isomers, only the *E* isomer is formed, so it also predominates in hydrazone solutions [43]. In order to evaluate influence of *N*-substitution to biological activity of hydrazones, alkylation of several (*E*)-*N*’-benzylidene-3-[(2,4-difluorophenyl)amino]propanohydrazides were investigated. For this purpose, starting material (**7a**, **7d** or **7f**) was dissolved in DMF, treated with excess of iodoethane at the room temperature in the presence of potassium hydroxide and potassium carbonate in the reaction mixture (Scheme 1). Structures of the target alkylated hydrazones **10**–**12** were proven by visible two additional peaks in the ^13^C NMR spectra belonging to the carbon atoms of the methylene (-CH_2_-) and methyl (-CH_3_) groups in the intervals 10.94–11.04 ppm and 39.37–39.43 ppm Comparing the FT-IR spectroscopy data for hydrazones **7a**, **7d**, **7f** and their alkylated analogues **10**–**12**, it is seen that in the *N*-substituted compounds, the absorption bands characteristic of the amide fragment -NH group is no longer visible.

Fragments of nitrogen heterocyclic compounds are often found in the structures of medicinal molecules [22], for example, pyrrole moiety can be recognized in molecules with anticancer properties [44]. Some of the drug molecules with not only anticancer but also anti-inflammatory activity contain a pyrazole fragment [45]. The reaction with hydrazones and diketones produced compounds with five-membered heterocyclic fragments containing one or two nitrogen atoms in the cycle was synthesized, yielding 3-[(2,4-difluorophenyl)amino]-1-(3,5-dimethyl-1*H*-pyrazol-1-yl)propan-1-one (**16**) and 3-[(2,4-difluorophenyl)amino]-*N*-(2,5-dimethyl-1*H*-pyrrol-1-yl)propanamide (**17**). In this case, an acidic catalyst is required for the formation of heterocycles, so catalytic amounts of hydrochloric and acetic acid, respectively, were used to obtain compounds **16** and **17**. These reactions were performed based on conditions described in our previously published studies [36,43,46]. Protons of methyl group, present in compound **16**, were observed as two singlets at 2.21 and 2.54 ppm, while in compound **17** high intensity singlet is seen at 1.95 ppm. Pyrazole ring proton is observed in the spectra as singlet at 6.21 ppm, while the protons of pyrrole ring (compound **17**) are observed as two singlets: 5.62 ppm and 5.70 ppm.

Compounds with (thio)semicarbazide fragments are promising potential therapeutic agents due to the abundance of hydrogen bond donor groups they contain. Replacement of sulfur with an oxygen atom in thiosemicarbazide derivatives increases antitumor activity, but also toxicity; therefore, it is important to develop new (thio)semicarbazide derivatives to optimize biological activity and reduce toxicity [47]. In order to synthesize carbo(thio)amides, the reactions of propanehydrazide **4** and phenylisocyanate or phenylisothiocyanate in methanol were performed. The correspondingly synthesized 2-{3-[(2,4-difluorophenyl)amino]propanoyl}-*N*-phenylhydrazine-1-carboxamide (**18**) and 2-{3-[(2,4-difluorophenyl)amino]propanoyl}-*N*-phenylhydrazine-1-carbothioamide (**19**) were transformed into triazolone (compound **20**) and triazolethione (compound **21**) ring-containing molecules via an intramolecular condensation reactions by treating the compounds **18** and **19** with 4% sodium hydroxide solution at the boiling temperature of the mixture. The increase in peaks visible in the aromatic part of ^13^C NMR spectra proves that an additional phenyl ring has appeared in semicabazides (**18**, **19**). Analysis of the FT-IR spectra shows that two amino groups remain in structures **20** and **21**, while in compound **21** there is no longer an absorption band indicating a C=O group; in the case of product **20**, only one intense absorption band is observed at 1689 cm^−1^. The structures of the obtained compounds and their yields (%) are shown in Table 1.

### 2.2. Antiproliferative Activity of 3-[(2,4-Difluorophenyl)Amino]Propanoic Acid Derivatives Against A549 and Caco-2 Cell Lines

After synthesizing and fully characterizing the 3-[(2,4-difluorophenyl)amino]propanoic acid derivatives **2–21**, we proceeded to assess their in vitro antiproliferative activity in human cancer cell models. The A549 human lung adenocarcinoma cell line and the Caco-2 human colorectal adenocarcinoma cell line were selected as well-established and widely used in vitro models for the preliminary evaluation of anticancer activity.

**Figure 2:**
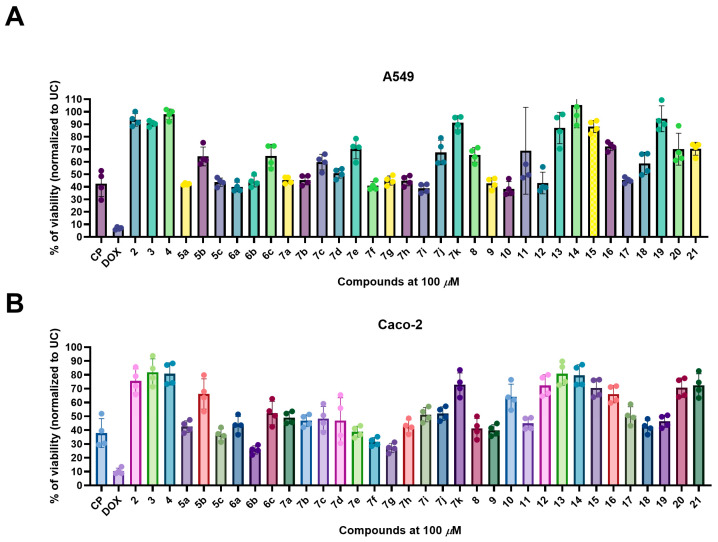
Antiproliferative activity of 3-[(2,4-difluorophenyl)amino]propanoic acid derivatives (**2**–**21**) against A549 (**A**) and Caco-2 (**B**) cell lines. Cells were treated with each compound at a fixed concentration of 100 µM, while doxorubicin (DOX) and cisplatin (CP) served as cytotoxicity controls. Cells exposed to media only with 0.5% DMSO were referred to untreated control (UC). Cell viability was assessed by the MTT assay. Data are presented as the mean ± SD from three independent experimental replicates.

Compound-mediated antiproliferative activity was quantified using the MTT colorimetric assay, with all test compounds evaluated at a fixed concentration of 100 µM. Cell viability following treatment was compared to two clinically used reference chemotherapeutics—doxorubicin (DOX) and cisplatin (CP)—both of which are FDA-approved agents employed in the management of lung and colorectal malignancies (Figure 2).

The parent scaffold and early synthetic intermediates (compounds **2**–**4**), which lack aromatic or heterocyclic extension through hydrazone formation, exhibited only minimal antiproliferative activity, with viability exceeding 80% in both models. Hydrazones bearing a heterocyclic moiety (series **5a**–**c** and **6a**–**c**) demonstrated a strong dependence on the electronic nature of the substituents. In both A549 and Caco-2 cells, compounds **5b** and **6b**, bearing the 5-nitro-2-thienyl and 5-nitro-2-furyl fragments, showed antiproliferative activity in A549 and Caco-2 models. In contrast, heterocyclic hydrazones bearing a bromine atom in the ring (**5c** and **6c**) exhibited antiproliferative activity, whereas the unsubstituted hydrazones (**5a** and **6a**) were showed similar activity.

Within the benzylidene hydrazone series (**7a**–**k**), substitution pattern influenced antiproliferative effects. The nitro-substituted derivative **7k** exhibited moderate activity in Caco-2 cells (45% viability), whereas **7j** demonstrated weaker effects in both models. Overall, activity within this series varied depending on both electronic substitution and cell line, without a uniformly dominant derivative across both models. Halogenated analogues (**7d** and **7e**) showed antiproliferative activity, while hydrazones containing electron-donating substituents such as methoxy (**7g**, **7h**), methyl (**7f**), or dimethylamino (**7i**) groups were weak or inactive, maintaining viability above 70% (Figure 2A,B).

*N*-alkylated hydrazones (compounds **10**–**12**), generated through modification of the terminal hydrazide nitrogen, showed reduced antiproliferative activity relative to their non-alkylated precursors (compounds **7a**, **7d**, **7f**). These compounds generally produced moderate activity (50–60% viability), suggesting that the free hydrazide NH group contributes to antiproliferative activity, likely through hydrogen bonding or maintenance of conformational preferences conducive to target binding. Removal of this NH functionality therefore attenuates activity, making NH functional group important for antiproliferative activity.

Incorporation of heterocyclic fragments resulted in a distinct structure–activity relationship (SAR), characterized by moderate and less substituent-sensitive antiproliferative effects compared with the pronounced electronic substituent dependence observed in the hydrazone series. 3-[(2,4-Difluorophenyl)amino]-1-(3,5-dimethyl-1*H*-pyrazol-1-yl)propan-1-one **(16)** and 3-[(2,4-difluorophenyl)amino]-*N*-(2,5-dimethyl-1*H*-pyrrol-1-yl)propanamide **(17)** exhibited moderate cytotoxicity, with slightly stronger effects observed in Caco-2 cells. These data imply that five-membered aromatic heterocycles enhance molecular planarity and lipophilicity, and introduce heteroatoms capable of engaging in π-stacking or hydrogen bonding interactions, although these features did not achieve the potency observed with nitro-substituted hydrazones **5b** and **6b**. Likewise, the semicarbazide **18** and the thiosemicarbazide **19** and the cyclized triazolone/triazolethione analogues **20** and **21** showed only modest activity. Although these scaffolds introduce additional aromatic systems and hydrogen-bond donor groups, they lack the strong electron-withdrawing characteristics that appear to be central to hydrazone-mediated cytotoxicity (Figure 2).

**Figure 3:**
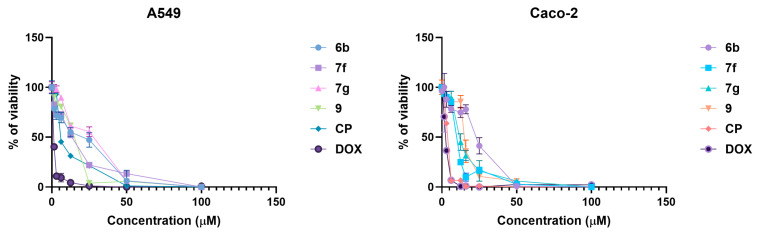
Dose–response curves and IC_50_ determination for selected 3-[(2,4-difluorophenyl)amino]propanoic acid derivatives **6b**, **7f**, **7g**, and **9** in A549 and Caco-2 cell lines. Cells were treated with increasing concentrations of each compound, while doxorubicin (DOX) and cisplatin (CP) were included as reference chemotherapeutic agents. Cell viability was assessed using the MTT assay. Data are presented as the mean ± SD from three independent experiments.

Based on the initial single-concentration screening, compounds **6b**, **7f**, **7g**, and **9** were selected for further pharmacological characterization due to their comparatively strong antiproliferative activity within their respective structural classes. These derivatives were subjected to dose–response evaluation in A549 and Caco-2 cells to determine their half-maximal inhibitory concentrations (IC_50_), with cisplatin (CP) and doxorubicin (DOX) included as reference compounds (Figure 3, Table S1).

Dose–response analysis showed that the nitro-heteroaryl hydrazone **6b**, bearing a strongly electron-withdrawing 5-nitro-2-thienyl substituent, exhibited moderate antiproliferative potency with IC_50_ values of 18.62 µM in A549 and 17.84 µM in Caco-2 cells. Among the benzylidene hydrazones, **7f**, containing an electron-donating methyl group, displayed improved activity relative to unsubstituted analogues (IC_50_ = 13.47–13.06 µM), whereas the methoxy-substituted derivative **7g** was less potent (IC_50_ = 21.18–18.92 µM), indicating a detrimental effect of increased polarity. Compound **9**, representing a distinct scaffold lacking a hydrazone linkage, showed activity with IC_50_ = 12.86 µM in A549 and 15.41 µM in Caco-2 respectively (Figure 3, Table S1).

**Figure 4:**
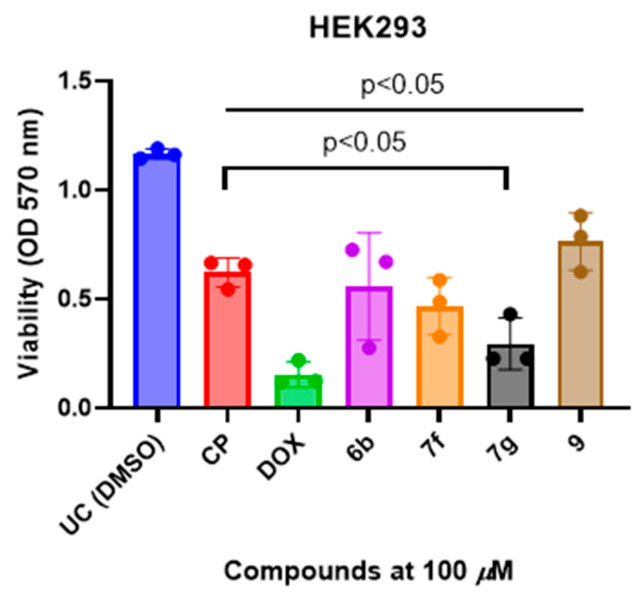
Cytotoxicity assessment of selected 3-[(2,4-difluorophenyl)amino]propanoic acid (**6b**, **7f**, **7g**, and **9**) in non-malignant HEK293 cells. Cells were treated with each compound at a fixed concentration of 100 µM. Doxorubicin (DOX) and cisplatin (CP) were included as reference cytotoxic agents, and DMSO served as the vehicle control. Cell viability was determined using the MTT assay and is expressed relative to untreated controls. Data are presented as the mean ± SD from three independent experiments.

To assess the selectivity of the most relevant anticancer hits, we evaluated compounds **6b**, **7f**, **7g**, and **9** in non-malignant HEK293 human kidney cells (Figure 4). Based on the single-concentration screening, compounds **6b**, **7f**, **7g**, and **9** were selected for further evaluation. Selection was guided not solely by maximal growth inhibition at 100 µM, but by a combination of reproducible antiproliferative effects, structural diversity across the series, and manageable cytotoxicity in non-malignant cells.

Notably, the hydrazone series demonstrated a more pronounced dependence on electronic substituents, suggesting that electronic modulation of the aromatic moiety may influence target interactions. In contrast, the heterocyclic derivatives displayed more moderate and less substituent-sensitive activity, indicating that the heterocyclic core itself may play a dominant role in determining antiproliferative effects within this subset. Although the compound set is limited, these observations suggest that both electronic factors and scaffold variation contribute to biological activity.

Assessment of compound effects in HEK293 cells at a single concentration was performed as an initial screen for non-selective cytotoxicity. These data provide qualitative insight into cellular tolerance but do not allow quantitative determination of selectivity. HEK293 cells were chosen as a non-cancerous control model because they originate from normal human tissue and lack oncogenic transformation, therefore serving as a reliable reference to evaluate compound toxicity toward healthy cells. While antiproliferative activity was assessed in A549 and Caco-2 cancer cells, Figure 4 was designed to examine the behavior of selected compounds in non-malignant HEK293 cells as an indicator of relative toxicity The nitro-substituted hydrazone **6b**, which was among the most potent compounds in A549 and Caco-2 cells, also showed measurable toxicity in HEK293, consistent with the high electrophilicity and reactivity conferred by the nitro group. The methyl-substituted analogue **7f** demonstrated cytotoxicity, mirroring its anticancer activity and reflecting the weaker electron-donating character of the CH_3_ group. In contrast, the methoxy-substituted compound **7g**, although weakly active in tumor cells, exhibited comparatively greater toxicity in HEK293. Finally, compound **9**, which lacks strongly electron-modulating substituents, displayed minimal toxicity in HEK293 (Figure 4).

### 2.3. In Silico Modeling and Target Prediction

We conducted in silico molecular docking studies to identify potential biological targets for the antiproliferative compounds (**6b**, **7f**, **7g**, and **9**) to gain insights into their possible mechanisms of action. To achieve this, we predicted the potential docking sites of these compounds within several cancer-related proteins and calculated their corresponding binding energies (∆G_bin_). For reliable results, we narrowed the search space to a selection of cancer-related proteins with known 3D structures, performing independent searches with the set of compounds and utilizing their most stable conformers in interactions with biological targets. The average binding energy for each compound was calculated as the arithmetic mean of the predicted Δ_G__bind values obtained from docking against all screened protein targets listed in Table 2. Table 2 shows the virtual screening results, which indicates that most antiproliferative compounds bind stronger to mesenchymal-epithelial transition factor (c-MET) with values ranging from −11.1 to −9.1 (average −9.65 kcal/mol) and epidermal growth factor receptor 2 (HER2), with ∆G_bin_ values ranging from −10.7 to −9.3 (average −9.76) kcal/mol.

**Figure 5:**
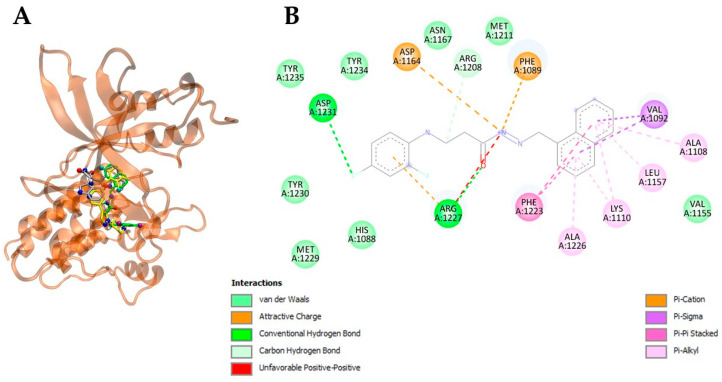
Compound **9** is predicted to bind within the hepatocyte growth factor receptor (c-MET) active site. Panel (**A**) displays the docking visualization, highlighting the predicted positioning of compound **9** (green), the co-crystallized ligand (silver), and crizotinib (yellow) within the c-MET binding pocket. Panel (**B**) shows the 2D interaction map illustrating the key hydrogen-bonding and hydrophobic contacts between compound **9** and c-MET residues. Hydrophobic interactions include van der Waals forces, π–π stacking, and π-alkyl contacts.

**Figure 6:**
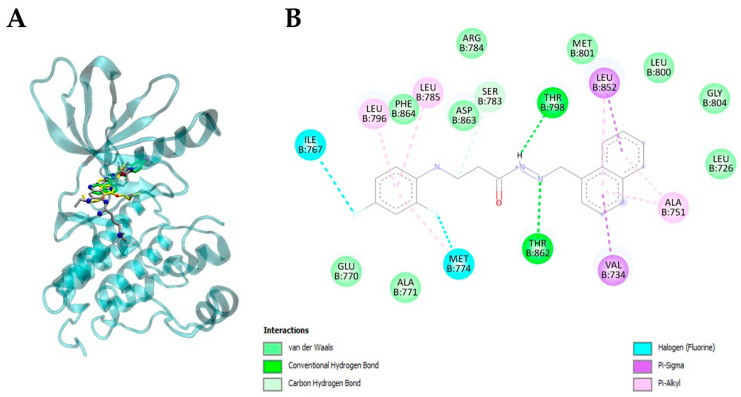
Compound **9** is predicted to bind within the HER2 active site. Panel (**A**) shows the visualization of the predicted binding positions of compound **9** (green), the co-crystallized ligand (silver), and erlotinib (yellow) within the HER2 binding pocket. Panel (**B**) presents the 2D interaction map demonstrating the hydrophobic contacts formed between compound **9** and HER2 residues. Hydrophobic interactions include van der Waals forces, π–sigma, π–π stacked, and π–alkyl interactions.

Interestingly, compound **9** consistently demonstrated the most favorable binding behavior across the tested protein panel, displaying the lowest mean binding energy value (−9.40 kcal/mol), which is fully aligned with its superior interaction energies toward c-MET (−9.65 kcal/mol) and HER2 (−9.76 kcal/mol) compared with the other tested *β*-alanine hybrids. These values were not only markedly lower than the global average interaction energies for c-MET (−8.35 kcal/mol) and HER2 (−8.09 kcal/mol) across the compound library, but also surpassed closely related structural analogues **6b**, **7f**, and **7g** by a margin of 0.8–1.5 kcal/mol. Such energetic advantages support the hypothesis that compound **9** possesses a preferential affinity toward growth-factor-associated receptor kinases, particularly those implicated in epithelial tumorigenesis (Table 2, Figure 5 and Figure 6).

When compared with crizotinib and erlotinib, the energetic aspects of the interaction remained favorable for compound **9**, presenting an improvement of 2.1 and 2.6 kcal/mol for c-MET and HER2, respectively. These results suggest that compound **9** may compete efficiently within the ATP-binding clefts of both kinases, potentially enabling a dual inhibition profile. Conversely, when **9** was compared directly with the crystallographic ligands bound to c-MET and HER2, its interaction values were found to be −3.5 and −3.9 kcal/mol less favorable, reflecting the expected high-affinity optimization of native ligands within their own structural environments. Nonetheless, this energetic penalty does not diminish the significance of the binding improvement relative to erlotinib, which remains a clinically used tyrosine kinase inhibitor.

Importantly, the naphthalene substituent present in **9** appears to play an important role in strengthening these interactions. Structural visualization and alignment studies revealed that this bulky, hydrophobic aromatic system inserts deeply into the catalytic binding grooves of c-MET and HER2 (Figure 5 and Figure 6), improving shape complementarity and enhancing π–π stacking with aromatic amino acids located near the hinge region. Notably, naphthalene contributes to an improved overlap with the co-crystallized ligands and enhances van der Waals stabilization at the core of the receptor pocket, providing a mechanistic explanation for the affinity improvement relative to simpler phenyl- or heteroaromatic-substituted analogues.

Compound **9** established a rich network of stabilizing interactions within both kinase targets, particularly c-MET and HER2, supporting its strong predicted affinity profiles. In c-MET, compound **9** engaged in extensive hydrophobic contacts with key residues located in the catalytic pocket, including Phe1089, Val1092, Ala1108, Met1211, Phe1223, Ala1226, and Arg1227, while also forming carbon-hydrogen and hydrogen-bonding interactions with residues such as Asp1164, Asn1167, Arg1208, Asp1231, Tyr1234, and Tyr1235 (Figure 5, Table 3).

Several of these interactions overlapped with those observed for the crystallographic ligand and erlotinib, including Phe1089, Val1092, Ala1108, Lys1110, Leu1157, Asp1164, Arg1227, Asp1231, and Tyr1235, indicating that compound **9** occupies the same functional region of the binding cleft and mimics the anchoring strategy of reference inhibitors.

A comparable interaction pattern was observed in HER2, where compound **9** formed strong hydrophobic contacts with residues such as Leu726, Val734, Ala751, Ile767, Met774, Ser783, Arg784, Leu785, Leu796, Thr798, Leu800, Met801, and Gly804, many of which are shared with the co-crystallized ligand and erlotinib (Figure 5, Table 3). Importantly, compound **9** maintained hydrogen-bonding interactions with Thr862 and Asp863, contributing to structural stabilization toward the hinge region. Collectively, the high degree of residue overlap, the dominance of van der Waals, π–π stacking, π-alkyl interactions, and the presence of multiple conserved contacts demonstrate that compound **9** fits deeply and specifically within the ATP-binding pockets of both kinases, thereby supporting its dual c-MET/HER2 binding profile and providing a molecular basis for its superior binding scores relative to erlotinib.

To functionally validate the in silico predictions indicating that compound **9** engages cMET and HER2 within binding sites overlapping those of clinically used inhibitors, we performed A549 cell survival and clonogenic assays in the presence of FDA-approved tyrosine kinase inhibitors. We postulated that if compound **9** modulates cMET and HER2 signaling pathways, its co-administration with established inhibitors would potentiate their antiproliferative effects. The A549 lung adenocarcinoma cell line was selected for these studies based on its higher sensitivity to compound **9** in dose–response assays, as well as the well-established involvement of cMET and HER2 signaling in lung tumorigenesis.

**Figure 7:**
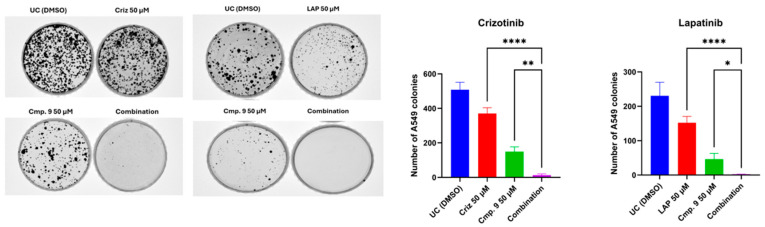
Potentiation of clonogenic growth inhibition by compound **9** in combination with cMET and HER2 inhibitors in A549 cells. A total of 5 × 10^2^ cells were seeded and exposed to crizotinib, lapatinib, compound **9**, or the indicated combinations for 12 h. Following treatment, compounds were removed and cells were cultured in fresh medium to allow colony formation. Colonies were visualized by crystal violet staining, imaged using a light transilluminator, and manually counted. Quantitative data are presented as the mean ± SD from three independent experiments. * *p* < 0.05; ** *p* < 0.01; **** *p* < 0.0001.

As shown in Figure 7, treatment with either crizotinib or lapatinib alone resulted in a reduction in colony formation relative to vehicle control, whereas compound **9** alone produced a more pronounced inhibitory effect. Notably, co-treatment with compound **9** markedly potentiated the inhibitory effects of both crizotinib and lapatinib, leading to a near-complete suppression of clonogenic growth. Quantitative analysis confirmed a statistically significant decrease in colony numbers under combination conditions compared to single-agent treatments. Collectively, these data provide functional support for the in silico docking results and suggest that compound **9** enhances the antiproliferative activity of cMET and HER2 inhibition in A549 cells.

## 3. Discussion

3-[(2,4-Difluorophenyl)amino]propanoic acid (**2**) and its derivatives, such as esters, hydrazides, hydrazones, and semicarbazides, were synthesized and characterized. Hydrazide reactions with various aromatic aldehydes and monoketones resulted in formation of hydrazones; during reactions with diketones, *β*-alanine derivatives with heterocyclic pyrazole and pyrrole rings were formed. In the present work, the synthetic approach centered on exploiting the reactivity of the *β*-alanine framework through hydrazinolysis and subsequent condensation with aromatic aldehydes and diketones to access hydrazones, and heterocyclic azoles derivatives, a strategy widely employed for obtaining structurally diverse bioactive scaffolds in medicinal chemistry. Numerous studies have shown that hydrazide and hydrazone chemistry provides a versatile and effective route to heterocyclic scaffolds with antibacterial, anticancer, and diverse pharmacological properties. For example, hydrazones have been synthesized and evaluated for anticancer and anti-inflammatory potential, demonstrating significant structure–activity relationships related to heterocyclic motifs [48,49,50]. Additionally, pyrazoles derivatives have been successfully designed as selective bacterial MetAP inhibitors, underlining the versatility of this synthetic methodology in drug discovery [51].

Following the synthesis of a library of *β*-alanine derivatives bearing 2,4-difluorophenyl, hydrazone, and diverse heterocyclic or aromatic substituents, we evaluated their in vitro antiproliferative activity against A549 (human lung adenocarcinoma) and Caco-2 (human colorectal adenocarcinoma) cell lines. In this context, the *β*-alanine linker and hydrazone functionality were selected as modular elements that can orient the 2,4-difluorophenyl ring and the heterocyclic/aromatic “head groups” within kinase binding pockets, while the fluorinated aryl fragment was expected to enhance lipophilicity, metabolic stability, and fitting into hydrophobic subpockets of growth factor receptors, as reported for other fluorinated hydrazone and Schiff base derivatives [52,53].

The anticancer activity of these *β*-alanine hydrazones is strongly structure-dependent and cell-line dependent. Early analogues **2**–**4**, lacking strongly electron-withdrawing or heteroaromatic fragments, were essentially inactive in both A549 and Caco-2 cells, suggesting that the base scaffold alone is insufficient to drive antiproliferative activity. Introduction of heteroaromatic moieties such as 2-thienyl (**5a**) and especially nitro-/halofuran substituents (**6** series) markedly improved activity, consistent with literature showing that electron-deficient heterocycles and nitro-substituted rings can enhance antiproliferative activity in hydrazone series [54,55,56]. The strong activity of **6b** bearing a 5-nitro-2-furyl group observed in Caco-2 cells suggests that both the nitro group and substitution on the furan ring are critical for optimal interaction with the biological cellular targets. By contrast, 5-bromo-2-furyl substitution (**6c**) resulted in diminished activity in both cell lines, indicating that simple halogen replacement does not recapitulate the electronic and hydrogen-bonding features provided by the nitro group.

Further synthetic diversification to phenyl-substituted hydrazones **7a**–**k** revealed that para-substitution with small electron-donating groups (4-CH_3_-Ph in **7f** and 4-OCH_3_-Ph in **7g**) supports robust antiproliferative effects, particularly in Caco-2 cells, whereas some halogenated analogues (4-bromophenyl in **7e**) showed biased activity toward Caco-2 over A549. These results suggest a delicate balance between electronic modulation and hydrophobic surface area in controlling anticancer activity and selectivity across epithelial cancer models. Compounds **8**–**21**, which incorporate additional structural variations, further confirmed that only a subset of substitution patterns yield >50% growth inhibition at 100 µM in either A549 (**9**, **10**, **12**, **17**) or Caco-2 (**8**, **9**, **11**, **18**, **19**), showing significant SAR latitude within this novel *β*-alanine–hydrazone–2,4-difluorophenyl framework. Importantly, our in silico molecular modeling indicates that compound **9** is capable of engaging HER2 and c-MET receptor tyrosine kinases, occupying the ATP-binding cleft and forming stabilizing hydrogen bonds with hinge-region residues alongside hydrophobic contacts mediated by the 2,4-difluorophenyl ring and heteroaromatic moieties. Dual or multi-target inhibition of growth factor receptors such as EGFR, HER2, and c-MET is an increasingly validated strategy to overcome resistance and pathway redundancy in solid tumors, and several hydrazone-based or heterocycle-rich scaffolds have recently been reported as potent EGFR/HER2 or EGFR/c-MET inhibitors with low-micromolar or submicromolar potency in A549 and other tumor cell lines [55]. In this context, the observed antiproliferative effects of **6b**, **7f**, **7g**, and **9**, together with the docking-supported interaction of **9** with HER2 and c-MET, support the notion that *β*-alanine-linked 2,4-difluorophenyl hydrazones represent a promising and synthetically accessible scaffold for further medicinal chemistry optimization toward growth factor receptor-driven cancers.

This study has several caveats that could be further addressed in future work. First, although the compound series demonstrates clear structure–activity trends, the chemical space explored remains relatively narrow; broader substituent synthesis—particularly incorporating medicinally preferred heterocycles, fluorinated benzene variants, and fluorine-containing spirocyclic motifs—may yield stronger target engagement and drug-like properties. Furthermore, because cytotoxicity in non-malignant HEK293 cells was evaluated at a single concentration, the present study does not permit calculation of selectivity indices. Comprehensive dose–response analysis in healthy cells will be required in future studies to quantitatively assess therapeutic windows. Second, sub-library development guided by structural modeling could allow more precise optimization toward hydrophobic pockets within HER2 and c-MET kinase domains. Third, the current conclusions rely primarily on cell viability and docking data; therefore, more in-depth functional target validation through techniques such as isothermal titration calorimetry, thermal shift assays, or surface plasmon resonance is required to confirm direct binding. Finally, assessing compound activity against clinically relevant HER2 and c-MET mutations associated with drug resistance would strengthen the translational relevance of this scaffold.

## 4. Materials and Methods

### 4.1. Chemical Synthesis

Reagents and solvents were obtained from Sigma–Aldrich (St. Louis, MO, USA) and used without further purification. The reaction course and purity of the synthesized compounds were monitored by TLC using aluminum plates precoated with Silica gel with F254 nm (Merck KGaA, Darmstadt, Germany). Melting points were determined with a B-540 melting point analyzer (Büchi Corporation, New Castle, DE, USA) and were uncorrected. NMR spectra were recorded on a Brucker Avance III (400, 101 MHz) spectrometer (Bruker BioSpin AG, Fällanden, Switzerland). Chemical shifts were reported in (δ) ppm relative to tetramethylsilane (TMS) with the residual solvent as internal reference (DMSO-d_6_, δ = 2.50 ppm for ^1^H and δ = 39.52 ppm for ^13^C). Data were reported as follows: chemical shift, multiplicity, coupling constant (Hz), integration, and assignment. IR spectra (ν, cm^−1^) were recorded on a Perkin–Elmer Frontier spectrometer (Perkin–Elmer Inc., Waltham, MA, USA) in wave interval from 4000 to 560 cm^−1^, by pressing a small amount of sample against a diamond crystal plate (number of scans—6, resolution—4 cm^−1^), data were processed using Spectrum software (Version 10.03.06). Elemental analyses (C, H, N) were conducted using the Elemental Analyzer CE-440 (Exeter Analytical, Inc., Chelmsford, MA, USA); their results were found to be in good agreement (±0.3%) with the calculated values. Mass spectra were obtained on Ms. Agilent 6530A QTOF, HPLC (Agilent 1260 II series, Santa Clara, CA, USA) spectrometer.

#### 4.1.1. 3-[(2,4-Difluorophenyl)Amino]Propanoic Acid (**2**)

A mixture of 2,4-difluoroaniline **(1)** (10.00 g, 77.5 mmol), acrylic acid (8.32 g, 115.5 mmol) and toluene (15 mL) was refluxed for 24 h and then cooled. Later aqueous 5% sodium hydroxide solution (100 mL) was added and after mixing aqueous layer was separated. It was acidified using acetic acid to pH 5. Obtained crude solids were purified by dissolving them in 5% sodium hydroxide solution (80 mL), the solution was filtered off, and the filtrate was acidified with acetic acid to pH 5. Formed crystals were once again filtered, washed with distilled water (3 mL) and dried obtaining the starting compound **2**. A Bunsen flask and a porcelain funnel with filter paper were used for filtration.

Purple solid, yield 10.07 g, 65%, m. p. 76–78 °C. **FT-IR**: ν 3437 (NH); 3078 (OH); 1683 (C=O) cm^−1^. **^1^H NMR** (400 MHz, DMSO-*d*_6_) δ 2.51–2.61 (m, 2H, NHCH_2_ overlaps with DMSO-*d*_6_), 3.27 (t, 2H, *J* = 5.0 Hz, CH_2_CO), 5.25 (s, 1H, NH), 6.66–6.79 (m, 1H, H_Ar_), 6.86 (t, 1H, *J* = 8.7 Hz, H_Ar_), 7.01–7.12 (m, 1H, H_Ar_), 12.26 (br. s., 1H, COOH) ppm. **^13^C NMR** (101 MHz, DMSO-*d*_6_) δ 33.6 (NHCH_2_), 39.0 (CH_2_CO overlaps with DMSO-*d*_6_), 103.2, 103.4, 103.4, 103.7, 110.7, 110.7, 110.9, 111.0, 111.9, 112.0, 112.1, 133.4, 133.4, 133.5, 133.5, 148.9, 149.0, 151.3, 151.4, 151.7, 151.8, 154.0, 154.1 (C_Ar_); 173.3 (C=O) ppm. **Calcd. for** C_9_H_9_F_2_NO_2_, %: C 53.73; H 4.51; N 6.96; Found, %: C 53.54; H 4.34; N 6.80. **HRMS** *m/z* calcd. for C_9_H_9_F_2_NO_2_ [M + H]^+^: 202.0601, Found 202.1083.

#### 4.1.2. Methyl 3-[(2,4-Difluorophenyl)Amino]Propanoate (**3**)

Ester **3** was obtained by dissolving starting material **2** (18.00 g, 89.5 mmol) in methanol (330 mL) and adding 80 drops of concentrated sulfuric acid, which was used as a catalyst. The reaction mixture was refluxed for 5 h, later the solvent was distilled under reduced pressure. The residue was neutralized using 5% sodium carbonate solution (50 mL). The product was purified by extraction using ethyl acetate (50 mL); residual water was removed by adding sodium sulfate (1.2 g). After filtration, organic layer was distilled under pressure, the obtained product **3** is in a liquid state. A Bunsen flask and a porcelain funnel with filter paper were used for filtration.

Dark purple liquid, yield 16.91 g, 93%. **^1^H NMR** (400 MHz, DMSO-*d*_6_) δ 2.60 (t, 2H, *J* = 6.8 Hz, NHCH_2_), 3.24–3.36 (m, 2H, CH_2_CO), 3.60 (s, 3H, CH_3_), 5.31 (s, 1H, NH), 6.72 (dd, 1H, *J* = 15.1, 9.2 Hz, H_Ar_), 6.86 (t, 1H, *J* = 8.6 Hz, H_Ar_), 7.07 (t, 1H, *J* = 8.7 Hz, H_Ar_) ppm. **^13^C NMR** (101 MHz, DMSO-*d*_6_) δ 33.3 (NHCH_2_), 38.9 (CH_2_CO overlaps with DMSO-*d*_6_), 51.4 (CH_3_), 103.2, 103.4, 103.5, 103.7, 110.6, 110.7, 110.9, 110.9, 111.9, 111.9, 112.0, 133.3, 133.3, 133.4, 133.4, 148.9, 149.0, 151.3, 151.4, 151.8, 151.8, 154.1, 154.2 (C_Ar_), 172.1 (C=O) ppm. **HRMS** *m/z* calcd. for C_10_H_11_F_2_NO_2_ [M + H]^+^: 216.0758, Found: 216.0827.

#### 4.1.3. 3-[(2,4-Difluorophenyl)Amino]Propanehydrazide (**4**)

Ester **3** (5.88 g, 27.3 mmol) was dissolved in 2-propanol (50 mL), hydrazine monohydrate (5.41 g, 108.1 mmol) was added dropwise. The reaction mixture was refluxed for 20 h, then cooled down. The obtained solids were filtered off, washed (2 mL) and recrystallized (10 mL) with 2-propanol to give the title compound **4**. A Bunsen flask and a porcelain funnel with filter paper were used for filtration.

White solid, yield 4.88 g, 82%, m. p. 139–141 °C (from 2-propanol). **FT-IR**: ν 3304 (2x NH+ NH_2_); 1642 (C=O) cm^−1^. **^1^H NMR** (400 MHz, DMSO-*d*_6_) δ 2.33 (t, 2H, *J* = 6.9 Hz, NHCH_2_), 3.27 (q, 2H, *J* = 6.5 Hz, CH_2_CO), 4.20 (s, 2H, NH_2_), 5.27 (s, 1H, NH), 6.66–6.78 (m, 1H, H_Ar_), 6.87 (t, 1H, *J* = 8.5 Hz, H_Ar_), 7.00–7.13 (m, 1H, H_Ar_), 9.06 (s, 2H, NH) ppm. **^13^C NMR** (101 MHz, DMSO-*d*_6_) δ 33.1 (NHCH_2_), 39.7 (CH_2_CO overlaps with DMSO-*d*_6_), 103.1, 103.4, 103.4, 103.6, 110.6, 110.7, 110.9, 110.9, 111.8, 111.9, 133.4, 133.4, 133.5, 133.6, 148.9, 148.7, 151.2, 151.4, 151.7, 151.8, 154.0, 154.1 (C_Ar_), 170.2 (C=O) ppm. **Calcd.** for C_9_H_11_F_2_N_3_O, %: C 50.23; H 5.15; N 19.53; Found, %: C 50.12; H 4.99; N 19.39. **HRMS** *m/z* calcd. for C_9_H_11_F_2_N_3_O_2_ [M + H]^+^: 216.0870, Found: 216.1412.

**General procedure for the preparation of hydrazones 5a** **–** **c, 6a** **–** **c, 7a** **–** **k, 8 and 9**

To a solution of hydrazide **4** (0.40 g, 1.9 mmol) in 2-propanol (15 mL), the corresponding aromatic or heterocyclic aldehyde (2.3 mmol) was added and the mixture was heated at reflux for 1 h (2 h for compounds **8** and **9**). When reaction solution was cooled, the obtained solids were filtered, dried and purified by recrystallization from 2-propanol (10 mL). A Bunsen flask and a porcelain funnel with filter paper were used for filtration. As previously mentioned, hydrazones **5a**–**c**, **6a**–**c**, **7a**–**k**, **8**, **9** and **14**, **15** in DMSO-*d*_6_ solution exist as a mixture of *E/Z* isomers as indicated by the proton signals of azomethine fragment and the amide -NH group in ^1^H NMR spectra, each of which is visible as a set of two singlets with a signal intensity ratio of 60:40 [43].

#### 4.1.4. 3-[(2,4-Difluorophenyl)Amino]-N’-(Thiophen-2-Ylmethylene)Propanehydrazide (**5a**)

Light grey solid, yield 0.53 g, 91%, m. p. 167–169 °C (from 2-propanol). **FT-IR:** ν 3330, 3080 (2x NH), 1651 (C=O), 1524 (C=N) cm^−1^. **^1^H NMR** (400 MHz, DMSO-*d*_6_) δ (Z/E 60/40): 2.43–2.45 and 2.84 (m and t, 2H, *J* = 7.1 Hz, NHCH_2_ overlaps with DMSO-*d*_6_), 3.28–3.43 (m, 2H, CH_2_CO), 5.29–5.42 (m, 1H, NHCH_2_), 6.71–6.84 (m, 1H, H_Ar_), 6.85–6.94 (m, 1H, H_Ar_), 7.01–7.16 (m, 2H, H_Ar_), 7.40 (dd, 1H, *J* = 14.2, 3.2 Hz, H_Ar_), 7.62 (t, 1H, *J* = 8.4 Hz, H_Ar_), 8.18 and 8.38 (2s, 1H, NHNCH), 11.33 and 11.36 (2s, 1H, CONH) ppm. **^13^C NMR** (101 MHz, DMSO-*d*_6_) δ 31.9 (NHCH_2_), 33.9 (CH_2_CO), 103.2, 103.4, 103.4, 103.7, 110.7, 110.7, 110.9, 110.9, 111.7, 127.8, 127.9, 128.3, 128.7, 130.3, 130.7, 138.2, 138.9, 139.1, 141.3, 148.9, 149.0, 151.3, 151.4, 151.6, 151.7, 153.9, 154.1 (C_Ar_), 167.2 (C=N), 172.6 (C=O) ppm. **Calcd. for** C_14_H_13_F_2_N_3_OS, %: C 54.36; H 4.24; N 13.58; Found, %: C 54.25; H 4.05; N 13.44. **HRMS** *m/z* calcd. for C_14_H_13_F_2_N_3_OS [M + H]^+^: 310.0747, Found: 310.1411.

#### 4.1.5. 3-[(2,4-Difluorophenyl)Amino]-N’-[(5-Nitrothiophen-2-yl)Methylene]Propanehydrazide (**5b**)

Yellow solid, yield 0.61 g, 91%, m. p. 171–173 °C (from 2-propanol). **FT-IR**: ν 3383, 3111 (2x NH), 1662 (C=O), 1522 (C=N) cm^−1^. **^1^H NMR** (400 MHz, DMSO-*d*_6_) δ (Z/E 60/40): 2.50–2.58 (m, 1H, NHCH_2_ overlaps with DMSO-*d*_6_), 2.89 (t, 1H, *J* = 7.0 Hz, NHCH_2_), 3.33–3.42 (m, 2H, CH_2_CO), 5.32–5.44 (m, 1H, NHCH_2_), 6.72–6.83 (m, 1H, H_Ar_), 6.89 (t, 1H, *J* = 8.5 Hz, H_Ar_), 7.01–7.13 (m, 1H, H_Ar_), 7.51 (dd, 1H, *J* = 15.5, 4.2 Hz, H_Ar_), 8.06–8.13 (m, 1H, H_Ar_), 8.16 ir 8.43 (2s, 1H, NHNCH), 11.77 and 11.78 (2s, 1H, CONH) ppm. **^13^C NMR** (101 MHz, DMSO-*d*_6_) δ 31.7 (NHCH_2_), 34.0 (CH_2_CO), 103.2, 103.4, 103.5, 103.7, 110.6, 110.7, 110.8, 110.9, 111.7, 129.0, 129.5, 130.5, 130.6, 133.5, 136.3, 139.7, 146.7, 146.9, 148.9, 150.3, 150.7, 151.3, 151.6 (C_Ar_), 167.9 (C=N), 173.3 (C=O) ppm. **Calcd. for** C_14_H_12_F_2_N_4_O_3_S, %: C 47.46; H 3.41; N 15.81; Found, %: C 47.30; H 3.30; N 15.60. **HRMS** *m/z* calcd. for C_14_H_12_F_2_N_4_O_3_S [M + H]^+^: 355.0598, Found: 355.1128.

#### 4.1.6. N’-[(4-Bromothiophen-2-yl)Methylene]-3-[(2,4-Difluorophenyl)Amino]Propanehydrazide (**5c**)

Light grey solid, yield 0.67 g, 90%, m. p. 176–178 °C (from 2-propanol). **FT-IR**: ν 3299, 3096 (2x NH), 1678 (C=O), 1520 (C=N) cm^−1^. **^1^H NMR** (400 MHz, DMSO-*d*_6_) δ (Z/E 60/40): 2.44–2.54 (m, 1H, NHCH_2_ overlaps with DMSO-*d*_6_), 2.84 (t, 1H, *J* = 7.0 Hz, NHCH_2_), 3.29–3.40 (m, 2H, CH_2_CO), 5.29–5.40 (m, 1H, NHCH_2_), 6.71–6.82 (m, 1H, H_Ar_), 6.88 (t, 1H, *J* = 8.6 Hz, H_Ar_), 7.01–7.12 (m, 1H, H_Ar_), 7.42–7.51 (m, 1H, H_Ar_), 7.69–7.76 (m, 1H, H_Ar_), 8.12 and 8.34 (2s, 1H, NHNCH), 11.45 and 11.50 (2s, 1H, CONH) ppm. **^13^C NMR** (101 MHz, DMSO-*d*_6_) δ 31.8 (NHCH_2_), 33.9 (CH_2_CO), 103.2, 103.4, 103.5, 103.7, 109.2, 109.2, 110.7, 110.7, 110.9, 110.9, 125.5, 126.0, 131.6, 132.2, 136.7, 139.9, 140.4, 140.6, 148.9, 151.3, 151.4, 151.7, 151.8, 154.0, 154.1, 154.1 (C_Ar_), 167.4 (C=N), 172.8 (C=O) ppm. **Calcd. for** C_14_H_12_BrF_2_N_3_OS, %: C 43.31; H 3.12; N 10.82; Found, %: C 43.36; H 3.07; N 10.64. **HRMS** *m/z* calcd. for C_14_H_12_BrF_2_N_3_OS [M + H]^+^: 387.9853, Found: 388.0249.

#### 4.1.7. 3-[(2,4-Difluorophenyl)Amino]-N’-(Furan-2-Ylmethylene)Propanehydrazide (**6a**)

Grey solid, yield 0.46 g, 81%, m. p. 163–165 °C (from 2-propanol). **FT-IR**: ν 3339, 3060 (2x NH), 1671 (C=O), 1525 (C=N) cm^−1^. **^1^H NMR** (400 MHz, DMSO-*d*_6_) δ (Z/E 60/40): 2.44–2.56 (m, 1H, NHCH_2_ overlaps with DMSO-*d*_6_), 2.84 (t, 1H, *J* = 7.1 Hz, NHCH_2_), 3.29–3.42 (m, 2H, CH_2_CO), 5.29–5.42 (m, 1H, NHCH_2_), 6.61 (s, 1H, H_Ar_), 6.72–6.93 (m, 3H, H_Ar_), 7.02–7.12 (m, 1H, H_Ar_), 7.82 (s, 1H, H_Ar_), 7.88 and 8.05 (2s, 1H, NHNCH), 11.30 and 11.35 (2s, 1H, CONH) ppm. **^13^C NMR** (101 MHz, DMSO-*d*_6_) δ 32.0 (NHCH_2_), 33.9 (CH_2_CO), 103.2, 103.4, 103.4, 103.7, 103.7, 110.7, 110.7, 110.9, 110.9, 111.9, 112.1, 112.1, 113.3, 113.3, 133.1, 133.4, 133.5, 133.5, 136.0, 144.9, 145.1, 148.9, 149.0, 149.2, 149.4, 151.3, 151.4, 151.7, 153.9, 154.1 (C_Ar_), 167.2 (C=N), 172,8 (C=O) ppm. **Calcd. for** C_14_H_13_F_2_N_3_O_2_, %: C 57.54; H 4.47; N 14.33; Found, %: C 57.69; H 4.64; N 14.21. **HRMS** *m/z* calcd. for C_14_H_13_F_2_N_3_O_2_ [M + H]^+^: 294.0976, Found: 294.1648.

#### 4.1.8. 3-[(2,4-Difluorophenyl)Amino]-N’-[(5-Nitrofuran-2-yl)Methylene]Propanehydrazide (**6b**)

Yellow solid, yield 0.57 g, 89%, m. p. 181–183 °C (from 2-propanol). **FT-IR**: ν 3382, 3107 (2x NH), 1665 (C=O), 1515 (C=N) cm^−1^. **^1^H NMR** (400 MHz, DMSO-*d*_6_) δ (Z/E 60/40): 2.55 (t, 1H, *J* = 6.8 Hz, NHCH_2_ overlaps with DMSO-*d*_6_), 2.91 (t, 1H, *J* = 6.9 Hz, NHCH_2_), 3.35–3.43 (m, 2H, CH_2_CO), 5.31–5.41 (m, 1H, NHCH_2_), 6.72–6.83 (m, 1H, H_Ar_), 6.88 (t, 1H, *J* = 8.5 Hz, H_Ar_), 7.00–7.12 (m, 1H, H_Ar_), 7.15–7.23 (m, 1H, H_Ar_), 7.73–7.81 (m, 1H, H_Ar_), 7.93 and 8.13 (2s, 1H, NHNCH), 11.75 and 11.80 (2s, 1H, CONH) ppm. **^13^C NMR** (101 MHz, DMSO-*d*_6_) δ 32.2 (NHCH_2_), 34.5 (CH_2_CO), 103.6, 103.6, 103.8, 103.9, 104.1, 104.1, 104.7, 111.2, 111.4, 112.4, 112.4, 112.5, 115.1, 115.2, 115.29, 115.6, 131.6, 133.8, 133.8, 133.8, 133.9, 133.9, 134.5, 149.4, 151.8, 152.1, 152.2, 152.2, 152.3, 152.3, 154.4 (C_Ar_), 168.4 (C=N), 174.0 (C=O) ppm. **Calcd. for** C_14_H_12_F_2_N_4_O_4_, %: C 49.71; H 3.58; N 16.56; Found, %: C 49.93; H 3.71; N 16.38. **HRMS** *m/z* calcd. for C_14_H_12_F_2_N_4_O_4_ [M + H]^+^: 339.0827, Found: 339.1425.

#### 4.1.9. N’-[(5-Bromofuran-2-yl)Methylene]-3-[(2,4-Difluorophenyl)Amino]Propanehydrazide (**6c**)

Grey solid, yield 0.59 g, 83%, m. p. 170–172 °C (from 2-propanol). **FT-IR**: ν 3333, 3125 (2x NH), 1649 (C=O), 1524 (C=N) cm^−1^. **^1^H NMR** (400 MHz, DMSO-*d*_6_) δ (Z/E 60/40): 2.41–2.55 (m, 1H, NHCH_2_ overlaps with DMSO-*d*_6_), 2.85 (t, 1H, *J* = 7.1 Hz, NHCH_2_), 3.26–3.43 (m, 2H, CH_2_CO), 5.29–5.41 (m, 1H, NHCH_2_), 6.67–6.95 (m, 4H, H_Ar_), 7.01–7.13 (m, 1H, H_Ar_), 7.80 and 7.97 (2s, 1H, NHNCH), 11.36 and 11.42 (2s, 1H, CONH) ppm. **^13^C NMR** (101 MHz, DMSO-*d*_6_) δ 31.9 (NHCH_2_), 33.9 (CH_2_CO), 103.2, 103.4, 103.4, 103.7, 110.7, 110.9, 114.2, 114.2, 115.9, 116.0, 124.2, 124.4, 132.1, 133.3, 133.5, 134.9, 148.9, 149.0, 151.2, 151.3, 151.4, 151.6, 151.7, 153.9, 154.0 (C_Ar_), 167.4 (C=N), 172.9 (C=O) ppm. **Calcd. for** C_14_H_12_BrF_2_N_3_O_2_, %: C 45.18; H 3.25; N 11.29; Found, %: C 45.38; H 3.18; N 11.26. **HRMS** *m/z* calcd. for C_14_H_12_BrF_2_N_3_O_2_ [M + H]^+^: 372.0081, Found: 372.0548.

#### 4.1.10. N’-Benzylidene-3-[(2,4-Difluorophenyl)Amino]Propanehydrazide (**7a**)

Light purple solid, yield 0.51 g, 89%, m. p. 195–197 °C (from 2-propanol). **FT-IR**: ν 3325, 3062 (2x NH), 1670 (C=O), 1525 (C=N) cm^−1^. **^1^H NMR** (400 MHz, DMSO-*d*_6_) δ (Z/E 60/40): 2.45–2.56 (m, 1H, NHCH_2_ overlaps with DMSO-*d*_6_), 2.94 (t, 1H, *J* = 6.9 Hz, NHCH_2_), 3.30–3.44 (m, 2H, CH_2_CO), 5.30–5.41 (m, 1H, NHCH_2_), 6.71–6.82 (m, 1H, H_Ar_), 6.83–6.93 (m, 1H, H_Ar_), 7.00–7.13 (m, 1H, H_Ar_), 7.34–7.48 (m, 3H, H_Ar_), 7.57–7.72 (m, 2H, H_Ar_), 8.00 and 8.17 (2s, 1H, NHNCH), 11.36 and 11.42 (2s, 1H, CONH) ppm. **^13^C NMR** (101 MHz, DMSO-*d*_6_) δ 31.9 (NHCH_2_), 33.9 (CH_2_CO), 103.2, 103.4, 103.4, 103.7, 110.6, 110.8, 126.7, 127.0, 128.8, 129.7, 129.9, 134.2, 134.3, 142.9, 146.1, 148.9, 149.0, 151.3, 151.4, 151.6, 151.7, 151.7, 153.9, 154.0, 154.1 (C_Ar_), 167.3 (C=N), 173.0 (C=O) ppm. **Calcd. for** C_16_H_15_F_2_N_3_O, %: C 63.36; H 4.98; N 13.85; Found, %: C 63.46; H 5.04; N 13.75. **HRMS** *m/z* calcd. for C_16_H_15_F_2_N_3_O [M + H]^+^: 304.1183, Found: 304.1850.

#### 4.1.11. 3-[(2,4-Difluorophenyl)Amino]-N’-(4-Fluorobenzylidene)Propanehydrazide (**7b**)

Light purple solid, yield 0.57 g, 93%, m. p. 196–198 °C (from 2-propanol). **FT-IR**: ν 3322, 3066 (2x NH), 1670 (C=O), 1515 (C=N) cm^−1^. **^1^H NMR** (400 MHz, DMSO-*d*_6_) δ (Z/E 60/40): 2.45–2.57 (m, 1H, NHCH_2_ overlaps with DMSO-*d*_6_), 2.94 (t, 1H, *J* = 6.9 Hz, NHCH_2_), 3.31–3.44 (m, 2H, CH_2_CO), 5.35 (s, 1H, NHCH_2_), 6.71–6.82 (m, 1H, H_Ar_), 6.83–6.93 (m, 1H, H_Ar_), 7.00–7.13 (m, 1H, H_Ar_), 7.27 (dd, 2H, *J* = 15.0, 4.2 Hz, H_Ar_), 7.64–7.78 (m, 2H, H_Ar_), 7.99 and 8.17 (2s, 1H, NHNCH), 11.37 and 11.56 (2s, 1H, CONH) ppm. **^13^C NMR** (101 MHz, DMSO-*d*_6_) δ 31.8 (NHCH_2_), 33.9 (CH_2_CO), 103.2, 103.4, 103.4, 103.7, 110.7, 110.9, 111.7, 115.7, 115.9, 116.0, 128.8, 128.9, 129.1, 129.2, 130.9, 141.8, 145.0, 149.0, 149.0, 151.3, 151.4, 151.6, 151.7, 154.1, 161.7, 161.8, 164.1, 164.3 (C_Ar_), 167.3 (C=N), 173.1 (C=O) ppm. **Calcd. for** C_16_H_14_F_3_N_3_O, %: C 59.81; H 4.39; N 13.08; Found, %: C 60.06; H 4.53; N 13.01. **HRMS** *m/z* calcd. for C_16_H_14_F_3_N_3_O [M + H]^+^: 322.1089, Found: 322.1722.

#### 4.1.12. N’-(2,4-Difluorobenzylidene)-3-[(2,4-Difluorophenyl)Amino]Propanehydrazide (**7c**)

Light purple solid, yield 0.50 g, 78%, m. p. 140–142 °C (from 2-propanol). **FT-IR**: ν 3321, 3070 (2x NH), 1654 (C=O), 1512 (C=N) cm^−1^. **^1^H NMR** (400 MHz, DMSO-*d*_6_) δ (Z/E 60/40): 2.45–2.57 (m, 1H, NHCH_2_ overlaps with DMSO-*d*_6_), 2.94 (t, 1H, *J* = 6.9 Hz, NHCH_2_), 3.31–3.44 (m, 2H, CH_2_CO), 5.28–5.40 (m, 1H, NHCH_2_), 6.71–6.82 (m, 1H, H_Ar_), 6.83–6.93 (m, 1H, H_Ar_), 7.00–7.11 (m, 1H, H_Ar_), 7.12–7.23 (m, 1H, H_Ar_), 7.34 (t, 1H, *J* = 8.6 Hz, H_Ar_), 7.80–7.96 (m, 1H, H_Ar_), 8.14 and 8.34 (2s, 1H, NHNCH), 11.47 and 11.56 (2s, 1H, CONH) ppm. **^13^C NMR** (101 MHz, DMSO-*d*_6_) δ 31.1 (NHCH_2_), 33.3 (CH_2_CO), 102.5, 102.8, 102.8, 103.0, 103.6, 103.8, 104.1, 110.0, 110.2, 118.0, 118.1, 127.1, 132.8, 132.9, 134.3, 134.4, 148.2, 148.4, 150.6, 150.8, 151.0, 153.4, 158.9, 161.1, 161.4 (C_Ar_), 166.8 (C=N), 172.6 (C=O) ppm. **Calcd. for** C_16_H_13_F_4_N_3_O, %: C 56.64; H 3.86; N 12.38; Found, %: C 56.46; H 3.91; N 12.24. **HRMS** *m/z* calcd. for C_16_H_13_F_4_N_3_O [M + H]^+^: 340.0995, Found: 340.1586.

#### 4.1.13. N’-(4-Chlorobenzylidene)-3-[(2,4-Difluorophenyl)Amino]Propanehydrazide (**7d**)

White solid, yield 0.55 g, 86%, m. p. 150–152 °C (from 2-propanol). **FT-IR**: ν 3323, 3064 (2x NH), 1667 (C=O), 1526 (C=N) cm^−1^. **^1^H NMR** (400 MHz, DMSO-*d*_6_) δ (Z/E 60/40): 2.45–2.57 (m, 1H, overlaps with DMSO-*d*_6_), 2.94 (t, 1H, *J* = 7.0 Hz, NHCH_2_), 3.29–3.43 (m, 2H, CH_2_CO), 5.30–5.39 (m, 1H, NHCH_2_), 6.72–6.82 (m, 1H, H_Ar_), 6.83–6.93 (m, 1H, H_Ar_), 7.01–7.13 (m, 1H, H_Ar_), 7.50 (t, 2H, *J* = 7.8 Hz, H_Ar_), 7.61–7.75 (m, 2H, H_Ar_), 7.98 and 8.16 (2s, 1H, NHNCH), 11.43 and 11.49 (2s, 1H, CONH) ppm. **^13^C NMR** (101 MHz, DMSO-*d*_6_) δ 31.8 (NHCH_2_), 33.9 (CH_2_CO), 103.2, 103.4, 103.7, 110.7, 110.8, 110.9, 111.7, 128.3, 128.6, 128.9, 128.9, 133.2, 141.6, 144.7, 144.8, 148.9, 148.9, 149.0, 151.3, 151.4, 151.4 (C_Ar_), 167.4 (C=N), 173.1 (C=O) ppm. **Calcd. for** C_16_H_14_ClF_2_N_3_O, %: C 56.90; H 4.18; N 12.44; Found, %: C 57.15; H 4.29; N 12.29. **HRMS** *m/z* calcd. for C_16_H_14_ClF_2_N_3_O [M + H]^+^: 338.0793, Found: 338.1381.

#### 4.1.14. N’-(4-Bromobenzylidene)-3-[(2,4-Difluorophenyl)Amino]Propanehydrazide (**7e**)

White solid, yield 0.63 g, 86%, m. p. 166–168 °C (from 2-propanol). **FT-IR**: ν 3321, 3064 (2x NH), 1668 (C=O), 1526 (C=N) cm^−1^. **^1^H NMR** (400 MHz, DMSO-*d*_6_) δ (Z/E 60/40): 2.43–2.56 (m, 1H, NHCH_2_ overlaps with DMSO-*d*_6_), 2.93 (t, 1H, *J* = 6.9 Hz, NHCH_2_), 3.30–3.43 (m, 2H, CH_2_CO), 5.34 (s, 1H, NHCH_2_), 6.71–6.81 (m, 1H, H_Ar_), 6.82–6.92 (m, 1H, H_Ar_), 7.00–7.12 (m, 1H, H_Ar_), 7.52–7.67 (m, 4H, H_Ar_), 7.96 and 8.14 (2s, 1H, NHNCH), 11.42 and 11.49 (2s, 1H, CONH) ppm. **^13^C NMR** (101 MHz, DMSO-*d*_6_) δ 31.8 (NHCH_2_), 33.9 (CH_2_CO), 103.2, 103.4, 103.4, 103.7, 110.7, 110.9, 111.8, 122.9, 128.9, 131.8, 131.8, 133.5, 141.7, 144.8, 148.9, 149.0, 151.3, 151.4, 151.6, 151.7, 153.9, 154.1 (C_Ar_), 167.4 (C=N), 173.1 (C=O) ppm. **Calcd. for** C_16_H_14_BrF_2_N_3_O, %: C 50.28; H 3.69; N 10.99; Found, %: C 50.26; H 3.67; N 10.86. **HRMS** *m/z* calcd. for C_16_H_14_BrF_2_N_3_O [M + H]^+^: 382.0288, Found: 382.0713.

#### 4.1.15. 3-[(2,4-Difluorophenyl)Amino]-N’-(4-Methylbenzylidene)Propanehydrazide (**7f**)

White solid, yield 0.55 g, 91%, m. p. 185–187 °C (from 2-propanol). **FT-IR**: ν 3319, 3076 (2x NH), 1670 (C=O), 1526 (C=N) cm^−1^. **^1^H NMR** (400 MHz, DMSO-*d*_6_) δ (Z/E 60/40): 2.33 (s, 3H, CH_3_), 2.43–2.55 (m, 1H, NHCH_2_ overlaps with DMSO-*d*_6_), 2.92 (t, 1H, *J* = 7.0 Hz, NHCH_2_), 3.29–3.42 (m, 2H, CH_2_CO), 5.30–5.39 (m, 1H, NHCH_2_), 6.73–6.82 (m, 1H, H_Ar_), 6.83–6.93 (m, 1H, H_Ar_), 7.01–7.12 (m, 1H, H_Ar_), 7.19–7.28 (m, 2H, H_Ar_), 7.49–7.60 (m, 2H, H_Ar_), 7.96 and 8.12 (2s, 1H, NHNCH), 11.29 and 11.35 (2s, 1H, CONH) ppm. **^13^C NMR** (101 MHz, DMSO-*d*_6_) δ 21.0 (CH_3_), 31.8 (NHCH_2_), 33.9 (CH_2_CO), 103.2, 103.4, 103.5, 103.7, 110.6, 110.8, 110.9, 111.8, 126.7, 127.0, 129.4, 131.5, 131.6, 133.6, 139.5, 139.6, 139.7, 143.0, 146.1, 148.9, 149.0, 151.3, 151.4, 153.9 (C_Ar_), 167.2 (C=N), 172.9 (C=O) ppm. **Calcd. for** C_17_H_17_F_2_N_3_O, %: C 64.34; H 5.40; N 13.24; Found, %: C 64.21; H 5.31; N 13.29. **HRMS** *m/z* calcd. for C_17_H_17_F_2_N_3_O [M + H]^+^: 318.1340, Found: 318.1975.

#### 4.1.16. 3-[(2,4-Difluorophenyl)Amino]-N’-(4-Methoxybenzylidene)Propanehydrazide (**7g**)

Light purple solid, yield 0.56 g, 87%, m. p. 170–172 °C (from 2-propanol). **FT-IR**: ν 3320, 3064 (2x NH), 1670 (C=O), 1516 (C=N) cm^−1^. **^1^H NMR** (400 MHz, DMSO-*d*_6_) δ (Z/E 60/40): 2.42–2.53 (m, 1H, NHCH_2_ overlaps with DMSO-*d*_6_), 2.91 (t, 1H, *J* = 7.0 Hz, NHCH_2_), 3.30–3.42 (m, 2H, CH_2_CO), 3.79 (s, 3H, OCH_3_), 5.29–5.38 (m, 1H, NHCH_2_), 6.72–6.82 (m, 1H, H_Ar_), 6.83–6.92 (m, 1H, H_Ar_), 6.93–7.02 (m, 2H, H_Ar_), 7.03–7.14 (m, 1H, H_Ar_), 7.53–7.65 (m, 2H, H_Ar_), 7.94 and 8.10 (2s, 1H, NHNCH), 11.23 and 11.28 (2s, 1H, CONH) ppm. **^13^C NMR** (101 MHz, DMSO-*d*_6_) 31.8 (NHCH_2_), 33.9 (CH_2_CO), 55.3 (OCH_3_), 103.2, 103.4, 103.4, 103.7, 110.7, 110.8, 110.9, 114.3, 126.8, 128.2, 128.6, 133.5, 133.6, 142.8, 146.0, 148.9, 149.0, 151.3, 161.0, 160.7 (C_Ar_), 167.0 (C=N), 172.8 (C=O) ppm. **Calcd. for** C_17_H_17_F_2_N_3_O_2_, %: C 61.26; H 5.14; N 12.61; Found, %: C 61.22; H 5.34; N 12.43. **HRMS** *m/z* calcd. for C_17_H_17_F_2_N_3_O_2_ [M + H]^+^: 334.1289, Found: 334.1895.

#### 4.1.17. 3-[(2,4-Difluorophenyl)Amino]-N’-(3,4,5-Trimethoxybenzylidene)Propanehydrazide (**7h**)

White solid, yield 0.56 g, 75%, m. p. 170–172 °C (from 2-propanol). **FT-IR**: ν 3353, 3005 (2x NH), 1664 (C=O), 1522 (C=N) cm^−1^. **^1^H NMR** (400 MHz, DMSO-*d*_6_) δ (Z/E 60/40): 2.45–2.56 (m, 1H, NHCH_2_ overlaps with DMSO-*d*_6_), 2.95 (t, 1H, *J* = 6.9 Hz, NHCH_2_), 3.29–3.44 (m, 2H, CH_2_CO), 3.69 (s, 3H, OCH_3_), 3.76–3.86 (m, 6H, 2x OCH_3_), 5.27–5.38 (m, 1H, NHCH_2_), 6.71–6.91 (m, 2H, H_Ar_), 6.96 (d, 2H, *J* = 13.4 Hz, H_Ar_), 7.01–7.13 (m, 1H, H_Ar_), 7.91 and 8.08 (2s, 1H, NHNCH), 11.39 and 11.40 (2s, 1H, CONH) ppm. **^13^C NMR** (101 MHz, DMSO-*d*_6_) 31.8 (NHCH_2_), 33.9 (CH_2_CO), 55.9, 55.9, 60.1 (3x OCH_3_), 103.2, 103.4, 103.5, 103.7, 104.0, 104.2, 110.7, 110.9, 111.9, 129.8, 129.9, 133.4, 133.5, 133.5, 133.6, 133.7, 139.0, 139.1, 142.9, 146.1, 149.0, 151.3, 151.4, 153.2 (C_Ar_), 167.3 (C=N), 173.1 (C=O) ppm. **Calcd. for** C_19_H_21_F_2_N_3_O_4_, %: C 58.01; H 5.38; N 10.68; Found, %: C 57.84; H 5.56; N 10.58. **HRMS** *m/z* calcd. for C_19_H_21_F_2_N_3_O_4_ [M + H]^+^: 394.1500, Found: 394.1883.

#### 4.1.18. 3-[(2,4-Difluorophenyl)Amino]-N’-[4-(Dimethylamino)Benzylidene]Propanehydrazide (**7i**)

Light brown solid, yield 0.61 g, 92%, m. p. 181–183 °C (from 2-propanol). **FT-IR**: ν 3317, 3060 (2x NH), 1669 (C=O), 1525 (C=N) cm^−1^. **^1^H NMR** (400 MHz, DMSO-*d*_6_) δ (Z/E 60/40): 2.41–2.55 (m, 1H, NHCH_2_ overlaps with DMSO-*d*_6_), 2.89 (t, 1H, *J* = 7.0 Hz, NHCH_2_), 2.95 (s, 6H, 2x CH_3_), 3.30–3.42 (m, 2H, CH_2_CO), 5.29–5.39 (m, 1H, NHCH_2_), 6.65–6.82 (m, 3H, H_Ar_), 6.83–6.93 (m, 1H, H_Ar_), 7.02–7.13 (m, 1H, H_Ar_), 7.40–7.52 (m, 2H, H_Ar_), 7.86 and 8.01 (2s, 1H, NHNCH), 11.07 and 11.11 (2s, 1H, CONH) ppm. **^13^C NMR** (101 MHz, DMSO-*d*_6_) 31.9 (NHCH_2_), 33.9 (CH_2_CO), 39.0, 39.8 (2x CH_3_ overlaps with DMSO-*d*_6_), 103.2, 103.4, 103.7, 103.8, 110.9, 111.8, 111.9, 121.6, 121.6, 128.0, 128.3, 133.5, 133.6, 143.8, 146.9, 148.9, 149.0, 151.3, 151.4, 151.4 (C_Ar_), 166.7 (C=N), 172.4 (C=O) ppm. **Calcd. for** C_18_H_20_F_2_N_4_O, %: C 62.42; H 5.82; N 16.18; Found, %: C 62.69; H 6.06; N 16.13. **HRMS** *m/z* calcd. for C_18_H_20_F_2_N_4_O [M + H]^+^: 347.1605, Found: 347.2181.

#### 4.1.19. 3-[(2,4-Difluorophenyl)Amino]-N’-(4-Nitrobenzylidene)-Propanehydrazide (**7j**)

Yellow solid, yield 0.61 g, 92%, m. p. 174–176 °C (from 2-propanol). **FT-IR**: ν 3377, 3081 (2x NH), 1667 (C=O), 1518 (C=N) cm^−1^. **^1^H NMR** (400 MHz, DMSO-*d*_6_) δ (Z/E 60/40): 2.55 and 2.97 (2t, 2H, *J* = 6.9 Hz, NHCH_2_), 3.34–3.44 (m, 2H, CH_2_CO), 5.35 (s, 1H, NHCH_2_), 6.72–6.82 (m, 1H, H_Ar_), 6.89 (t, 1H, *J* = 8.5 Hz, H_Ar_), 7.07 (q, 1H, *J* = 9.9 Hz, H_Ar_), 7.38–7.98 (m, 2H, H_Ar_), 8.08 and 8.29 (2s, 1H, NHNCH) overlaps with 8.21–8.28 (m, 2H, H_Ar_), 11.66 and 11.72 (2s, 1H, CONH) ppm. **^13^C NMR** (101 MHz, DMSO-*d*_6_) δ 31.7 (NHCH_2_), 33.9 (CH_2_CO), 103.2, 103.4, 103.7, 104.0, 110.7, 110.8, 110.9, 111.7, 124.0, 127.6, 127.9, 133.4, 133.5, 140.5, 140.6, 140.7, 143.6, 147.6, 147.8, 148.9, 149.0 (C_Ar_), 167.8 (C=N), 173.5 (C=O) ppm. **Calcd. for** C_16_H_14_F_2_N_4_O_3_, %: C 55.17; H 4.05; N 16.09; Found, %: C 55.29; H 4.24; N 15.93. **HRMS** *m/z* calcd. for C_16_H_14_F_2_N_4_O_3_ [M + H]^+^: 349.1034, Found: 349.1606.

#### 4.1.20. N’-(2-Chloro-5-Nitrobenzylidene)-3-[(2,4-Difluorophenyl)Amino]Propanehydrazide (**7k**)

Yellowish solid, yield 0.61 g, 87%, m. p. 184–186 °C (from 2-propanol). **FT-IR**: ν 3429, 3084 (2x NH), 1675 (C=O), 1513 (C=N) cm^−1^. **^1^H NMR** (400 MHz, DMSO-*d*_6_) δ (Z/E 60/40): 2.55 and 2.98 (2t, 2H, *J* = 6.8 Hz, NHCH_2_), 3.35–3.45 (m, 2H, CH_2_CO), 5.33–5.41 (m, 1H, NHCH_2_), 6.73–6.93 (m, 2H, H_Ar_), 6.96–7.12 (m, 1H, H_Ar_), 7.78–7.85 (m, 1H, H_Ar_), 8.17–8.24 (m, 1H, H_Ar_), 8.38 and 8.56 (2s, 1H, NHNCH) overlaps with 8.61–8.65 (m, 1H, H_Ar_), 11.72 and 11.87 (2s, 1H, CONH) ppm. **^13^C NMR** (101 MHz, DMSO-*d*_6_) δ 31.6 (NHCH_2_), 34.0 (CH_2_CO), 103.1, 103.2, 103.4, 103.6, 110.6, 110.9, 111.9, 120.8, 121.0, 124.9, 125.1, 131.6, 132.9, 137.2, 138.8, 139.0, 140.0, 146.7, 148.7, 151.7, 154.0 (C_Ar_), 167.8 (C=N), 173.5 (C=O). **Calcd. for** C_16_H_13_ClF_2_N_4_O_3_, %: C 50.21; H 3.42; N 14.64; Found, %: C 49.96; H 3.42; N 14.48. **HRMS** *m/z* calcd. for C_16_H_13_ClF_2_N_4_O_3_ [M + H]^+^: 383.0644, Found: 383.1075.

#### 4.1.21. 3-[(2,4-Difluorophenyl)Amino]-N’-(Naphthalen-1-Ylmethylene)Propanehydrazide (**8**)

Light brown solid, yield 0.45 g, 67%, m. p. 158–160 °C (from 2-propanol). **FT-IR**: ν 3383, 3077 (2x NH), 1662 (C=O), 1516 (C=N) cm^−1^. **^1^H NMR** (400 MHz, DMSO-*d*_6_) δ (Z/E 60/40): 2.57 and 3.01 (2t, 2H, *J* = 7.0 Hz, NHCH_2_), 3.37–3.47 (m, 2H, CH_2_CO), 5.36–5.44 (m, 1H, NHCH_2_), 6.75–6.94 (m, 2H, H_Ar_), 7.02–7.14 (m, 1H, H_Ar_), 7.53–7.68 (m, 3H, H_Ar_), 7.87 (d, 1H, *J* = 7.2 Hz, H_Ar_), 8.01 (d, 2H, *J* = 7.7 Hz, H_Ar_), 8.54 and 8.85 (2d, 1H, *J* = 8.4 Hz, H_Ar_), 8.70 and 8.77 (2s, 1H, NHNCH), 11.43 and 11.53 (2s, 1H, CONH) ppm. **^13^C NMR** (101 MHz, DMSO-*d*_6_) δ 32.0 (NHCH_2_), 34.0 (CH_2_CO), 103.2, 103.4, 103.5, 103.6, 103.7, 110.7, 111.9, 111.9, 125.6, 126.3, 126.6, 127.3, 128.9, 129.5, 133.5, 142.4, 146.1 (C_Ar_), 167.3 (C=N), 173.0 (C=O) ppm. **Calcd. for** C_20_H_17_F_2_N_3_O, %: C 67.98; H 4.85; N 11.89; Found, %: C 67.81; H 5.02; N 11.96. **HRMS** *m/z* calcd. for C_20_H_17_F_2_N_3_O [M + H]^+^: 354.1340, Found: 354.1913.

#### 4.1.22. 3-[(2,4-Difluorophenyl)Amino]-N’-(Naphthalen-2-Ylmethylene)Propanehydrazide (**9**)

White solid, yield 0.42 g, 63%, m. p. 188–190 °C (from 2-propanol). **FT-IR**: ν 3352, 3059 (2x NH), 1659 (C=O), 1517 (C=N) cm^−1^. **^1^H NMR** (400 MHz, DMSO-*d*_6_) δ (Z/E 60/40): 2.55 and 3.00 (2t, 2H, *J* = 6.8 Hz, NHCH_2_), 3.25–3.48 (m, 2H, CH_2_CO), 5.38 (s, 1H, NHCH_2_), 6.74–6.83 (m, 1H, H_Ar_), 6.84–6.94 (m, 1H, H_Ar_), 7.02–7.13 (m, 1H, H_Ar_), 7.51–7.60 (m, 2H, H_Ar_), 7.86–8.01 (m, 4H, H_Ar_), 8.04 and 8.11 (2s, 1H, H_Ar_), 8.17 and 8.32 (2s, 1H, NHNCH), 11.45 (s, 1H, CONH) ppm. **^13^C NMR** (101 MHz, DMSO-*d*_6_) δ 31.8 (NHCH_2_), 334.0 (CH_2_CO), 103.2, 103.4, 103.5, 103.7, 110.7, 110.8, 110.9, 122.3, 126.8, 127.0, 127.8, 128.3, 128.5, 132.9, 143.1, 146.1, 148.9, 149.0, 151.3, 151.4, 154.0, 154.1, 154.1 (C_Ar_), 167.4 (C=N), 173.1 (C=O) ppm. **Calcd. for** C_20_H_17_F_2_N_3_O, %: C 67.98; H 4.85; N 11.89; Found, %: C 67.73; H 4.88; N 11.76. **HRMS** *m/z* calcd. for C_20_H_17_F_2_N_3_O [M + H]^+^: 354.1340, Found: 354.1903.

**General method of preparation of compounds 10–12**

The corresponding propanehydrazide (**7a**, **7d**, or **7f**, 0.9 mmol) was dissolved in dimethylformamide (3 mL). Potassium hydroxide (0.15 g, 2.7 mmol) and potassium carbonate (0.37 g, 2.7 mmol) were added, and the reaction mixture was stirred at 30 °C for 15 min. Then ethyl iodide (0.26 mL, 3.2 mmol) was added dropwise, and the reaction was stirred at the room temperature for 1 h. Later inorganic bases were separated by filtration, ethyl iodide excess was distilled under reduced pressure. The residue was diluted with distilled water (5 mL) while cooling. The obtained solids were filtered, washed with hot hexane (3 mL), dried and purified by recrystallization from 1,4-dioxane and water mixture (2:1) (10 mL). A Bunsen flask and a porcelain funnel with filter paper were used for filtration.

#### 4.1.23. N’-Benzylidene-3-[(2,4-Difluorophenyl)Amino]-N-Ethylpropanehydrazide (**10**)

Light brown solid, yield 0.14 g, 47%, m. p. 77–79 °C (from dioxane:water (2:1)). **FT-IR**: ν 3069 (NH), 1654 (C=O), 1519 (C=N) cm^−1^. **^1^H NMR** (400 MHz, DMSO-*d*_6_) δ: 1.07 (t, 3H, *J* = 6.8 Hz, CH_2_CH_3_), 3.08 (t, 2H, *J* = 6.9 Hz, NHCH_2_), 3.26–3.46 (m, 2H, CH_2_CO), 4.01 (q, 2H, *J* = 6.7 Hz, CH_2_CH_3_), 5.33 (s, 1H, NHCH_2_), 6.75 (dd, 1H, *J* = 15.0, 9.2 Hz, H_Ar_), 6.85 (t, 1H, *J* = 8.4 Hz, H_Ar_), 7.05 (t, 1H, *J* = 10.4 Hz, H_Ar_), 7.35–7.49 (m, 3H, H_Ar_), 7.74 (d, 2H, *J* = 7.3 Hz, H_Ar_), 8.02 (s, 1H, NN=CH) ppm. **^13^C NMR** (101 MHz, DMSO-*d*_6_) δ 11.0 (CH_2_CH_3_), 32.7 (NHCH_2_), 34.6 (CH_2_CO), 39.4 (CH_2_CH_3_), 103.2, 103.4, 103.7, 110.7, 110.9, 111.8, 126.9, 128.7, 129.5, 133.5, 133.6, 133.6, 135.0, 139.6, 148.9, 149.0, 151.3, 151.4, 151.6, 151.7, 153.9, 154.0 (C_Ar_), 169.8 (C=N), 172.2 (C=O) ppm. **Calcd. for** C_18_H_19_F_2_N_3_O, %: C 65.24; H 5.78; N 12.68; Found, %: C 65.05; H 5.70; N 12.50. **HRMS** *m/z* calcd. for C_18_H_19_F_2_N_3_O [M + H]^+^: 332.1496, Found: 332.2122.

#### 4.1.24. N’-(4-Chlorobenzylidene)-3-[(2,4-Difluorophenyl)Amino]-N-Ethylpropanehydrazide (**11**)

Yellowish solid, yield 0.15 g, 46%, m. p. 85–87 °C (from dioxane:water (2:1)). **FT-IR**: ν 3074 (NH), 1664 (C=O), 1508 (C=N) cm^−1^. **^1^H NMR** (400 MHz, DMSO-*d*_6_) δ: 1.06 (t, 3H, *J* = 6.9 Hz, CH_2_CH_3_), 3.07 (t, 2H, *J* = 6.8 Hz, NHCH_2_), 3.36 (s, 2H, CH_2_CO), 3.99 (q, 2H, *J* = 6.7 Hz, CH_2_CH_3_), 5.31 (s, 1H, NHCH_2_), 6.68–6.79 (m, 1H, H_Ar_), 6.80–6.90 (m, 1H, H_Ar_), 6.99–7.10 (m, 1H, H_Ar_), 7.49 (d, 2H, *J* = 8.2 Hz, H_Ar_), 7.75 (d, 2H, *J* = 8.2 Hz, H_Ar_), 8.02 (s, 1H, NN=CH) ppm. **^13^C NMR** (101 MHz, DMSO-*d*_6_) δ 10.9 (CH_2_CH_3_), 32.6 (NHCH_2_), 34.7 (CH_2_CO), 39.4 (CH_2_CH_3_), 103.2, 103.4, 103.4, 103.7, 110.6, 110.9, 111.8, 128.5, 128.8, 133.5, 133.6, 133.6, 133.9, 138.4, 148.9, 149.0, 151.3, 151.4, 151.6, 151.7, 153.9, 154.0 (C_Ar_), 169.0 (C=N), 172.3 (C=O) ppm. **Calcd. for** C_18_H_18_ClF_2_N_3_O, %: C 56.10; H 4.96; N 11.49; Found, %: C 56.27; H 5.16; N 11.29. **HRMS** *m/z* calcd. for C_18_H_18_ClF_2_N_3_O [M + H]^+^: 366.1106, Found: 366.1618.

#### 4.1.25. 3-[(2,4-Difluorophenyl)Amino]-N-Ethyl-N’-(4-Methylbenzylidene)Propanehydrazide (**12**)

Light grey solid, yield 0.15 g, 47%, m. p. 85–87 °C (from dioxane:water (2:1)). **FT-IR**: ν 3071 (NH), 1656 (C=O), 1508 (C=N) cm^−1^. **^1^H NMR** (400 MHz, DMSO-*d*_6_) δ: 1.06 (t, 3H, *J* = 6.8 Hz, CH_2_CH_3_), 2.33 (s, 3H, CH_3_), 3.07 (t, 2H, *J* = 6.9 Hz, NHCH_2_), 3.26–3.45 (m, 2H, CH_2_CO), 3.99 (q, 2H, *J* = 6.6 Hz, CH_2_CH_3_), 5.32 (s, 1H, NHCH_2_), 6.74 (dd, 1H, *J* = 15.0, 9.2 Hz, H_Ar_), 6.85 (t, 1H, *J* = 8.5 Hz, H_Ar_), 7.05 (t, 1H, *J* = 8.6 Hz, H_Ar_), 7.24 (d, 2H, *J* = 7.7 Hz, H_Ar_), 7.63 (d, 2H, *J* = 7.7 Hz, H_Ar_), 7.97 (s, 1H, NN=CH) ppm. **^13^C NMR** (101 MHz, DMSO-*d*_6_) δ 11.0 (CH_2_CH_3_), 21.0 (CH_3_), 32.7 (NHCH_2_), 34.5 (CH_2_CO), 39.4 (CH_2_CH_3_), 103.2, 103.4, 103.5, 103.7, 110.6, 110.7, 110.8, 110.9, 126.9, 129.4, 132.3, 133.6, 133.7, 133.7, 139.3, 139.7, 148.9, 149.0, 151.3, 151.4, 151.6, 151.7, 154.0, 154.1 (C_Ar_), 168.6 (C=N), 172.1 (C=O) ppm. **Calcd. for** C_19_H_21_F_2_N_3_O, %: C 66.07; H 6.17; N 12.17; Found, %: C 65.92; H 6.11; N 11.96. **HRMS** *m/z* calcd. for C_19_H_21_F_2_N_3_O [M + H]^+^: 346.1653, Found: 346.2230.

**General method of the preparation of hydrazones 13–15**

Hydrazide **4** (0.40 g, 1.9 mmol) was dissolved in 15 mL of acetone (to obtain product **13**) or 15 mL of ethyl methyl ketone (to obtain product **14**). Compound **15** was synthesized by dissolving the same amounts hydrazide **4** in 2-propanol (15 mL), to which 4-aminoacetophenone (0.27 g, 2.0 mmol) was added. Each reaction was catalyzed with 4 drops of acetic acid, performed for 3 h, at the boiling temperature of the mixture. The obtained solids were filtered off, washed with hot hexane (3 mL), dried and later purified by recrystallization from a mixture of 2-propanol and water (2:1) (10 mL). A Bunsen flask and a porcelain funnel with filter paper were used for filtration.

#### 4.1.26. 3-[(2,4-Difluorophenyl)Amino]-N’-(Propan-2-Ylidene)Propanehydrazide (**13**)

Light purple solid, yield 0.35 g, 72%, m. p. 120–122 °C (from 2-propanol:water (2:1)). **FT-IR**: ν 3357, 3091 (2x NH), 1675 (C=O), 1526 (C=N) cm^−1^. **^1^H NMR** (400 MHz, DMSO-*d*_6_) δ: 1.83 and 1.90 (2s, 6H, NCCH_3_), 2.77 (t, 1H, *J* = 7.0 Hz, NHCH_2_), 3.24–3.34 (m, 2H, CH_2_CO), 3.37–3.45 (m, 1H, NHCH_2_), 5.21–5.34 (m, 1H, NHCH_2_), 6.70–6.81 (m, 1H, H_Ar_), 6.82–6.92 (m, 1H, H_Ar_), 7.00–7.12 (m, 1H, H_Ar_), 10.01 and 10.07 (2s, 1H, CONH) ppm. **^13^C NMR** (101 MHz, DMSO-*d*_6_) δ 17.1, 17.5, 25.0, 25.2 (2x CH_3_), 32.3 (NHCH_2_), 33.6 (CH_2_CO), 103.2, 103.4, 103.4, 103.7, 110.7, 110.9, 111.8, 133.5, 133.6, 148.9, 149.0, 150.6, 151.3, 151.4, 151.6, 154.0, 155.1 (C_Ar_), 167.3 (C=N), 173.1 (C=O) ppm. **Calcd. for** C_12_H_14_F_2_N_3_O, %: C 56.46; H 5.92; N 16.46; Found, %: 56.30; H 6.10; N 16.47. **HRMS** *m/z* calcd. for C_12_H_15_F_2_N_3_O [M + H]^+^: 256.1183, Found: 256.1845.

#### 4.1.27. N’-(Butan-2-Ylidene)-3-[(2,4-Difluorophenyl)Amino]Propanehydrazide (**14**)

Light purple solid, yield 0.36 g, 70%, m. p. 82–84 °C (from 2-propanol:water (2:1)). **FT-IR**: ν 3387, 3093 (2x NH), 1673 (C=O), 1526 (C=N) cm^−1^. **^1^H NMR** (400 MHz, DMSO-*d*_6_) δ (Z/E 60/40): 1.00 (t, 3H, *J* = 7.4 Hz, CH_2_CH_3_), 1.82 and 1.88 (2s, 3H, NCCH_3_), 2.14–2.30 (m, 2H, CH_2_CH_3_), 2.44–2.55 (m, 1H, NHCH_2_ overlaps with DMSO-*d*_6_), 2.80 (t, 1H, *J* = 6.9 Hz, NHCH_2_), 3.25–3.35 (m, 2H, CH_2_CO), 5.22–5.34 (m, 1H, NHCH_2_), 6.68–6.79 (m, 1H, H_Ar_), 6.80–6.89 (m, 1H, H_Ar_), 7.00–7.13 (m, 1H, H_Ar_), 9.98 and 10.08 (2s, 1H, CONH) ppm. **^13^C NMR** (101 MHz, DMSO-*d*_6_) δ 9.8, 10.6, 10.8 (NCCH_3_), 15.7, 16.0 (CH_2_CH_3_), 22.5, 22.9 (CH_2_CH_3_), 31.5, 31.6 (NHCH_2_), 32.2, 33.7 (CH_2_CO), 103.1, 103.4, 103.6, 110.6, 110.8, 111.8, 111.8, 133.5, 133.6, 148.9, 149.0, 151.3, 151.4, 154.0, 154.1, 158.5 (C_Ar_), 167.3 (C=N), 173.3 (C=O) ppm. **Calcd. for** C_13_H_17_F_2_N_3_O, %: C 57.98; H 6.36; N 15.60; Found, %: C 57.77; H 6.58; N 15.42. **HRMS** *m/z* calcd. for C_13_H_17_F_2_N_4_O [M + H]^+^: 270.1340, Found: 270.2015.

#### 4.1.28. N’-[1-(4-Aminophenyl)Ethylidene]-3-[(2,4-Difluorophenyl)Amino]Propanehydrazide (**15**)

Light brown solid, yield 0.46 g, 73%, m. p. 181–183 °C (from 2-propanol:water (2:1)). **FT-IR**: ν 3464, 3335, 3231, 3040 (NH_2_, 2x NH), 1654 (C=O), 1513 (C=N) cm^−1^. **^1^H NMR** (400 MHz, DMSO-*d*_6_) δ (Z/E 60/40): 2.13 (s, 3H, NCCH_3_), 2.58 and 2.93 (2t, 2H, *J* = 6.6, 7.0 Hz, NHCH_2_), 3.29–3.42 (m, 2H, CH_2_CO), 5.28–5.38 (m, 1H, NHCH_2_), 5.39–5.49 (m, 2H, NH_2_), 6.55 (d, 2H, *J* = 8.2 Hz, H_Ar_), 6.71–6.93 (m, 2H, H_Ar_), 7.00–7.13 (m, 1H, H_Ar_), 7.43–7.53 (m, 2H, H_Ar_), 10.15 and 10.26 (2s, 1H, CONH) ppm. **^13^C NMR** (101 MHz, DMSO-*d*_6_) δ 13.3, 13.7, 25.5 (NCCH_3_), 32.3 (NHCH_2_), 33.8 (CH_2_CO), 103.2, 103.4, 103.4, 103.6, 110.6, 110.8, 113.1, 113.2, 125.3, 125.4, 127.2, 127.5, 133.5, 133.5, 133.6, 133.6, 148.9, 149.9, 150.1, 151.4, 151.7, 152.6, 154.0 (C_Ar_), 167.3 (C=N), 173.4 (C=O) ppm. **Calcd. for** C_17_H_18_F_2_N_4_O, %: C 61.44; H 5.46; N 16.86; Found, %: C 61.43; H 5.50; N 16.67. **HRMS** *m/z* calcd. for C_17_H_18_F_2_N_4_O [M + H]^+^: 333.1449, Found: 333.2054.

**General method of the preparation of compounds 16 and 17**

In order to obtain compound **16**, propanehydrazide **4** (0.40 g, 1.9 mmol) was dissolved in 15 mL of 2-propanol, later 2,4-pentanedione (0.29 g, 2.9 mmol) was added dropwise, successively hydrochloric acid (12 M, 1 drop) was used added. In order to synthesize compound **17**, 2,5-hexanedione (0.39 g, 3.4 mmol) and 2 drops of acetic acid were added dropwise to the prepared hydrazide **4** solution. In each case, the reaction was carried out under reflux for 2 h and after cooling the reaction solution, the obtained solids were filtered, dried and recrystallized from a mixture of 2-propanol and water (1:2) (10 mL). A Bunsen flask and a porcelain funnel with filter paper were used for filtration. This synthesis method was adapted based on conditions described in our previously published studies [35,42,45].

#### 4.1.29. 3-[(2,4-Difluorophenyl)Amino]-1-(3,5-Dimethyl-1H-Pyrazol-1-yl)Propan-1-One (**16**)

Light brown solid, yield 0.39 g, 74%, m. p. 66–68 °C (from 2-propanol:water (1:2)). **FT-IR**: ν 3408, 3094 (2x NH), 1711 (C=O), 1522 (C=N) cm^−1^. **^1^H NMR** (400 MHz, DMSO-*d*_6_) δ: 2.21 and 2.54 (2s, 6H, 2x CH_3_ overlaps with DMSO-*d*_6_), 3.29–3.40 (m, 2H, NHCH_2_), 3.41–3.51 (m, 2H, CH_2_CO), 5.47 (s, 1H, NHCH_2_), 6.21 (s, 1H, NCCH), 6.78–6.86 (m, 1H, H_Ar_), 6.87–6.95 (m, 1H, H_Ar_), 7.05–7.16 (m, 1H, H_Ar_) ppm. **^13^C NMR** (101 MHz, DMSO-*d*_6_) δ 13.5, 14.1 (2x CH_3_), 34.7 (NHCH_2_), 38.5 (CH_2_CO), 103.2, 103.4, 103.4, 103.7, 110.6, 110.8, 110.9, 111.2, 111.8, 111.9, 133.3, 133.4, 133.5, 143.2, 148.9, 149.0, 151.3, 151.4, 151.7, 151.8, 154.0, 154.1 (C_Ar_), 172.0 (C=O) ppm. **Calcd. for** C_14_H_15_F_2_N_3_O, %: C 60.21; H 5.41; N 15.05; Found, %: C 60.08; H 5.46; N 14.86. **HRMS** *m/z* calcd. for C_14_H_15_F_2_N_3_O [M + H]^+^: 280.1183, Found: 280.1851.

#### 4.1.30. 3-[(2,4-Difluorophenyl)Amino]-N-(2,5-Dimethyl-1H-Pyrrol-1-yl)Propanamide (**17**)

Light grey solid, yield 0.47 g, 89%, m. p. 150–152 °C (from 2-propanol:water (1:2)). **FT-IR**: ν 3345, 3081 (2x NH), 1662 (C=O) cm^−1^. **^1^H NMR** (400 MHz, DMSO-*d*_6_) δ: 1.95 (s, 6H, 2x CH_3_), 2.58 (t, 2H, *J* = 6.7 Hz, NHCH_2_), 3.31–3.45 (m, 2H, CH_2_CO), 5.31 (s, 1H, NHCH_2_), 5.62 and 5.70 (2s, 2H, 2x NCCH), 6.73–6.85 (m, 1H, H_Ar_), 6.86–6.94 (m, 1H, H_Ar_), 7.02–7.14 (m, 1H, H_Ar_), 10.63 (s, 1H, CONH) ppm. **^13^C NMR** (101 MHz, DMSO-*d*_6_) δ 10.9, 11.0 (2x CH_3_), 33.0 (NHCH_2_), 39.4 (CH_2_CO, overlaps with DMSO-*d*_6_), 102.9, 103.2, 103.4, 103.7, 103.9, 110.7, 110.9, 112.0, 126.7, 127.1, 133.2, 133.3, 148.9, 149.0, 151.3, 151.4, 151.7, 151.8, 154.1, 154.2 (C_Ar_), 170.4 (C=O) ppm. **Calcd. for** C_15_H_17_F_2_N_3_O, %: C 61.42; H 5.84; N 14.33; Found, %: C 61.21; H 6.01; N 14.13. **HRMS** *m/z* calcd. for C_15_H_17_F_2_N_3_O [M + H]^+^: 294.1340, Found: 294.2022.

**General method of the preparation of compounds 18 and 19**

Hydrazide **4** (0.40 g, 1.9 mmol) was dissolved in methanol (10 mL), later the reagent was added dropwise (in the case of product **18**—0.37 g, 3.1 mmol of phenylisocyanate, in the case of product **19**—0.28 g, 2.1 mmol of phenylisothiocyanate). The reactions were carried out at the boiling temperature of the mixture for 5 h (to synthesize product **18**) or 1 h (to synthesize product **19**), respectively. After the reaction mixture was cooled down, the obtained solids were filtered off, dried and purified by recrystallizing from 1,4-dioxane (10 mL). A Bunsen flask and a porcelain funnel with filter paper were used for filtration.

#### 4.1.31. 2-{3-[(2,4-Difluorophenyl)Amino]Propanoyl}-N-Phenylhydrazine-1-Carboxamide (**18**)

Brown solid, yield 0.52 g, 82%, m. p. 167–169 °C (from 1,4-dioxane). **FT-IR**: ν 3372, 3343, 3264, 3200 (4x NH), 1704, 1662 (C=O) cm^−1^. **^1^H NMR** (400 MHz, DMSO-*d*_6_) δ: 2.40–2.47 (m, 2H, NHCH_2_ overlaps with DMSO-*d*_6_), 3.24–3.28 (m, 2H, CH_2_CO), 5.24–5.34 (m, 1H, NHCH_2_), 6.66–6.81 (m, 1H, H_Ar_), 6.83–6.91 (m, 1H, H_Ar_), 6.92–7.01 (m, 1H, H_Ar_), 7.02–7.13 (m, 1H, H_Ar_), 7.19–7.31 (m, 2H, H_Ar_), 7.37–7.52 (m, 2H, H_Ar_), 8.05, 8.71, 9.75 (3s, 3H, 3x NH) ppm. **^13^C NMR** (101 MHz, DMSO-*d*_6_) δ 32.9 (CH_2_CO), 51.6 (NHCH_2_), 103.2, 103.4, 103.7, 110.7, 110.9, 111.9, 118.5, 121.9, 128.7, 128.8, 133.4, 133.5, 139.6, 148.9, 149.0, 151.3, 151.4, 154.0, 154.1, 154.2, 155.4, 155.4 (C_Ar_), 170.9 (2x C=O) ppm. **Calcd. for** C_16_H_16_F_2_N_4_O_2_, %: C 57.48; H 4.82; N 16.76; Found, %: C 57.50; H 5.07; N 16.62. **HRMS** *m/z* calcd. for C_16_H_16_F_2_N_4_O_2_ [M + H]^+^: 335.1241, Found: 335.1840.

#### 4.1.32. 2-{3-[(2,4-Difluorophenyl)Amino]Propanoyl}-N-Phenylhydrazine-1-Carbothioamide (**19**)

Light purple solid, yield 0.57 g, 85%, m. p. 178–180 °C (from 1,4-dioxane). **FT-IR**: ν 3363, 3287, 3133, 3063 (4x NH), 1675 (C=O), 1208 (C=S) cm^−1^. **^1^H NMR** (400 MHz, DMSO-*d*_6_) δ: 2.45–2.57 (m, 2H, NHCH_2_ overlaps with DMSO-*d*_6_), 3.26–3.39 (m, 2H, CH_2_CO), 5.31 (s, 1H, NHCH_2_), 6.68–6.80 (m, 1H, H_Ar_), 6.82–6.91 (m, 1H, H_Ar_), 7.02–7.12 (m, 1H, H_Ar_), 7.13–7.21 (m, 2H, H_Ar_), 7.32 (t, 2H, *J* = 7.6 Hz, H_Ar_), 7.37–7.49 (m, 2H, H_Ar_), 9.56, 9.98 (3s, 3H, 3x NH) ppm. **^13^C NMR** (101 MHz, DMSO-*d*_6_) δ 33.0 (CH_2_CO), 48.8 (NHCH_2_), 103.1, 103.4, 103.4, 103.7, 110.9, 112.0, 125.2, 128.2, 133.4, 133.4, 133.5, 133.6, 139.1, 148.9, 149.0, 151.3, 151.4, 151.7, 151.8, 154.0, 154.2 (C_Ar_), 170.8 (C=O), 180.9 (C=S) ppm. **Calcd. for** C_16_H_16_F_2_N_4_OS, %: 54.85; H 4.60; N 15.99; Found, %: C 55.02; H 4.84; N 15.71. **HRMS** *m/z* calcd. for C_16_H_16_F_2_N_4_OS [M + H]^+^: 351.1013, Found: 351.1575.

#### 4.1.33. 5-{2-[(2,4-Difluorophenyl)Amino]Ethyl}-4-Phenyl-2,4-Dihydro-3H-1,2,4-Triazol-3-One (**20**)

The starting material—carboxamide **18** (0.41 g, 1.2 mmol)—was dissolved in aqueous 4% sodium hydroxide solution (11 mL). The cyclization reaction was carried out at the boiling temperature of the mixture for 2 h. After cooling, the mixture was neutralized using acetic acid in ice-water bath to pH 6, the precipitated crystals were filtered, dried and purified by recrystallization from 1,4-dioxane (10 mL). A Bunsen flask and a porcelain funnel with filter paper were used for filtration.

Light brown solid, yield 0.22 g, 58%, m. p. 151–153 °C (from 1,4-dioxane). **FT-IR**: ν 3327, 3064 (2x NH), 1689 (C=O), 1527 (C=N) cm^−1^. **^1^H NMR** (400 MHz, DMSO-*d*_6_) δ: 2.64 (t, 2H, *J* = 6.6 Hz, NHCH_2_), 3.12–3.29 (m, 2H, CH_2_CO), 5.33–5.45 (m, 1H, NHCH_2_), 6.34 (dd, 1H, *J* = 14.4, 8.9 Hz, H_Ar_), 6.67–6.81 (m, 1H, H_Ar_), 7.03 (t, 1H, *J* = 8.8 Hz, H_Ar_), 7.32–7.60 (m, 5H, H_Ar_), 11.74 (s, 1H, C=NNH) ppm. **^13^C NMR** (101 MHz, DMSO-*d*_6_) δ 25.5 (CH_2_C=N), 34.1 (NHCH_2_), 103.3, 103.5, 103.8, 110.6, 110.8, 110.8, 111.4, 127.7, 128.8, 129.5, 133.0, 133.1, 145.3, 149.0, 151.3, 151.4, 151.6, 151.8, 154.0, 154.1, 154.5 (C_Ar_), 170.1 (C=O) ppm. **Calcd. for** C_16_H_14_F_2_N_4_O, %: C 60.76; H 4.46; N 17.71; Found, %: C 60.58; H 4.61; N 17.59. **HRMS** *m/z* calcd. for C_16_H_14_F_2_N_4_O [M + H]^+^: 317.1136, Found: 317.1786.

#### 4.1.34. 5-{2-[(2,4-Difluorophenyl)Amino]Ethyl}-4-Phenyl-2,4-Dihydro-3H-1,2,4-Triazole-3-Thione (**21**)

The starting material **19** (0.84 g, 2.4 mmol) was dissolved in aqueous 4% sodium hydroxide solution (14 mL). After stirring at reflux for 1.5 h, the reaction mixture was cooled, later neutralized with acetic acid in ice-water bath to pH 6. The obtained solids were filtered off, dried, recrystallized from 1,4-dioxane (10 mL). A Bunsen flask and a porcelain funnel with filter paper were used for filtration.

White solid, yield 0.75 g, 94%, m. p. 148–150 °C (from 1,4-dioxane). **FT-IR**: ν 3281, 3062 (2x NH), 1521 (C=N), 1268 (C=S) cm^−1^. **^1^H NMR** (400 MHz, DMSO-*d*_6_) δ: 2.67 (t, 2H, *J* = 7.0 Hz, NHCH_2_) 3.16–3.30 (m, 2H, CH_2_CO), 5.40–5.52 (m, 1H, NHCH_2_), 6.25–6.37 (m, 1H, H_Ar_), 6.73 (t, 1H, *J* = 8.4 Hz, H_Ar_), 7.44 and 7,55 (2d, 5H, *J* = 7.4, 6.4 Hz, H_Ar_), 7.38–7.84 (m, 2H, H_Ar_), 7.49–7.61 (m, 2H, H_Ar_), 11.11 (s, 1H, C=NNH) ppm. **^13^C NMR** (101 MHz, DMSO-*d*_6_) δ 25.0 (CH_2_C=N), 39.7 (NHCH_2_ overlaps with DMSO-*d*_6_), 103.3, 103.5, 103.5, 103.8, 110.6, 110.8, 111.3, 128.5, 129.5, 132.8, 132.8, 133.8, 148.8, 149.0, 150.5, 151.2, 151.6, 151.7, 154.0, 154.1 (C_Ar_), 167.7 (C=N), 179.2 (C=S) ppm. **Calcd. for** C_16_H_14_F_2_N_4_S, %: C 57.82; H 4.25; N 16.86; Found, %: C 57.60; H 4.18; N 16.67. **HRMS** *m/z* calcd. for C_16_H_14_F_2_N_4_S [M + H]^+^: 333.0907, Found: 333.1514.

### 4.2. Cell Lines and Culture Conditions

A549 human non-small-cell lung carcinoma cells (ATCC CCL-185) and HEK293 cells were obtained from the American Type Culture Collection (Rockville, MD, USA). Caco-2 and HEK293 cell lines were generously supplied by the Iliev laboratory at the Jill Roberts Institute for Inflammatory Bowel Disease, Weill Cornell Medicine, Cornell University (New York, NY, USA). All cell lines were maintained in Dulbecco’s Modified Eagle Medium/Nutrient Mixture F-12 (DMEM/F-12; Gibco, Waltham, MA, USA) supplemented with 10% fetal bovine serum (FBS; Gibco) and antibiotics (100 U/mL penicillin and 100 µg/mL streptomycin; Gibco). Cultures were kept at 37 °C in a humidified incubator with 5% CO_2_. Media were replaced every 2–3 days, and cells were subcultured once they reached approximately 70–80% confluence [57,58].

### 4.3. MTT Antiproliferative Assay

The antiproliferative activity of the synthesized compounds was evaluated using an MTT-based viability assay. Cells were seeded into 96-well plates at a density of 1 × 10^4^ cells per well and allowed to adhere overnight under standard culture conditions (37 °C, 5% CO_2_). The test compounds and control agents were dissolved in DMSO to achieve 25–50 mg/mL stock solution. The following day, cells were exposed to test compounds at a final concentration of 100 µM, with each condition tested in triplicate in complete growth media with final concentration of 0.5% of DMSO. Cells without test or control agents but containing growth media with DMSO were referred to untreated control (UC). After 20 h of treatment, commercial MTT (Invitrogen, Carlsbad, CA, USA, # V13154) solution was added to each well and incubated for an additional 4 h to allow formation of formazan crystals. These crystals were dissolved in anhydrous DMSO, and absorbance was recorded at 570 nm using a microplate spectrophotometer. Cell viability was determined using the equation ([AE − AB]/[AC − AB]) × 100%, where AE corresponds to absorbance from treated wells, AC to untreated control wells, and AB to blank wells. Viability and IC_50_ analysis were conducted using GraphPad Prism or QuickCalcs (version 2.0) [35].

### 4.4. Molecular Modeling

#### 4.4.1. Receptor Preparation

The crystal structures of 17 selected proteins (Table 2) were retrieved from the Protein Data Bank [59]. These proteins, as (MAPK1, ERK2, MEK1, TPK), mesenchymal epithelial transition factor (MET), cyclooxygenase-2 (COX-2), kinases, tropomyosin receptor kinase A (TRKA), vascular endothelial growth factor receptor 2 (VEGFR-2), estrogen receptors (ERs), and among others, are overexpressed in lung and colorectal cancer cell lines [60,61,62,63,64,65,66,67,68,69,70].

#### 4.4.2. Ligand Preparation

The 3D structures of the candidate compounds **6b**, **7f**, **7g** and **9** were built using GaussView 5.0 and geometrically optimized using Avogadro (2.0 software). These structures were visually checked to correct some structural errors. The 3D structure of ligands was extracted from crystal 3RHK and 7JXH, whereas the structure of inhibitor drug (erlotinib) was taken PubChem database with compound CID: 176870. Erlotinib and crizotinib were used as reference inhibitors for in silico docking due to the availability of well-characterized crystallographic complexes.

#### 4.4.3. Docking of Protein-Ligand Interaction

The compounds were docked into proteins to identify its potential binding site. Both ligand and protein were prepared using AutoDock Tools version 1.5.7, as previously described [46]. Docking calculations were performed using AutoDock Vina 1.2.0. Finally, graphical analysis was performed using VMD (version 1.9.4a57 ) and Discovery Studio (version 2023) [71,72].

### 4.5. Clonogenic Survival Assay

A549 cells were seeded at a density of 5 × 10^2^ cells per well in 6-well plates and allowed to attach overnight. Cells were then treated with crizotinib (MedChemExpress) or lapatinib (MedChemExpress), compound **9**, or the indicated combinations for 12 h. Following treatment, the medium containing test compounds was removed, cells were gently washed with phosphate-buffered saline (PBS), and fresh drug-free complete medium was added. Cells were then incubated for 10 days to allow colony formation, with medium replaced every 4 days. At the end of the incubation period, colonies were fixed with methanol, and stained with 0.5% (*w*/*v*) crystal violet solution, rinsed thoroughly with distilled water, and air-dried. Stained colonies were imaged using a light transilluminator, and colonies consisting of ≥50 cells were manually counted. Data are expressed as the mean ± standard deviation (SD) from three independent experiments.

### 4.6. Statistical Analysis

The data are expressed as the mean ± SD from three independent experiments, unless otherwise stated. Statistical significance was determined using a one-way ANOVA test in GraphPad Prism software (version Prism 10). A *p* < 0.05 was considered statistically significant.

## 5. Conclusions

The present work establishes the 3-[(2,4-difluorophenyl)amino]propanoic acid framework as a promising chemotype for generating antiproliferative small molecules with activity across epithelial-derived cancer models. In particular, compounds **6b**, **7f**, **7g**, and **9**, bearing 5-nitro-2-furyl, 4-methylphenyl, 4-methoxyphenyl, and 2-naphthyl substituents, displayed the most promising activity in our antiproliferative assays. Evaluation in non-malignant HEK293 human kidney epithelial cells demonstrated measurable cytotoxicity, indicating that the intrinsic bioactivity of this scaffold is not entirely cancer-restricted and underscoring the importance of further structural refinement to enhance selectivity toward malignant phenotypes. While this observation underscores the intrinsic bioactivity of the scaffold, it also highlights the need for rational optimization to enhance cancer selectivity, refine physicochemical properties, and reduce off-target effects. Collectively, the structure–activity relationships identified in this study provide a foundation for refinement of 3-[(2,4-difluorophenyl)amino]propanoic acid scaffold. Future work to elucidate the molecular targets and signaling pathways affected by these compounds, complemented by in vivo pharmacological and toxicological characterization to validate 3-[(2,4-difluorophenyl)amino]propanoic acid scaffold as lead candidates for anticancer drug development. Importantly, this study expands the chemical space of fluorinated β-alanine-derived scaffolds in anticancer research and highlights the potential of rationally modified 2,4-difluorophenyl-containing frameworks as a basis for the development of next-generation antiproliferative agents.

## Figures and Tables

**Table 1:** Structures of the synthesized compounds and their yields.

Structure No.	Chemical Structure	Yield, %	Structure No.	Chemical Structure	Yield, %
**2**	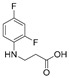	65	**7i**	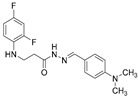	92
**3**	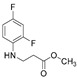	93	**7j**	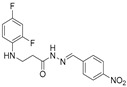	92
**4**	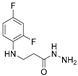	82	**7k**	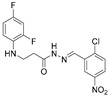	87
**5a**	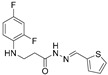	91	**8**	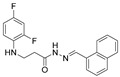	67
**5b**	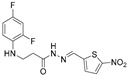	91	**9**	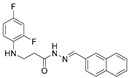	63
**5c**	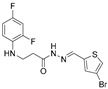	90	**10**	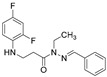	47
**6a**	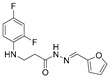	81	**11**	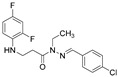	46
**6b**	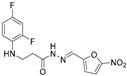	89	**12**	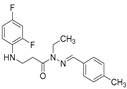	47
**6c**	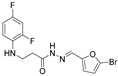	83	**13**	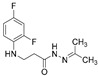	72
**7a**	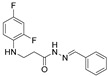	89	**14**	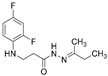	70
**7b**	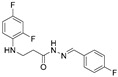	93	**15**	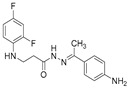	73
**7c**	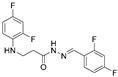	78	**16**	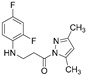	74
**7d**	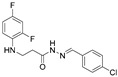	**86**	**17**	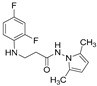	89
**7e**	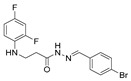	86	**18**	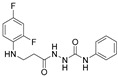	82
**7f**	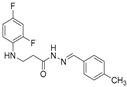	91	**19**	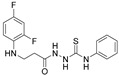	85
**7g**	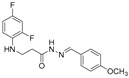	87	**20**	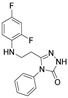	58
**7h**	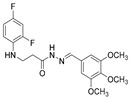	75	**21**	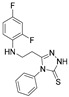	94

**Table 2:** Predicted binding free energy values (∆G_bin_ kcal/mol) of synthesized antiproliferative hybrids with selected proteins overexpressed in cancer.

Compounds	Target Proteins																
	DHFR	TXNRD1	COX-2	FGFR-2	VEGRF-2	NR3A1	EGFR	HER2	NR3A2	c-MET	ERK2	MEK1	TRKA	CK4	TPK	HDAC2	SIRT1
**6b**	−8.4	−9.8	−9.2	−8.0	−8.0	−7.9	−9.1	−9.3	−8.3	−9.1	−8.3	−8.8	−8.7	−7.8	−8.0	−6.9	−9.9
**7f**	−8.3	−9.0	−8.7	−8.3	−8.1	−7.8	−9.4	−9.5	−7.9	−9.4	−7.6	−8.9	−8.4	−7.2	−8.4	−6.9	−8.8
**7g**	−8.2	−8.9	−8.3	−8.1	−7.9	−7.6	−8.9	−9.5	−8.1	−9.0	−8.2	−9.1	−8.8	−7.5	−8.4	−6.7	−9.1
**9**	−9.4	−9.0	−9.5	−9.4	−9.0	−9.1	−10.4	−10.7	−9.2	−11.1	−8.9	−8.8	−10.0	−7.8	−10.0	−7.6	−9.7
**P avge.**	−8.57	−9.16	−8.91	−8.45	−8.27	−8.09	−9.44	**−9.76**	−8.35	**−9.65**	−8.26	−8.89	−8.98	−7.59	−8.70	−7.04	−9.40

Proteins with their respective (PDB) entries: DHFR: Dihydrofolate reductase (1DLS); TXNRD1: Thioredoxin Reductase 1 (2ZZ0); COX-2: Cyclooxygenase 2 (3LN2); FGFR-2: Fibroblast growth factor receptor 2 (1GJO); VEGRF-2: Vascular endothelial growth factor receptor 2 (3VHE); NR3A1: Estrogen receptor α (3ERT); EGFR: Epidermal growth factor receptor (5GTY); HER2: Epidermal growth factor receptor 2 (7JXH); NR3A2: Estrogen receptor beta (2QTU); c-MET: Mesenchymal-epithelial transition factor (3RHK); ERK2: Extra-cellular signal-regulated kinase 2 (2OJG); MEK1: MAPK/ERK kinase (4AN3); TRKA: tyrosine kinase receptor (6PL2); CK4: Cyclin-dependent kinase 4 (1G3N); TPK: Tyrosine-protein kinase (4EHZ); HDAC2: histone deacetylase 2 (3MAX); SIRT1: NAD-dependent sirtuin-1 deacetylase (4ISI). P avge.: Protein average, mean of the ∆G_bin_ values for the interactions of each protein with all the hybrids; the two proteins with the highest global compounds affinity are highlighted on red color.

**Table 3:** Binding site contacts of compound **9**, the reference ligands, and erlotinib within the c-MET and HER2 catalytic pockets.

Compound	ΔG*_bin_* (kcal/mol)	H Bonds and Hydrophobic Contacts in the Binding Site
		c-MET
**9**	−11.1	His1088, Phe1089, Val1092, Ala1108, Lys1110, Val1155, Leu1157, Asp1164, Asn1167, Arg1208, Met1211, Phe1223, Ala1226, **Arg1227**, Met1229, Tyr1230, **Asp1231**, Tyr1234, Tyr1235
**Ligand 1 ^[a]^**	−14.6	Ile1084, Gly1085, Phe1089, Val1092, Ala1108, Lys1110, Leu1140, Leu1157, **Pro1158**, Tyr1159, **Met1160**, Gly1163, Met1211, Phe1223, Ala1226, Arg1227
**Crizotinib ** **^[b]^**	−9.9	Ile1084, Phe1089, Val1092, Ala1108, Lys1110, Leu1157, Gly1163, Asp1164, **Asn1167**, Arg1208, Met1211, Phe1223, Ala1226, Arg1227, Asp1231, Tyr1234
		**HER2**
**9**	−10.7	Leu726, Val734, Ala751, Ile767, Glu770, Ala771, Met774, Ser783, Arg784, Leu785, Leu796, **Thr798**, Leu800, Met801, Gly804, Leu852, **Thr862**, Asp863, Phe864
**Ligand 2 ^[a]^**	−14.6	Leu726, Val734, Ala751, Ile752, Lys753, Met774, **Ser783**, Leu785, Leu796, Thr798, Gln799, Leu800, Met801, Glu804, **Cys805**, Asp808, Leu852, Thr862, Asp863, Phe864
**Erlotinib ** **^[b]^**	−8.1	Leu726, Gly727, Ser728, Val734, Ala751, Lys753, Met774, Ser783, Leu785, Leu796, Thr798, Gln799, Leu800, Met801, Gly804, Cys805, Leu852, **Thr862**, Asp863, Phe864

^[a]^ Ligands 1 and 2 correspond to 1-[(3*R*,4*R*)-4-(1*H*-indol-3-yl)-2,5-dioxopyrrolidin-3-yl]pyrrolo[3,2,1-ij]quinolinium and (2*E*)-*N*-[3-cyano-7-ethoxy-4-({3-methyl-4-[([1,2,4]triazolo[1,5-a]pyridin-7-yl)oxy]phenyl}amino)quinolin-6-yl]-4-(dimethylamino)but-2-enamide, respectively. The three-dimensional structures of ligands 1 and 2 were obtained from the Protein Data Bank using PDB IDs 3RHK and 7JXH, respectively. ^[b]^ Drug compound used as a reference inhibitor of biological targets. Words colored in blue, green, and red correspond to amino acids shared between compound **9** and ligands; compound **9** and the drug; and compound **9**, ligands, and the drug, respectively. Bolded residue names indicate amino acids involved in hydrogen bonding.

## Data Availability

The original contributions presented in this study are included in the article/Supplementary Material. Further inquiries can be directed to the corresponding author.

## Supplementary Materials

The following supporting information can be downloaded at: https://www.mdpi.com/article/10.3390/ph19030381/s1, (^1^H NMR, ^13^C NMR spectra of compounds **2**–**21**, Table S1. Calculated IC_50_ values of target compounds **6b**, **7f**, **7g** and **9**).

## Abbreviations

The following abbreviations are used in this manuscript:

NMR	Nuclear magnetic resonance
A549	Lung carcinoma epithelial cell model
Caco-2	Colorectal adenocarcinoma epithelial cell model
c-MET	Hepatocyte growth factor receptor
HER2	Human epidermal growth factor receptor 2
HEK293	Non-malignant human kidney cells
EGFR	Epidermal growth factor receptor
DMSO-*d*_6_	Dimethyl sulfoxide-*d*_6_
DMF	Dimethylformamide
FT-IR	Fourier-transform infrared spectroscopy
HRMS	High-resolution mass spectroscopy
FDA	U.S. Food and Drug Administration
CP	Cisplatin
DOX	Doxorubicin
MTT	3-(4,5-dimethylthiazol-2-yl)-2,5-diphenyltetrazolium bromide assay
SAR	Structure-activity relationship
