# Supplementary material for: Synthesis and Development of 3-((2,4-Difluorophenyl)Amino)Propanoic Acid Derivatives as an Antiproliferative Medicinal Chemistry Scaffold Targeting Growth Factor Receptors
Source: Pharmaceuticals (Basel). 2026 Feb 27;19(3):381. doi: 10.3390/ph19030381 (PMC13028823; doi:10.3390/ph19030381)
Supplement: Supplementary file 1 [file pharmaceuticals-19-00381-s001.zip › pharmaceuticals-4118233-supplementary.pdf]

Supplementary Materials

Synthesis and Development of 3-((2,4-difluorophenyl)amino)propanoic Acid Derivatives as an Antiproliferative Medicinal Chemistry Scaffold Targeting Growth Factor Receptors

Guoda Pranaitytė [1], Povilas Kavaliauskas [1,2,4], Vidmantas Petraitis [3], Ruta Petraitiene [3], Ramune Grigaleviciute [4,5] Liudas Ivanauskas [6], Mindaugas Marksa [6], Gediminas Duda [6], Waldo Acevedo [7], Birutė Grybaitė [1,*] and Vytautas Mickevičius [1]

1 Department of Organic Chemistry, Kaunas University of Technology, Radvilenų Rd. 19, LT-50254 Kaunas, Lithuania; guoda.pranaityte@ktu.lt, birute.grybaite@ktu.lt, vytautas.mickevicius@ktu.lt
2 Department of Microbiology and Immunology, University of Maryland School of Medicine, Baltimore, Maryland 21201, United States; Povilas.Kavaliauskas@som.umaryland.edu
3 Center for Discovery and Innovation, Hackensack Meridian Health, Nutley, NJ 07110, USA.
4 Biological Research Center, Lithuanian University of Health Sciences, LT-44307 Kaunas, Lithuania.
5 Department of Animal Nutrition, Lithuanian University of Health Sciences, LT-44307 Kaunas, Lithuania
6 Department of Analytical and Toxicological Chemistry, Lithuanian University of Health Sciences, LT-50161 Kaunas, Lithuania; mindaugas.marksa@lsmu.lt, liudas.ivanauskas@lsmu.lt, gedas957@gmail.com
7 Instituto de Química, Facultad de Ciencias, Pontificia Universidad Católica de Valparaíso – Valparaíso, Chile; waldo.acevedo@pucv.cl
* Correspondence: birute.grybaite@ktu.lt

*3-[(Difluorophenyl)amino]propanoic acid (**2**)*

Figure S1. ^{1}H NMR spectrum of compound **2.**

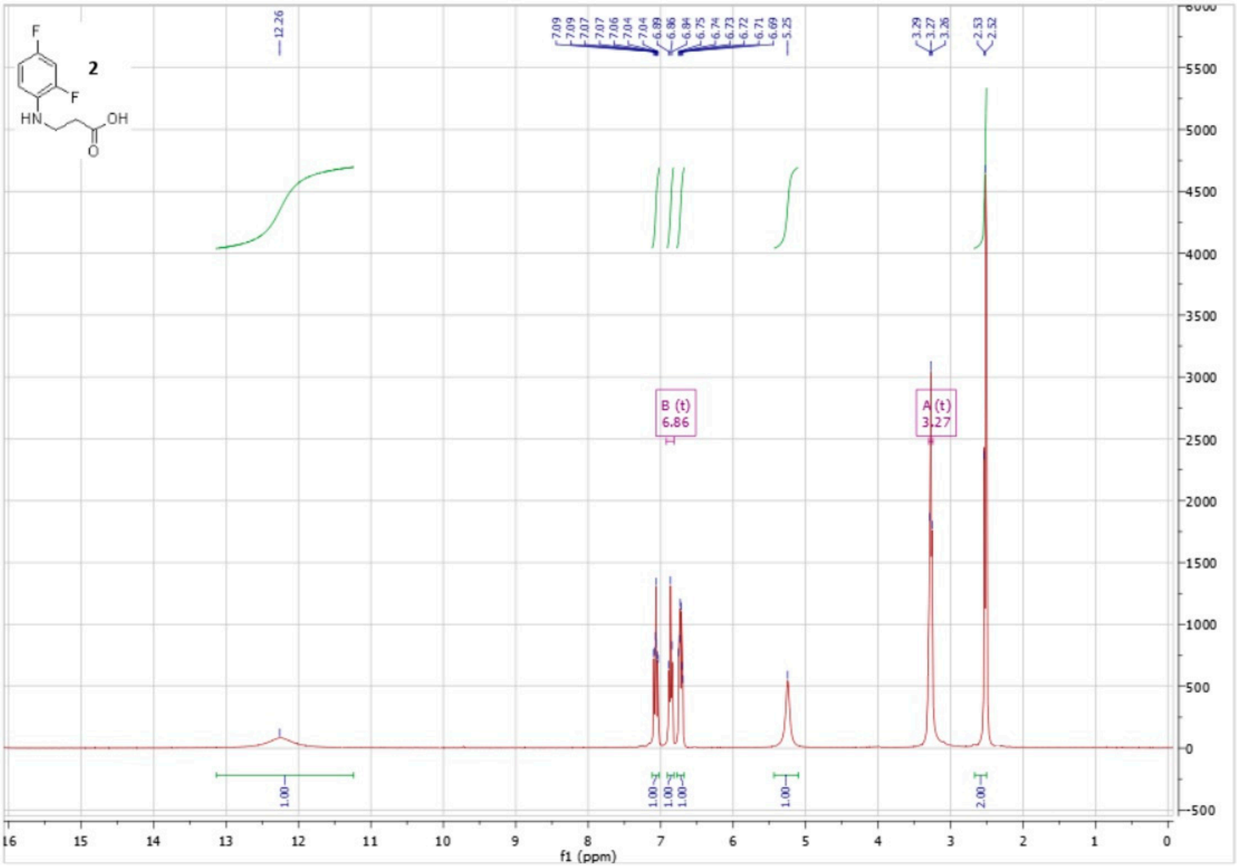

Figure S2. ^{13}C NMR spectrum of compound **2.**

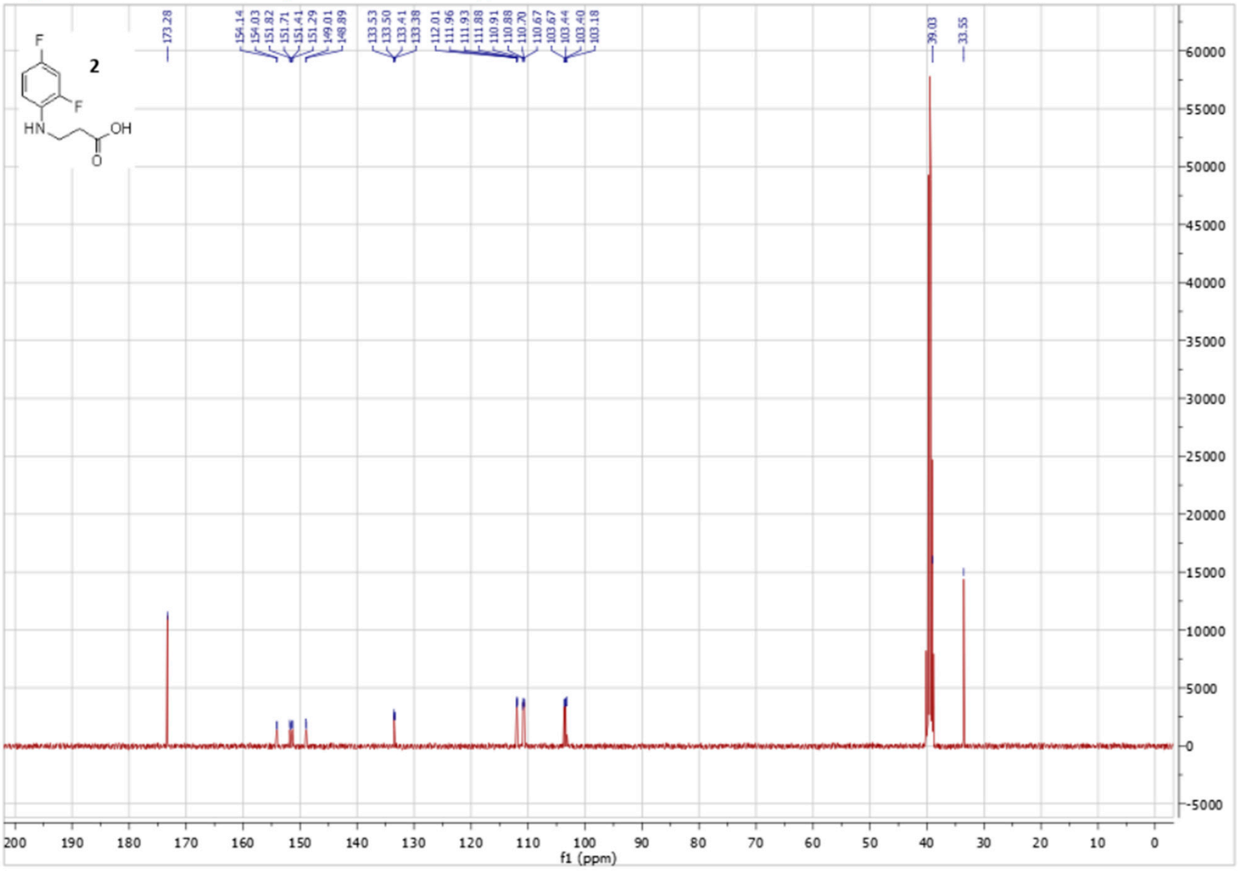

*Methyl 3-[(2,4-difluorophenyl)amino]propanoate (**3**)*

Figure S3. ^{1}H NMR spectrum of compound **3.**

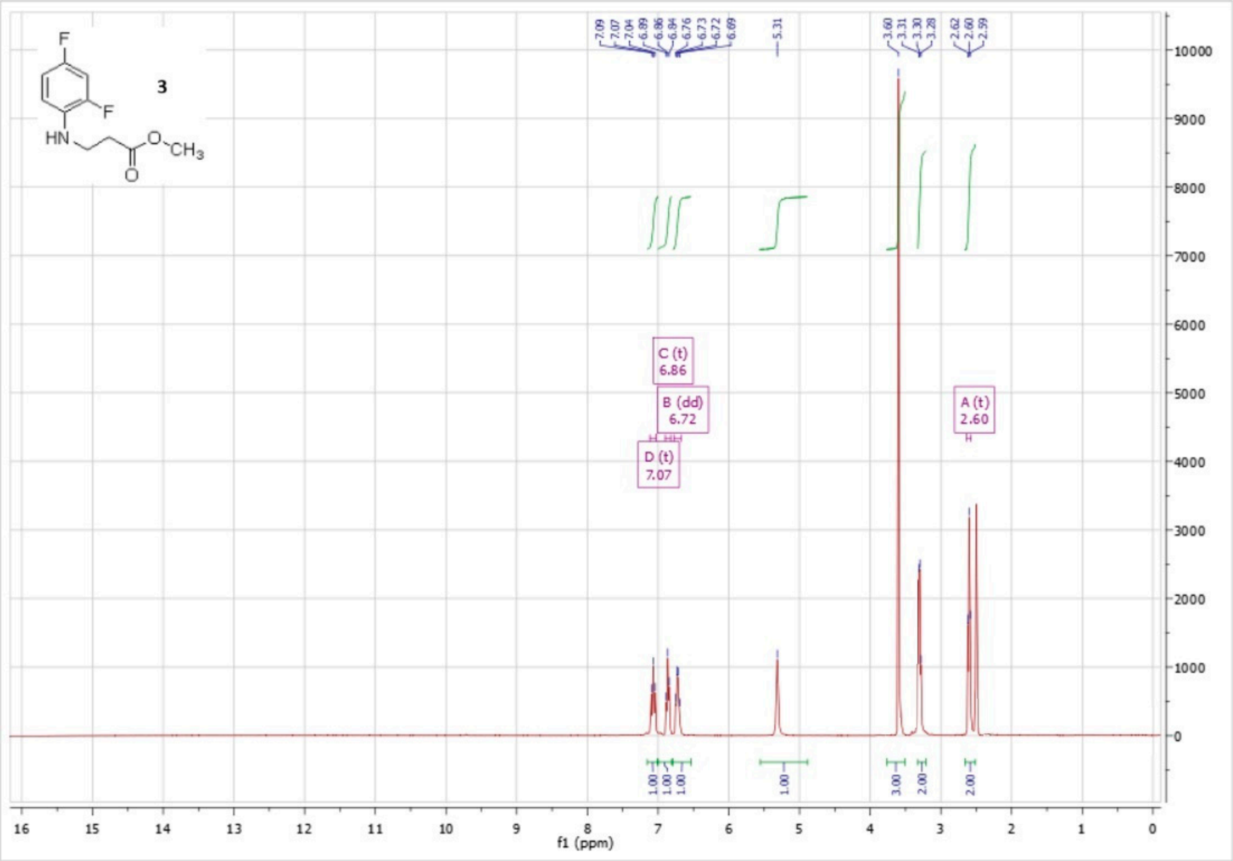

Figure S4. ^{13}C NMR spectrum of compound **3.**

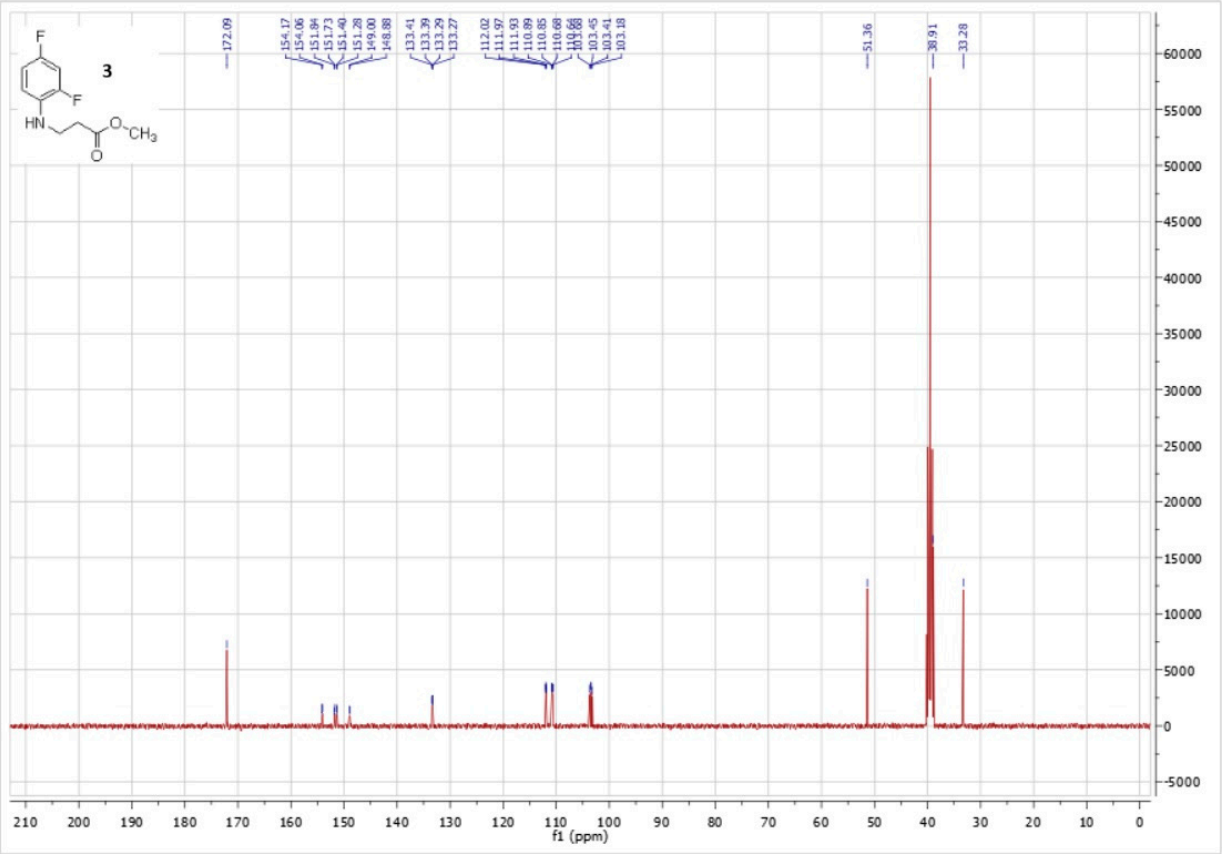

*3-[(2,4-Difluorophenyl)amino]propanehydrazide (**4**)*

Figure S5. ^{1}H NMR spectrum of compound **4.**

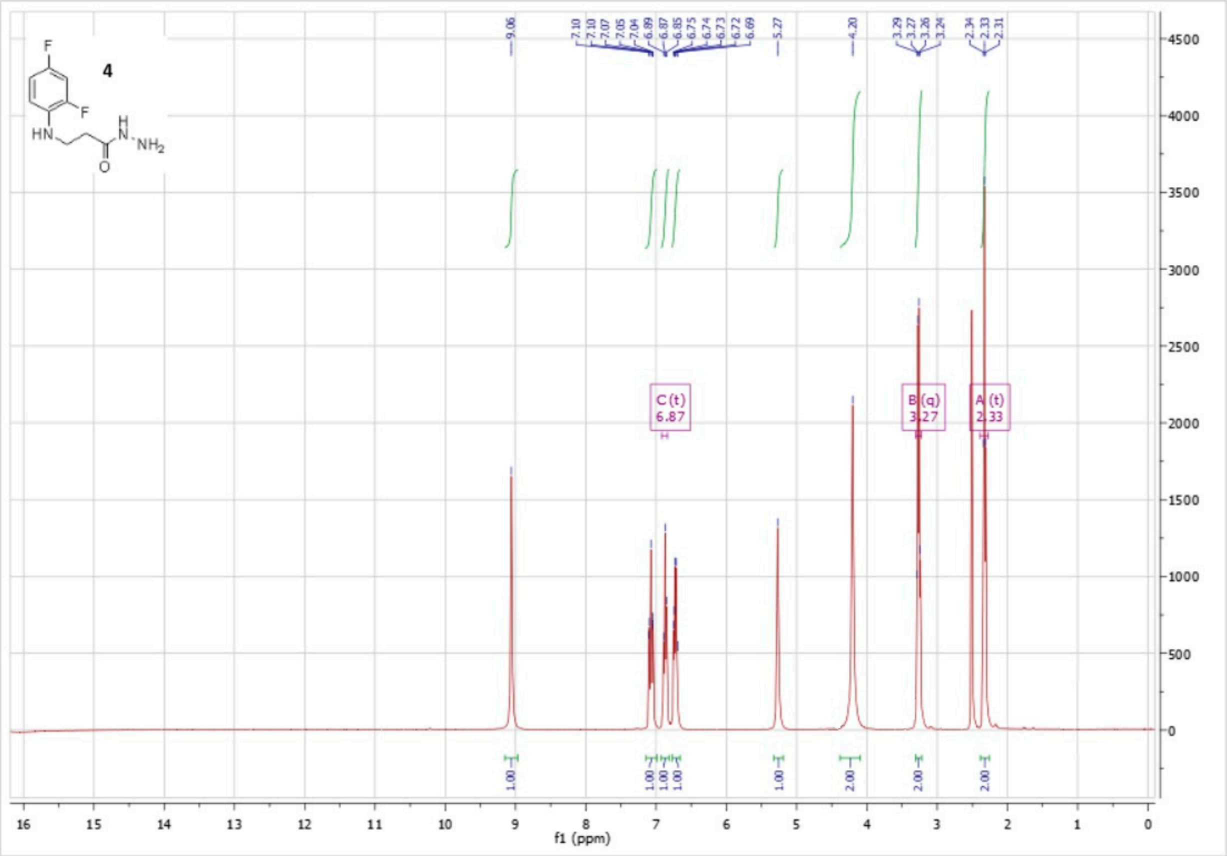

Figure S6. ^{13}C NMR spectrum of compound **4.**

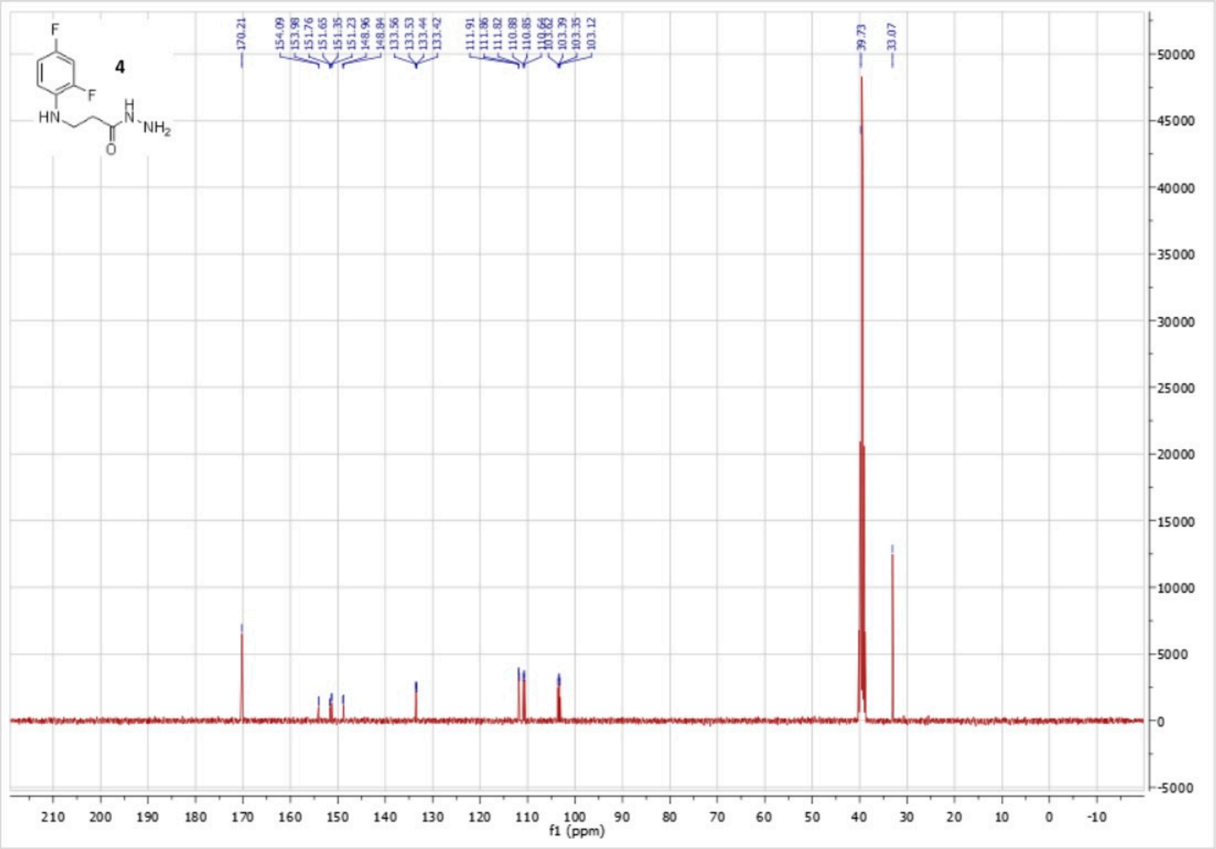

*3-[(2,4-Difluorophenyl)amino]-N'-(thiophen-2-ylmethylene)propanehydrazide (**5a**)*

Figure S7. ^{1}H NMR spectrum of compound **5a.**

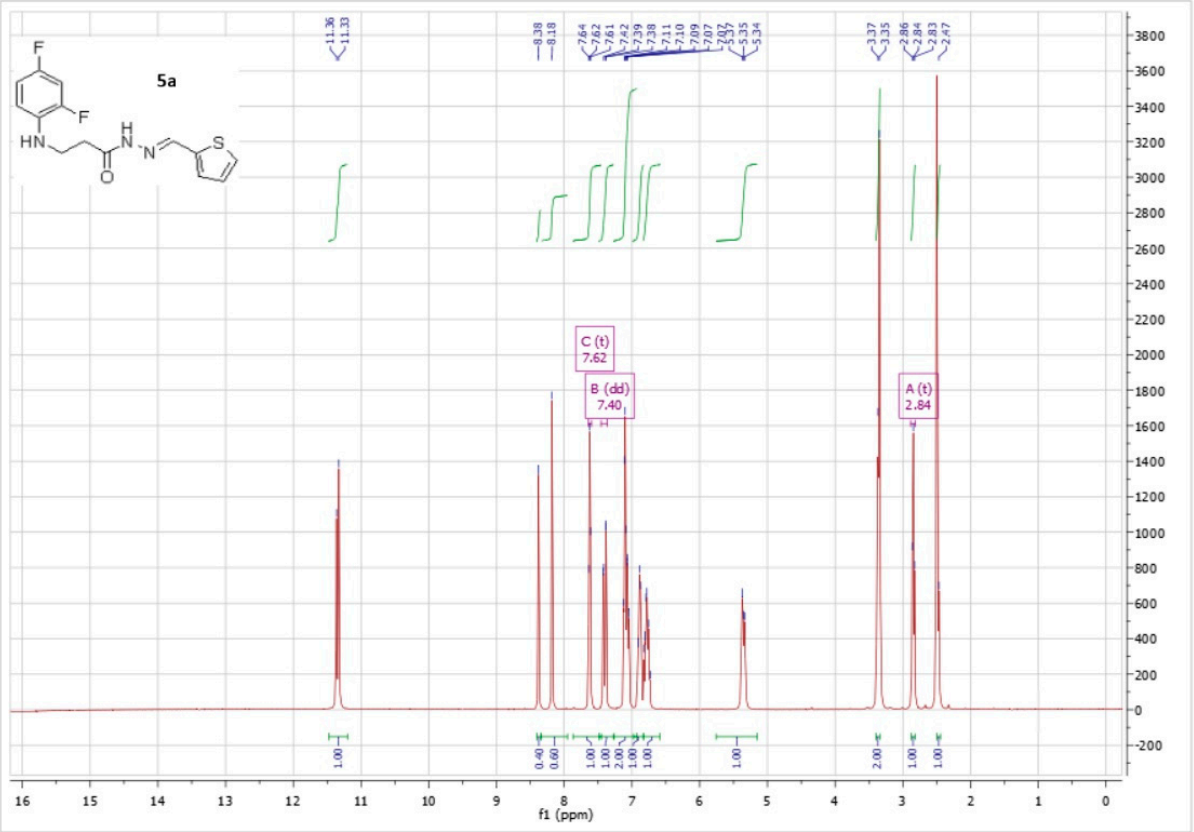

Figure S8. ^{13}C NMR spectrum of compound **5a.**

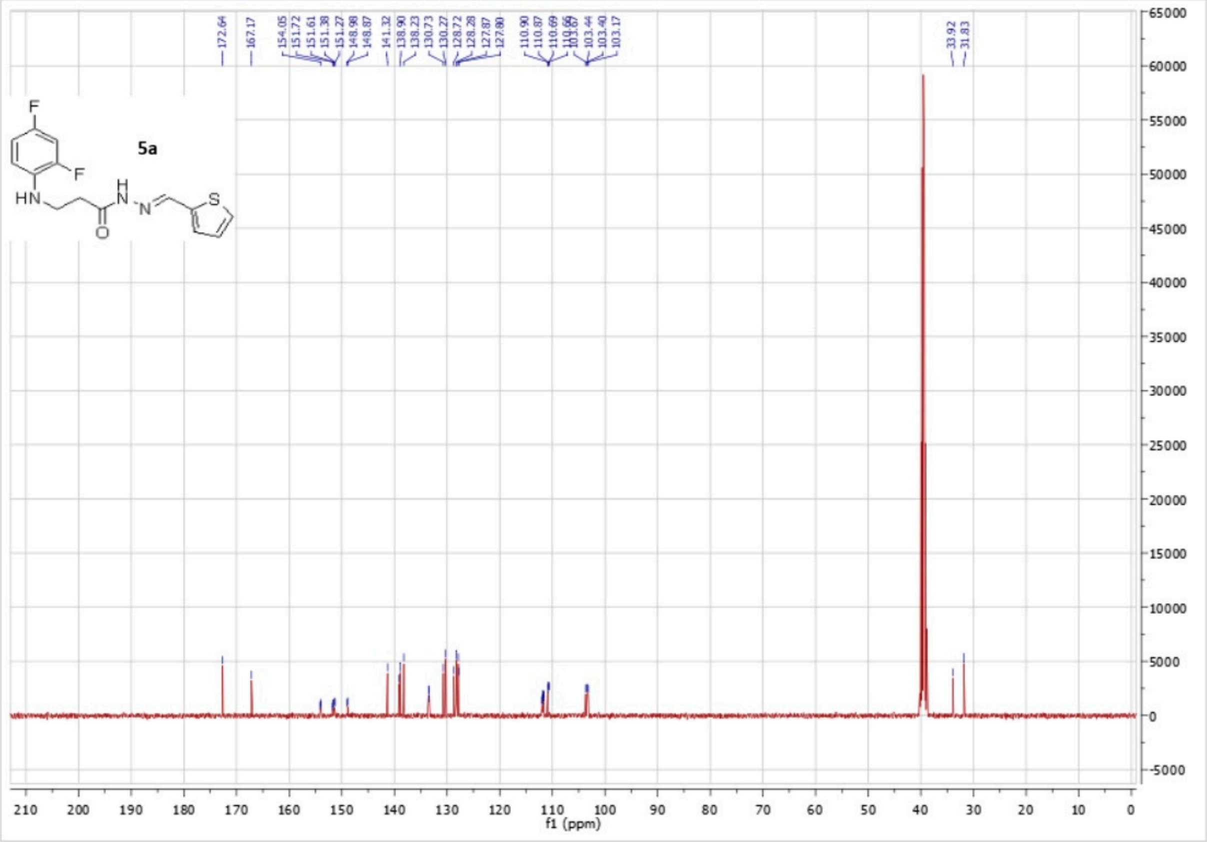

*3-[(2,4-Difluorophenyl)amino]-N'-[(5-nitrothiophen-2-yl)methylene]propanehydrazide (**5b**)*

Figure S9. ^{1}H NMR spectrum of compound **5b.**

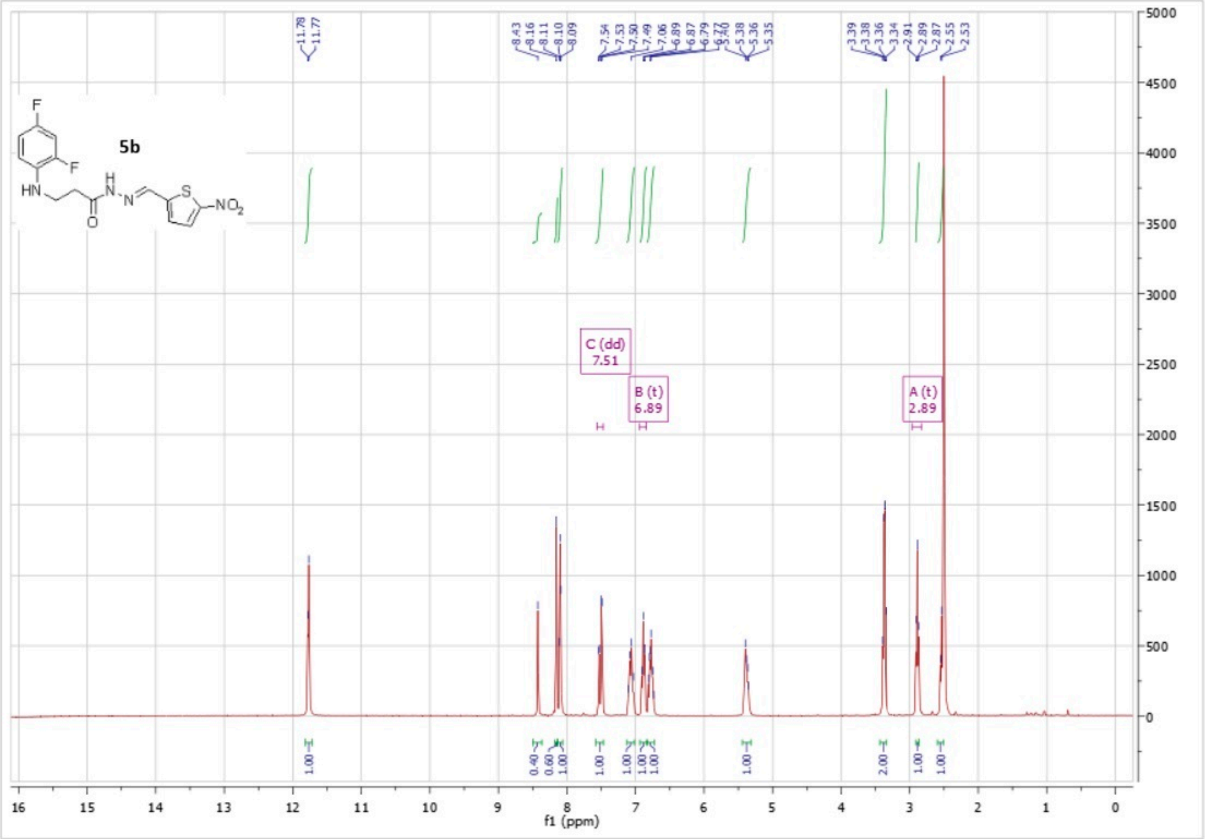

Figure S10. ^{13}C NMR spectrum of compound **5b.**

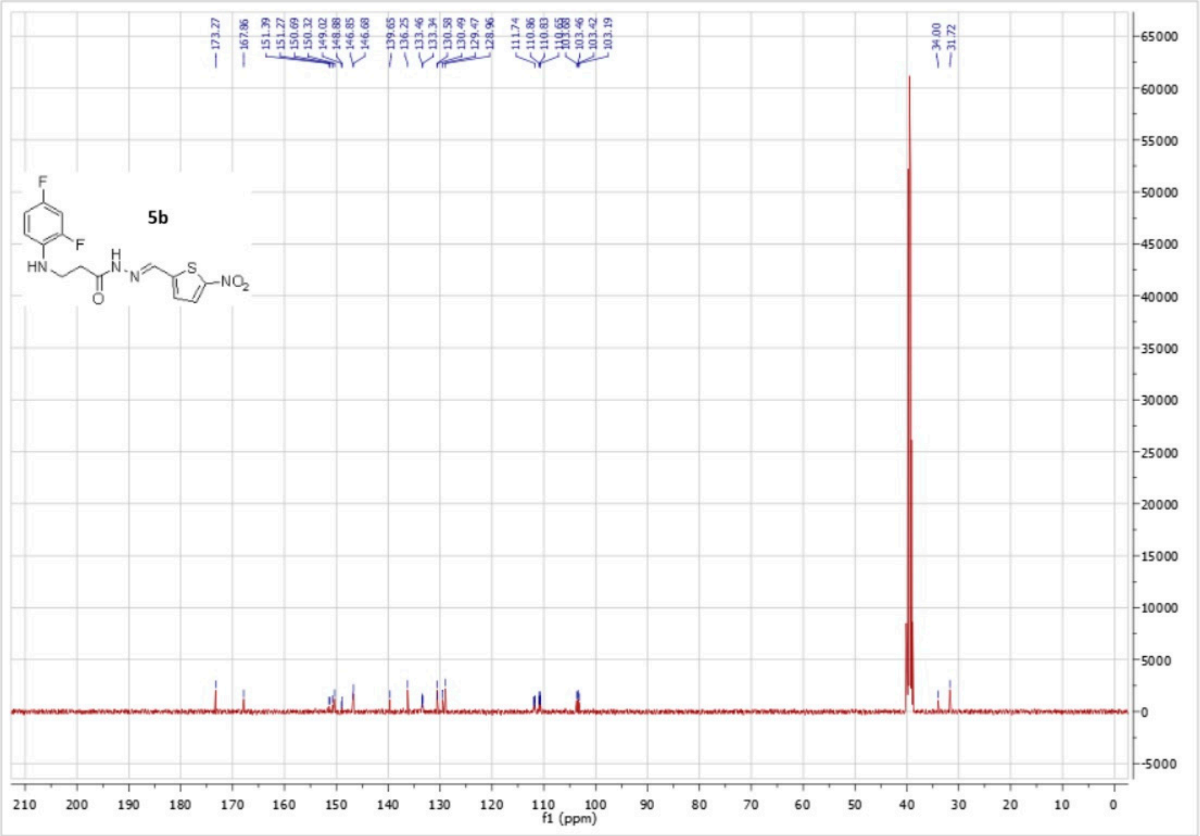

*N'-[(4-Bromothiophen-2-yl)methylene]-3-[(2,4-difluorophenyl)amino]propanehydrazide (**5c**)*

Figure S11. ^{1}H NMR spectrum of compound **5c.**

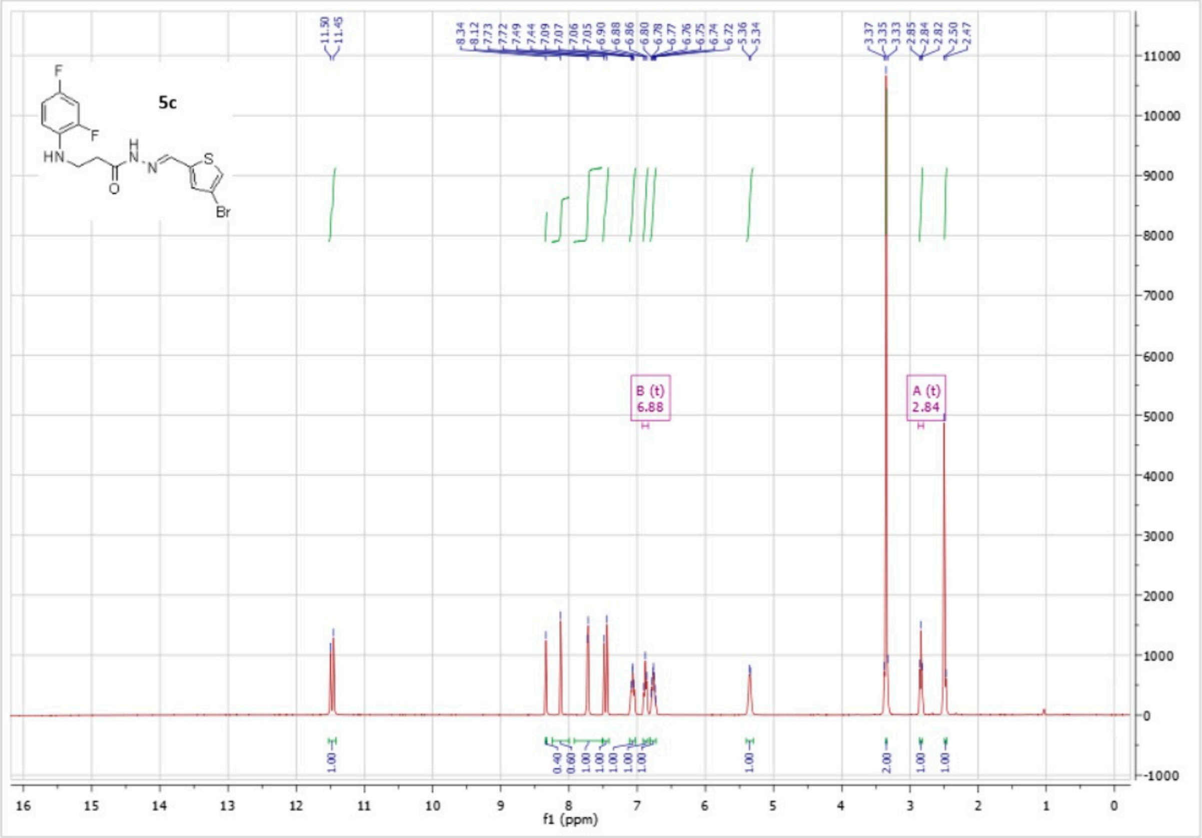

Figure S12. ^{13}C NMR spectrum of compound **5c.**

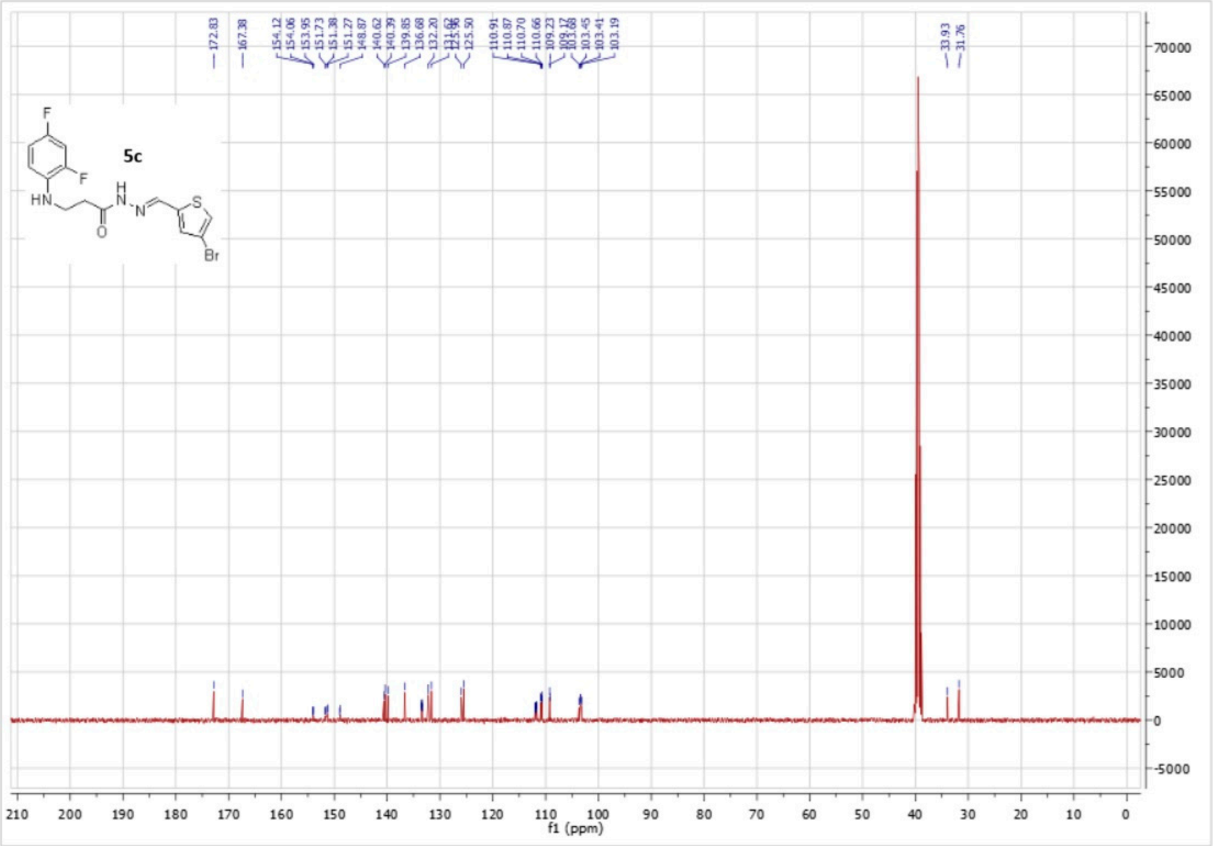

*3-[(2,4-Difluorophenyl)amino]-N'-(furan-2-ylmethylene)propanehydrazide (**6a**)*

Figure S13. ^{1}H NMR spectrum of compound **6a.**

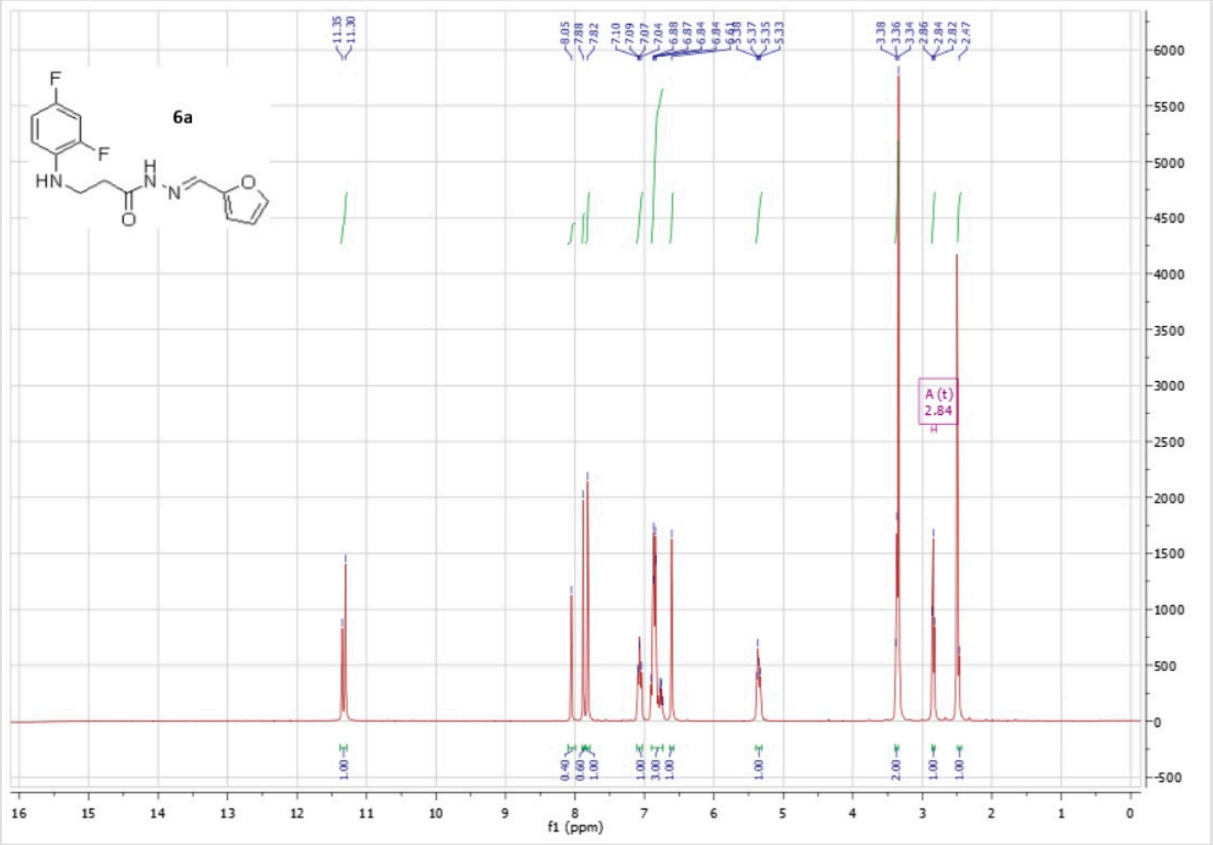

Figure S14. ^{13}C NMR spectrum of compound **6a.**

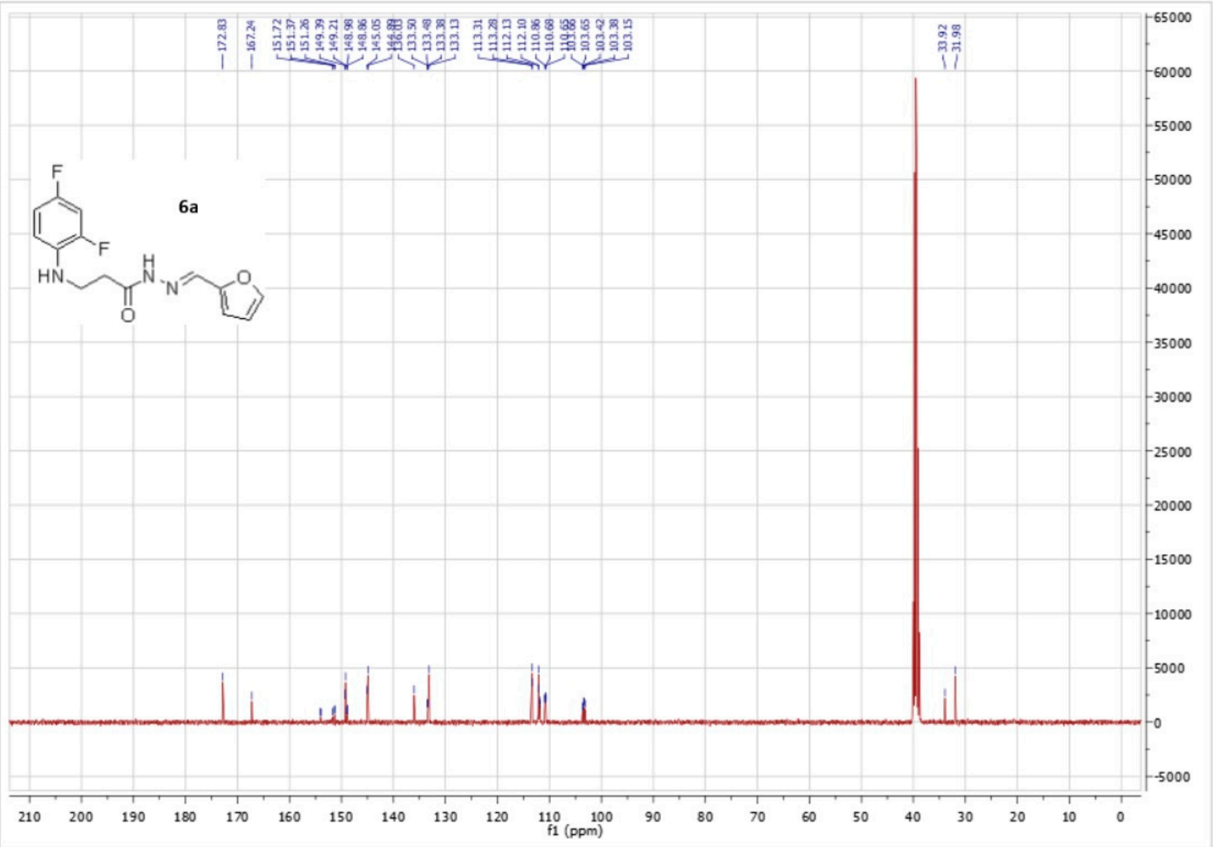

*3-[(2,4-Difluorophenyl)amino]-N'-[(5-nitrofuran-2-yl)methylene]propanehydrazide (**6b**)*

Figure S15. ^{1}H NMR spectrum of compound **6b.**

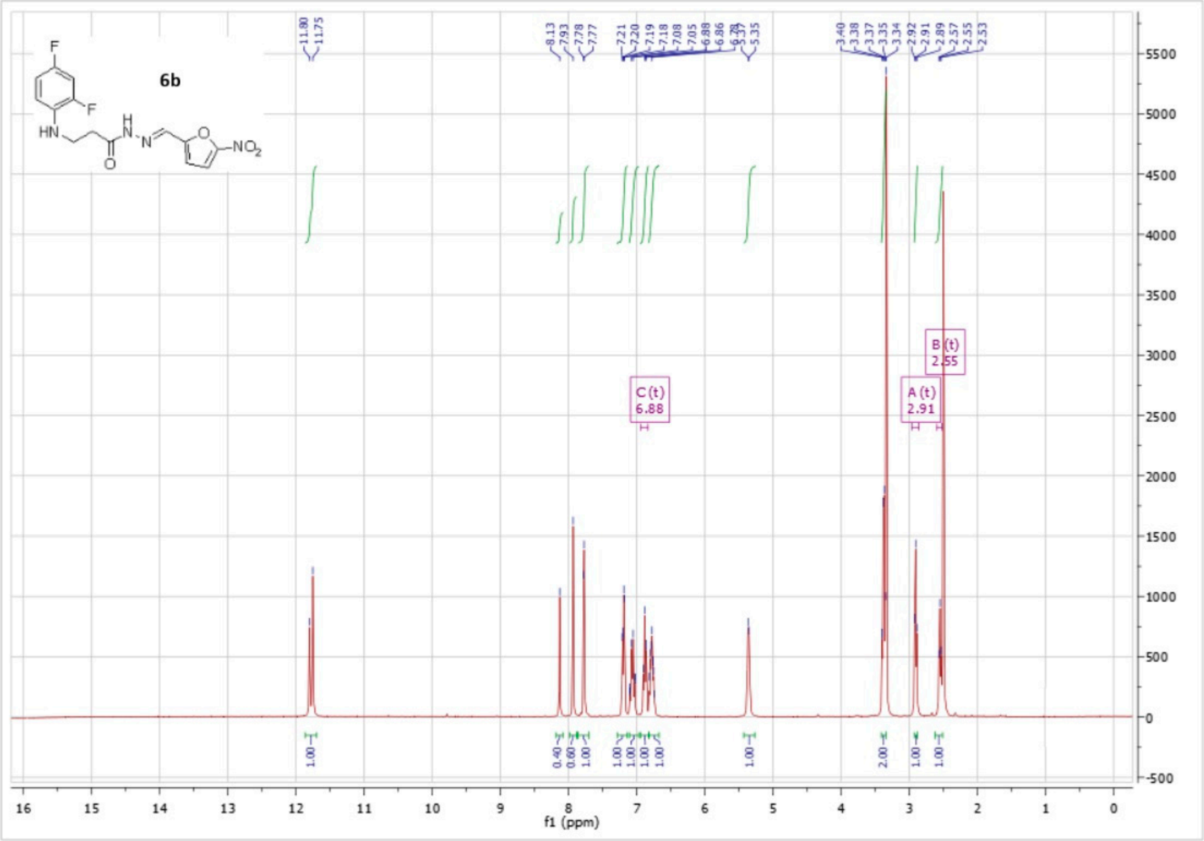

Figure S16. ^{13}C NMR spectrum of compound **6b.**

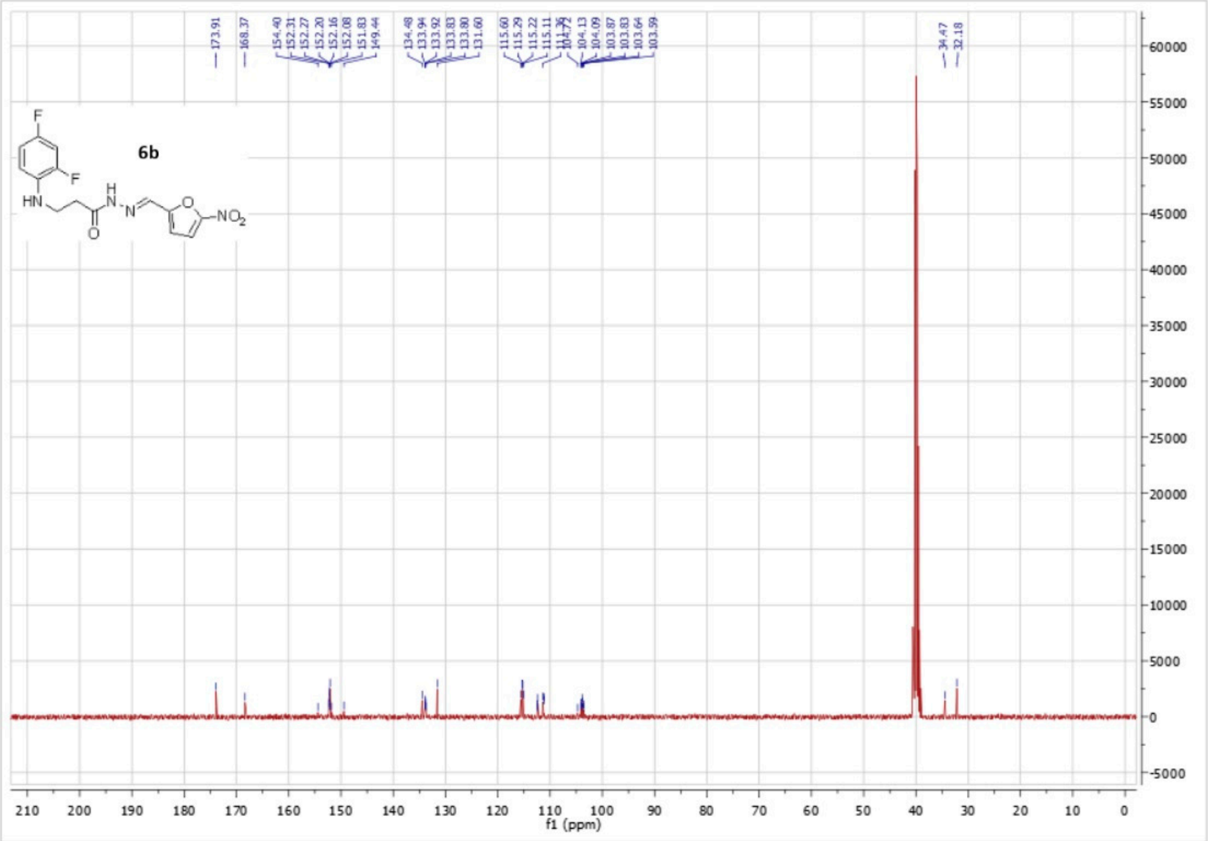

*N'-[(5-Bromofuran-2-yl)methylene]-3-[(2,4-difluorophenyl)amino]propanehydrazide (**6c**)*

Figure S17. ^{1}H NMR spectrum of compound **6c.**

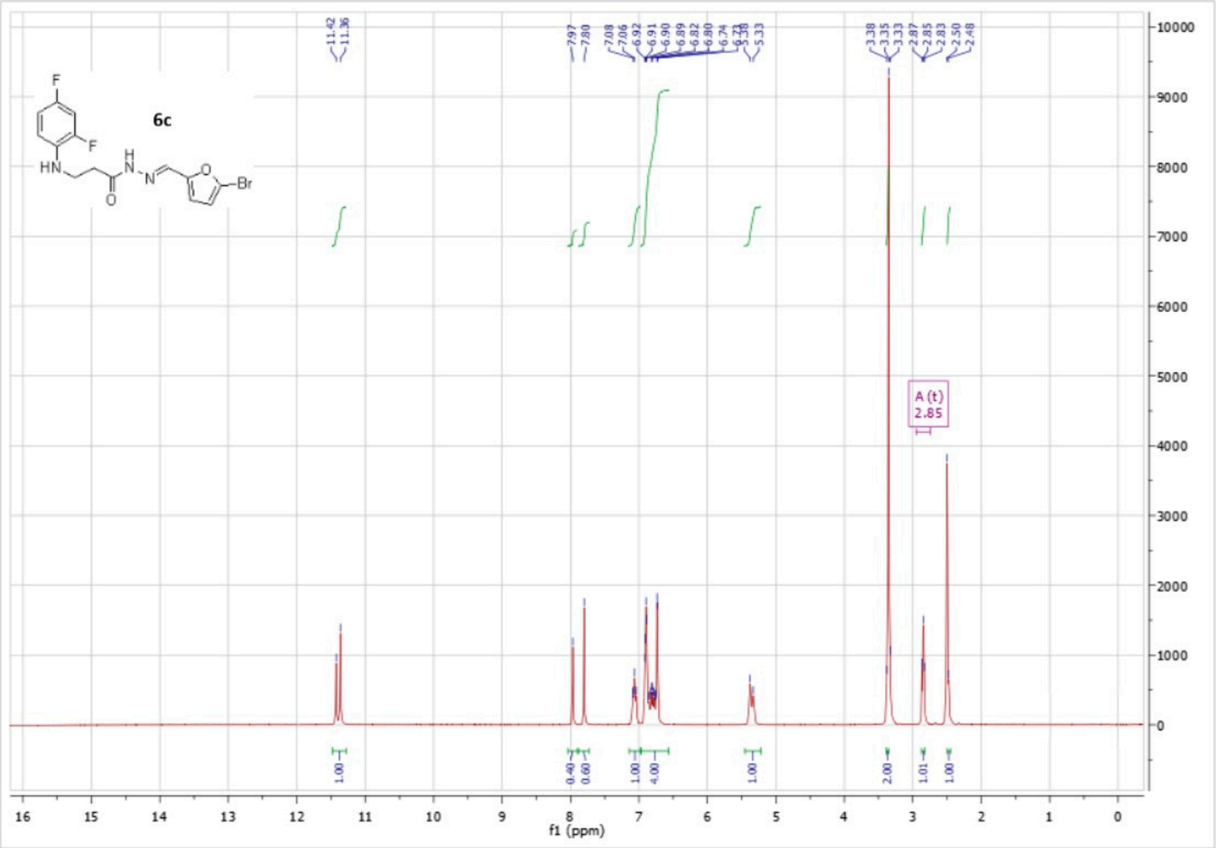

Figure S18. ^{13}C NMR spectrum of compound **6c.**

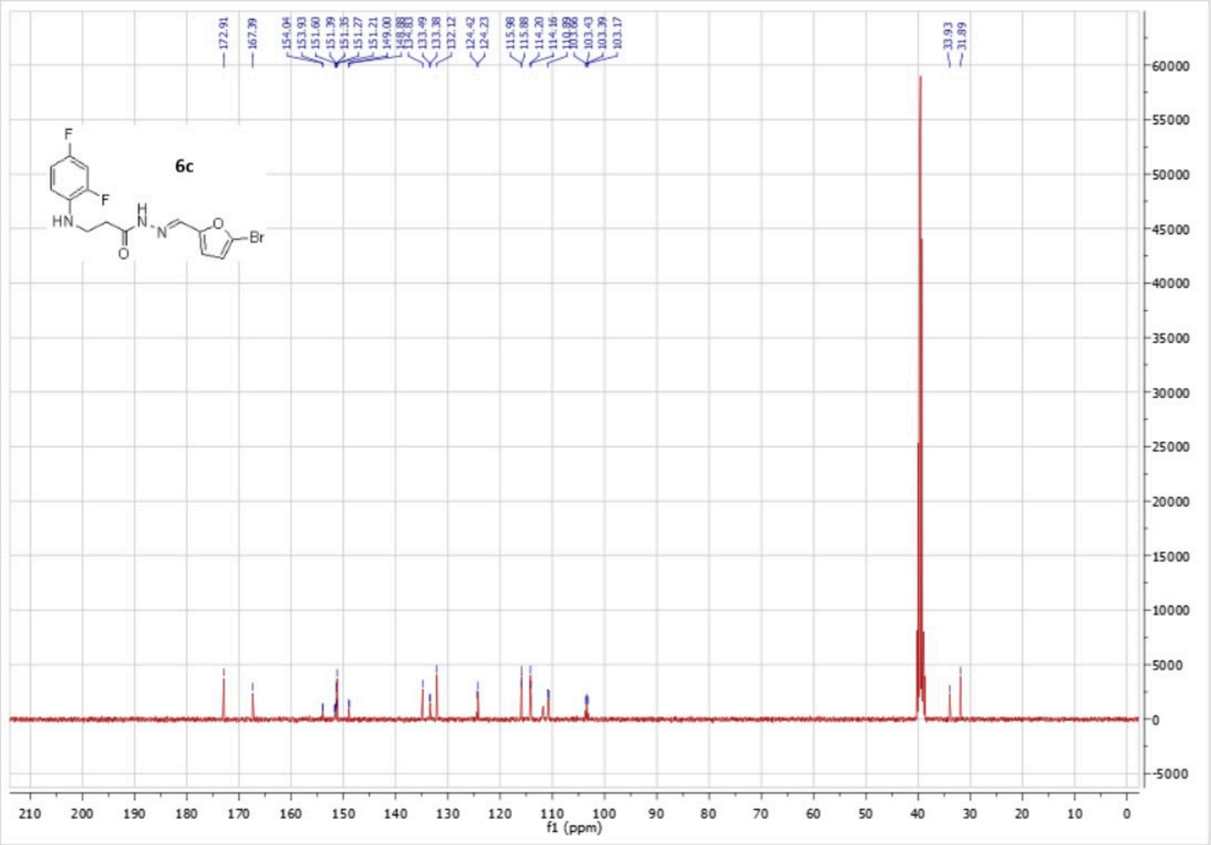

*N'-Benzylidene-3-[(2,4-difluorophenyl)amino]propanehydrazide (7**a**)*

Figure S19. ^{1}H NMR spectrum of compound **7a.**

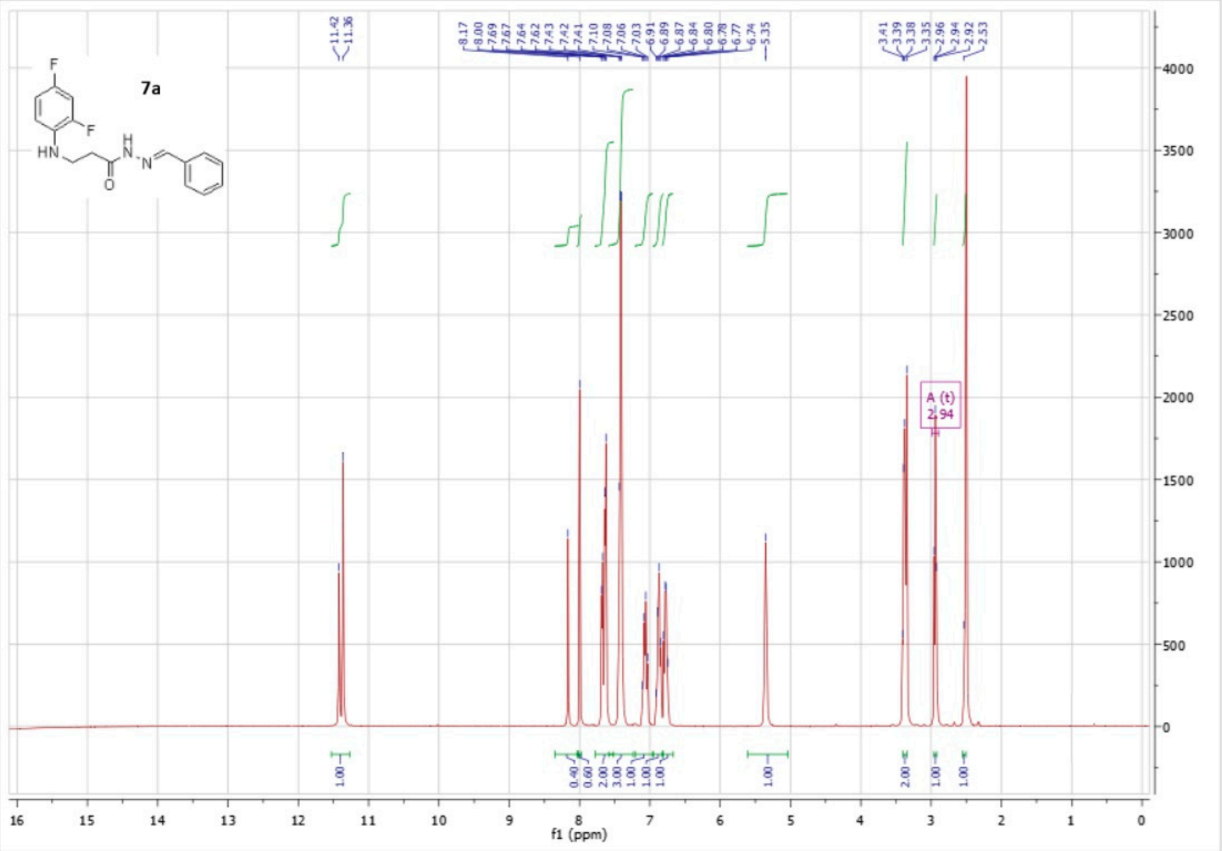

Figure S20. ^{13}C NMR spectrum of compound **7a.**

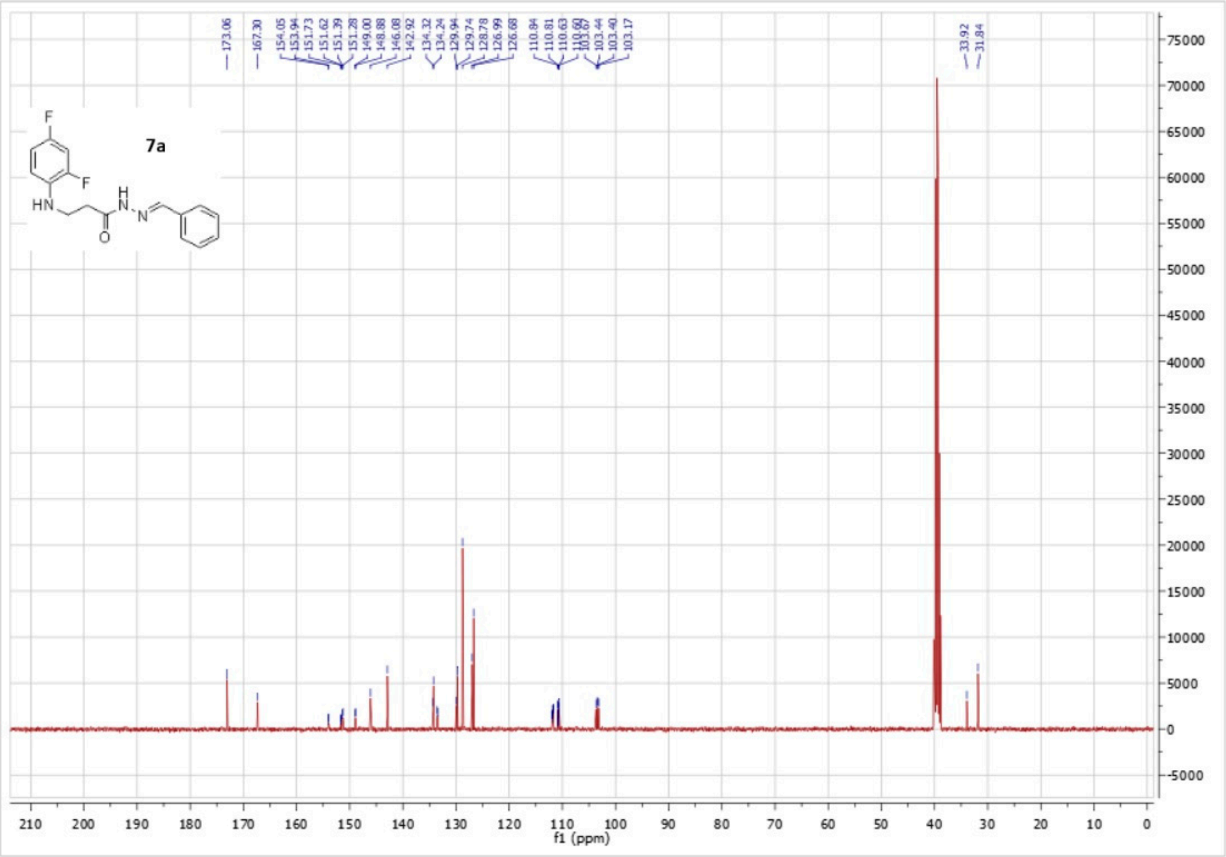

*3-[(2,4-Difluorophenyl)amino]-N'-(4-fluorobenzylidene)propanehydrazide (7**b**)*

Figure S21. ^{1}H NMR spectrum of compound **7b.**

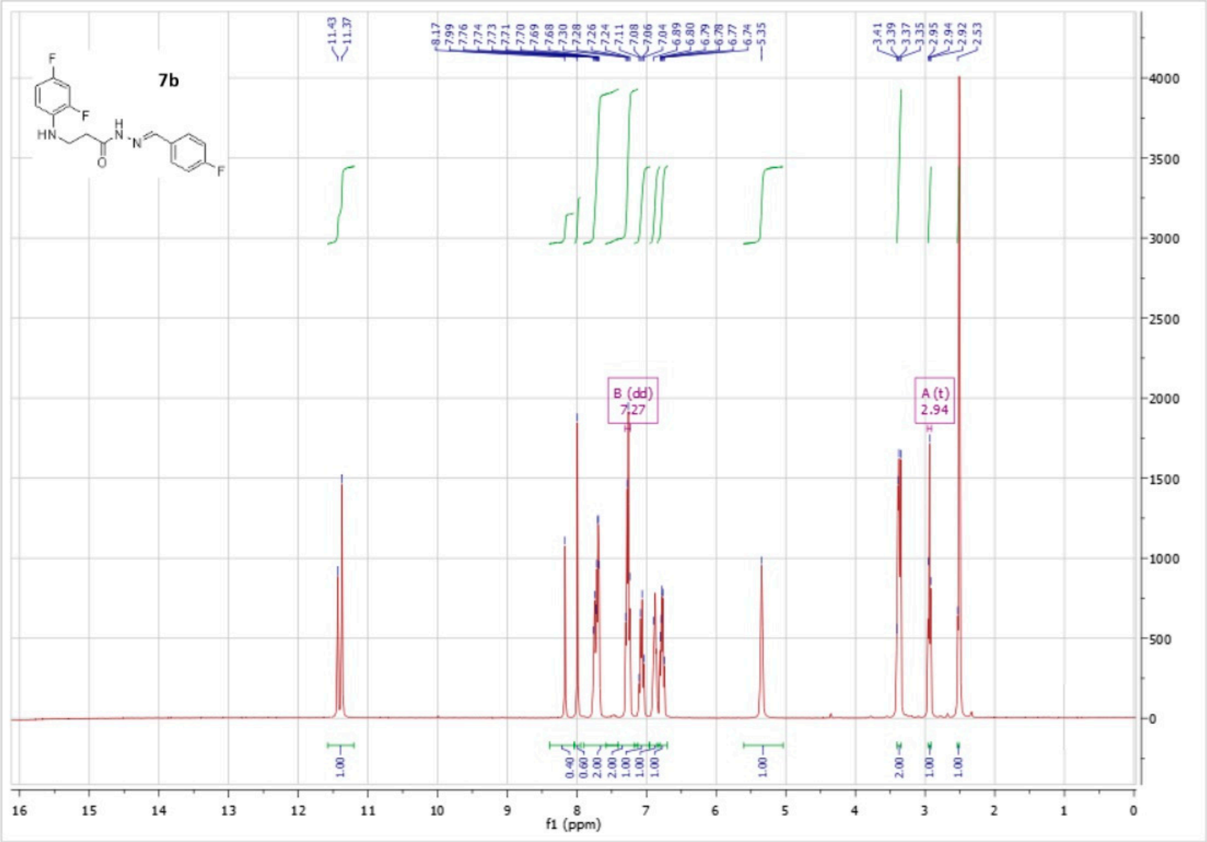

Figure S22. ^{13}C NMR spectrum of compound **7b.**

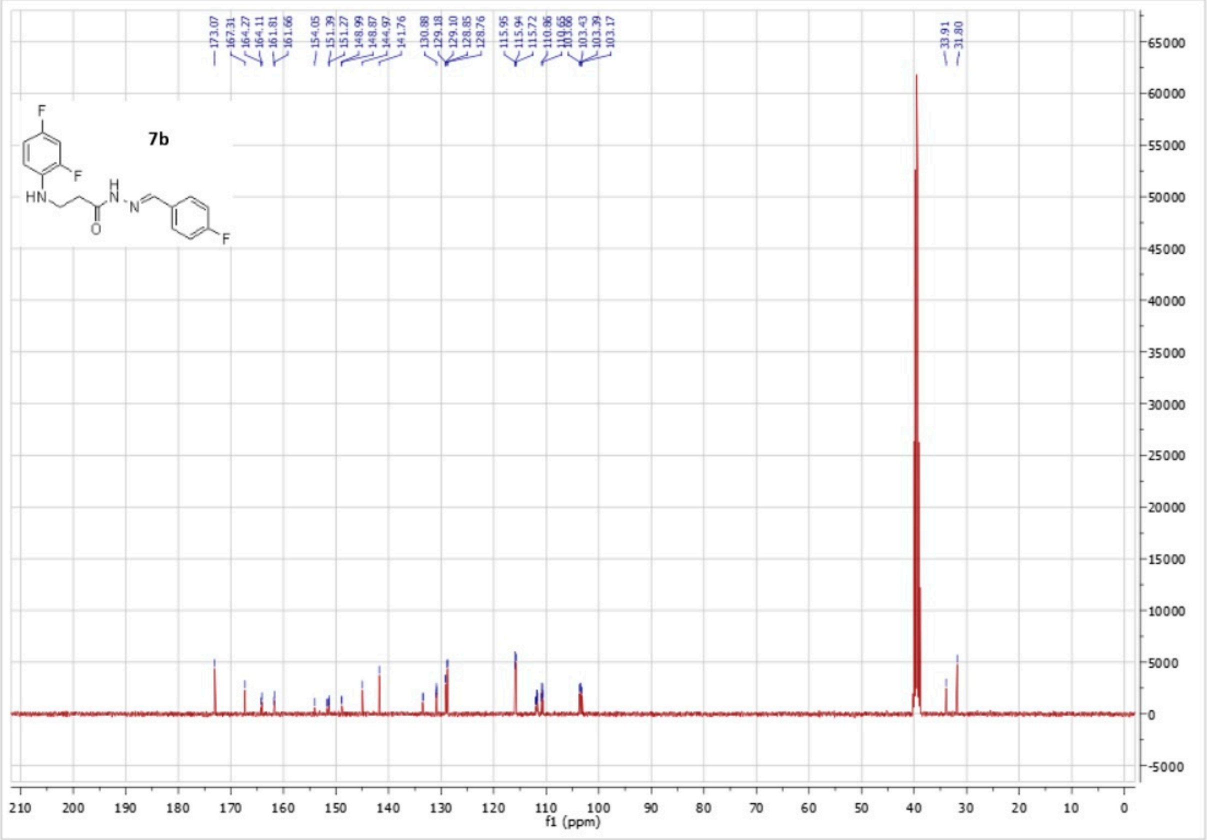

*N'-(2,4-Difluorobenzylidene)-3-[(2,4-difluorophenyl)amino]propanehydrazide (7**c**)*

Figure S23. ^{1}H NMR spectrum of compound **7c.**

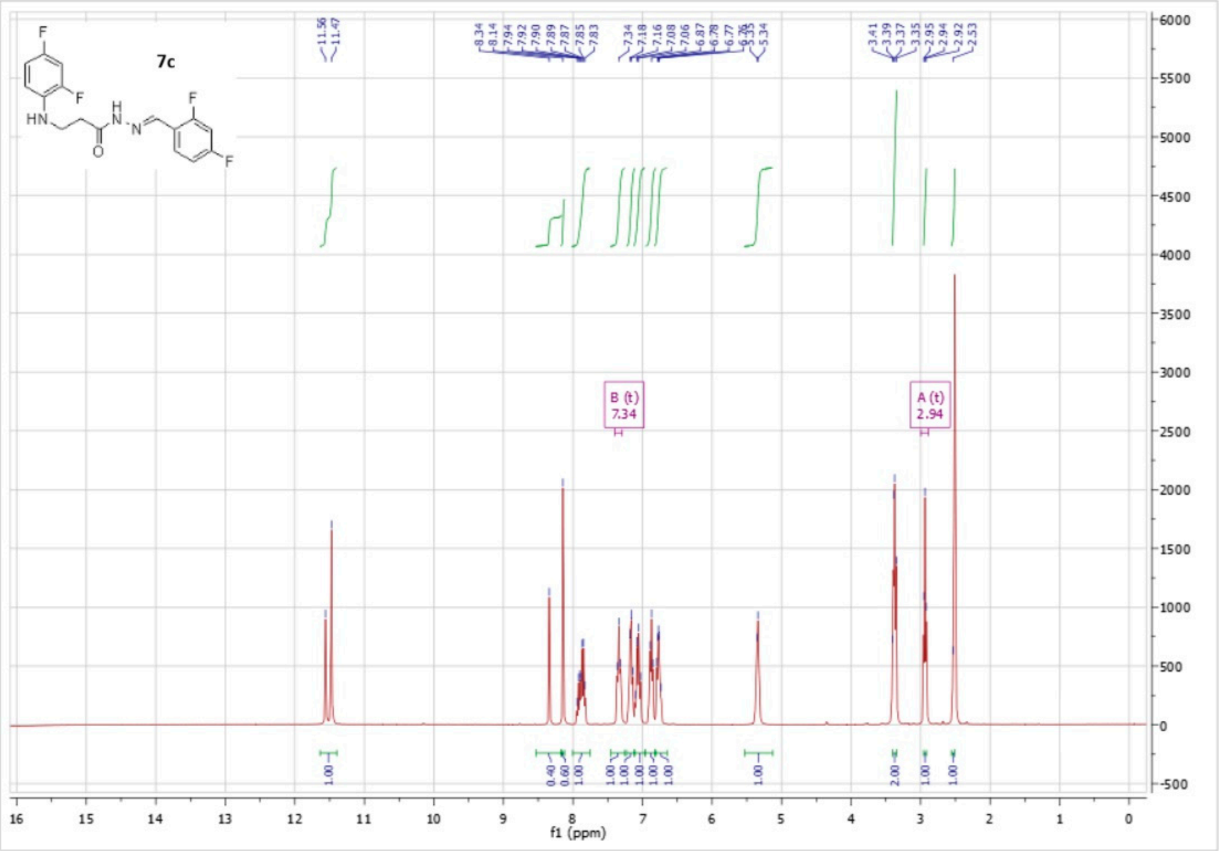

Figure S24. ^{13}C NMR spectrum of compound **7c.**

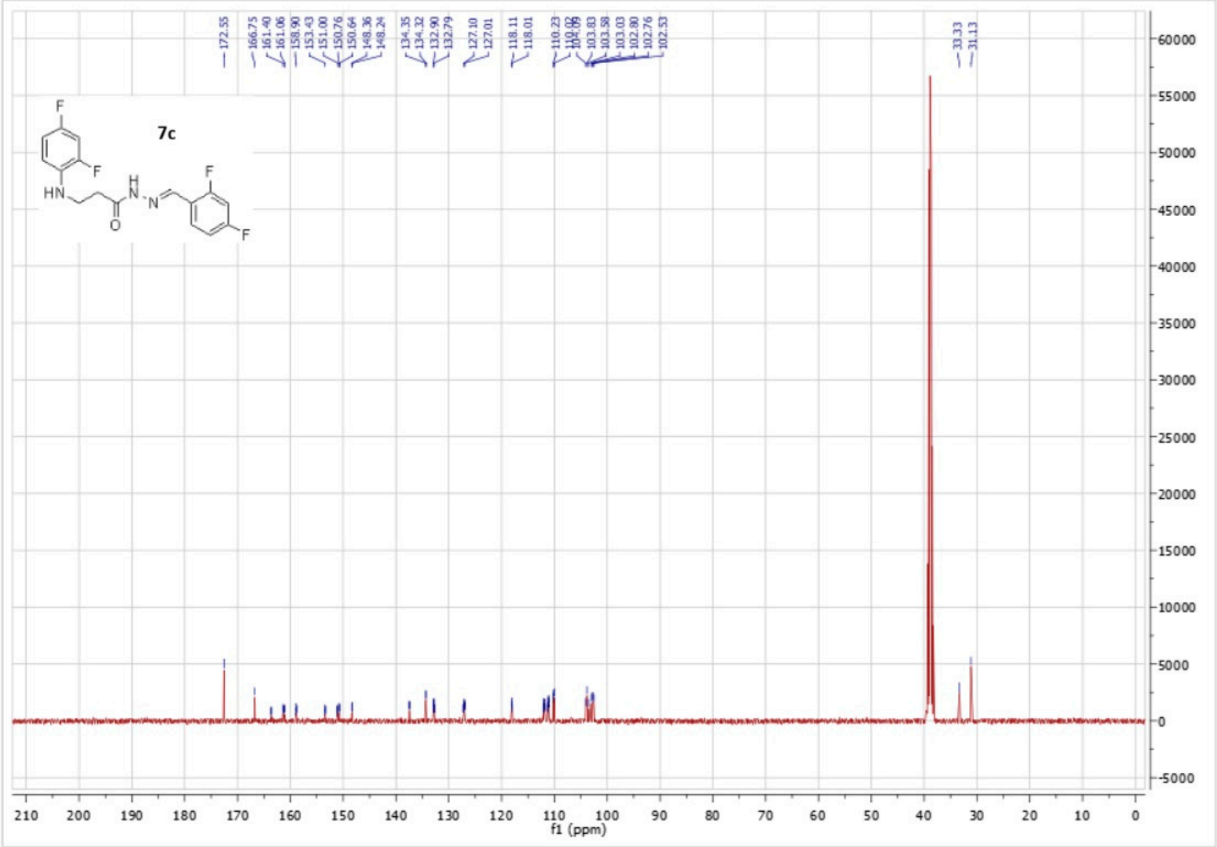

*N'-(4-Chlorobenzylidene)-3-[(2,4-difluorophenyl)amino]propanehydrazide (7**d**)*

Figure S25. ^{1}H NMR spectrum of compound **7d.**

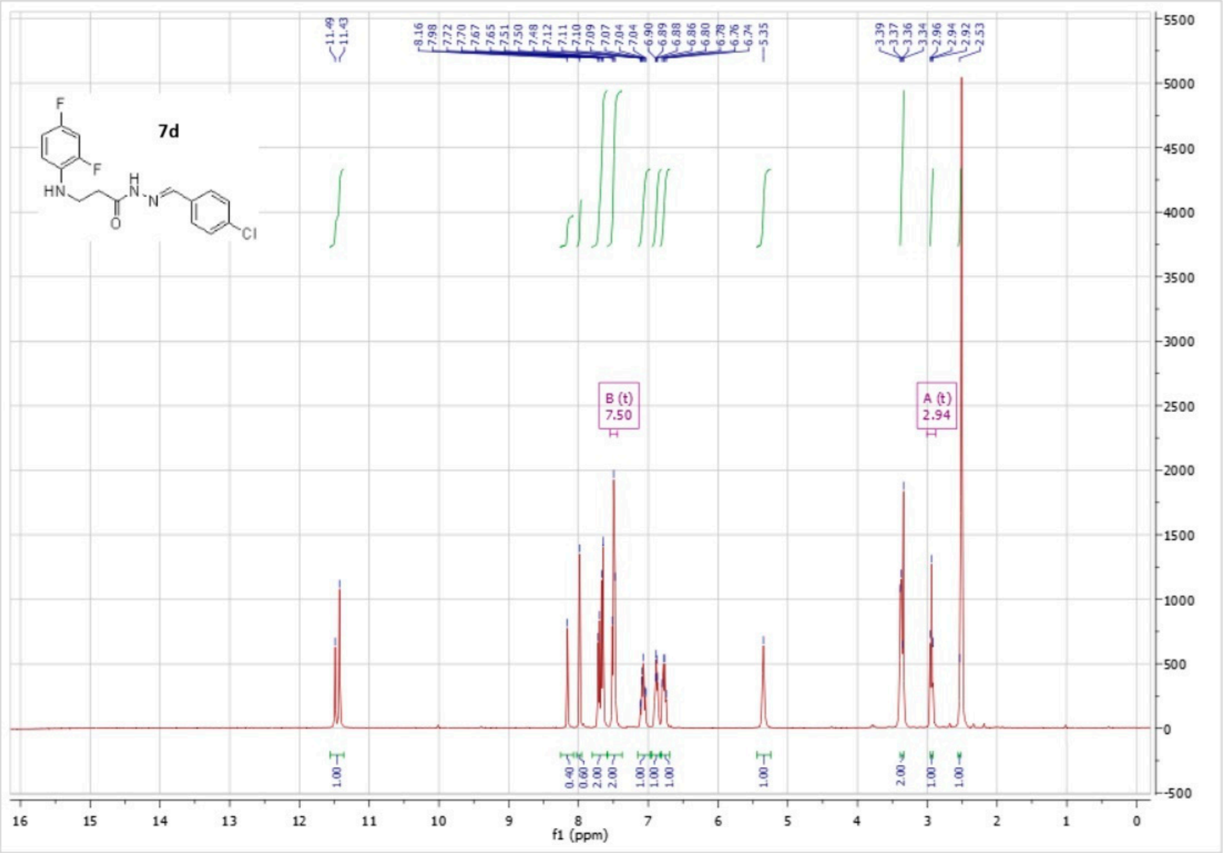

Figure S26. ^{13}C NMR spectrum of compound **7d.**

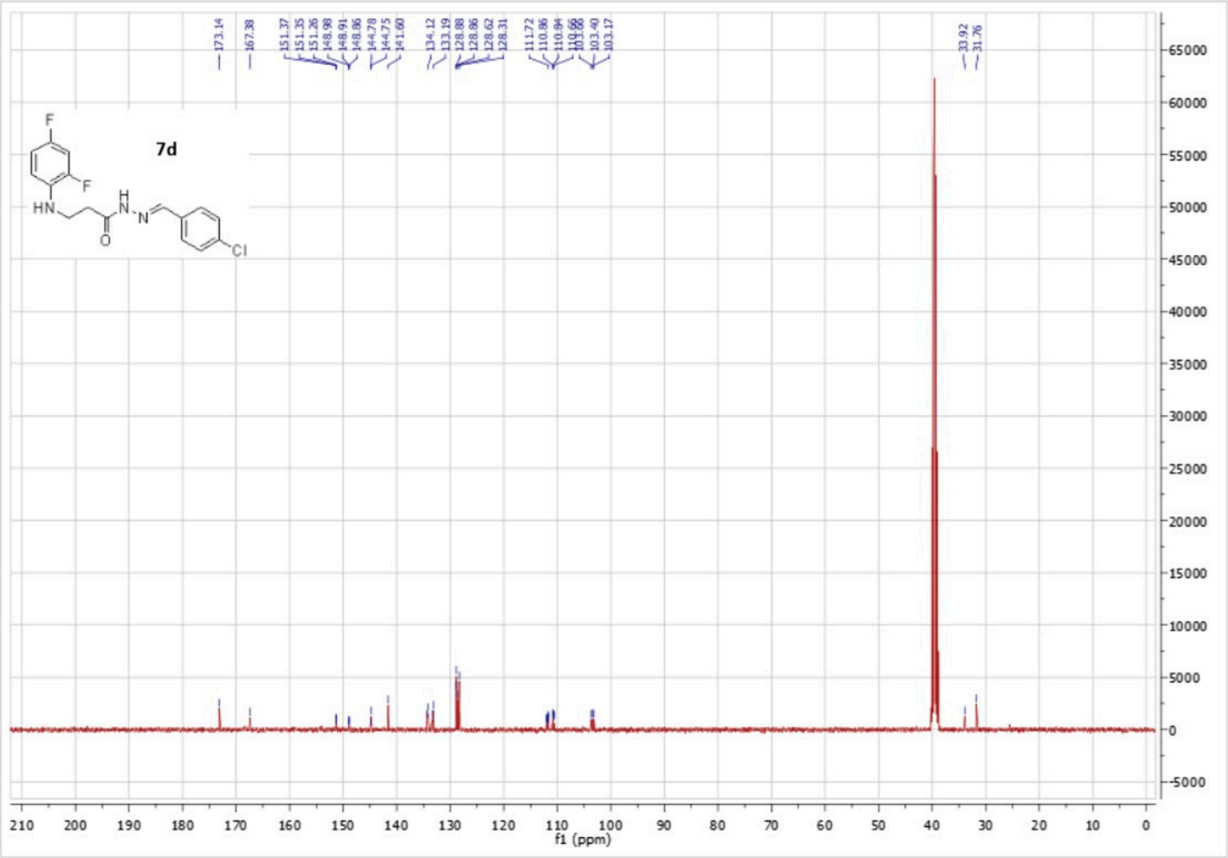

*N'-(4-Bromobenzylidene)-3-[(2,4-difluorophenyl)amino]propanehydrazide (7**e**)*

Figure S27. ^{1}H NMR spectrum of compound **7e.**

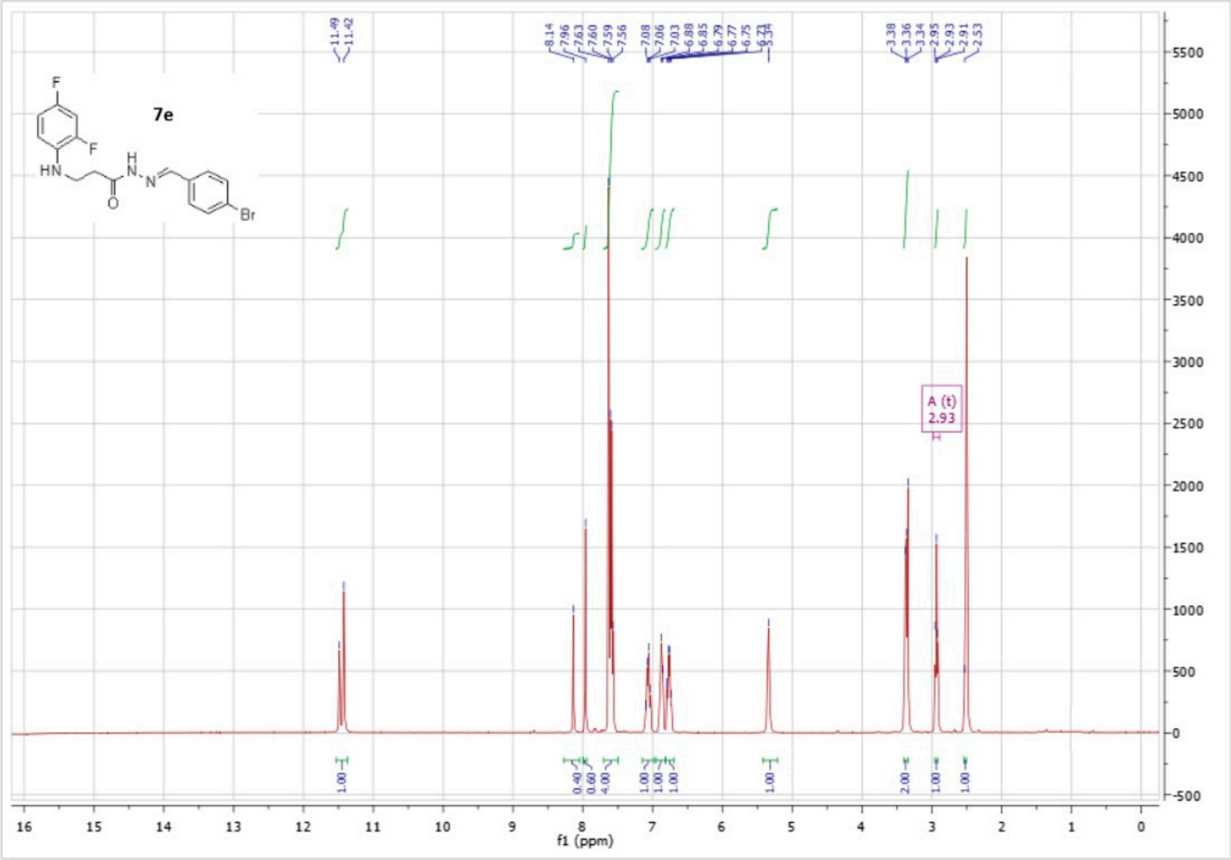

Figure S28. ^{13}C NMR spectrum of compound **7e.**

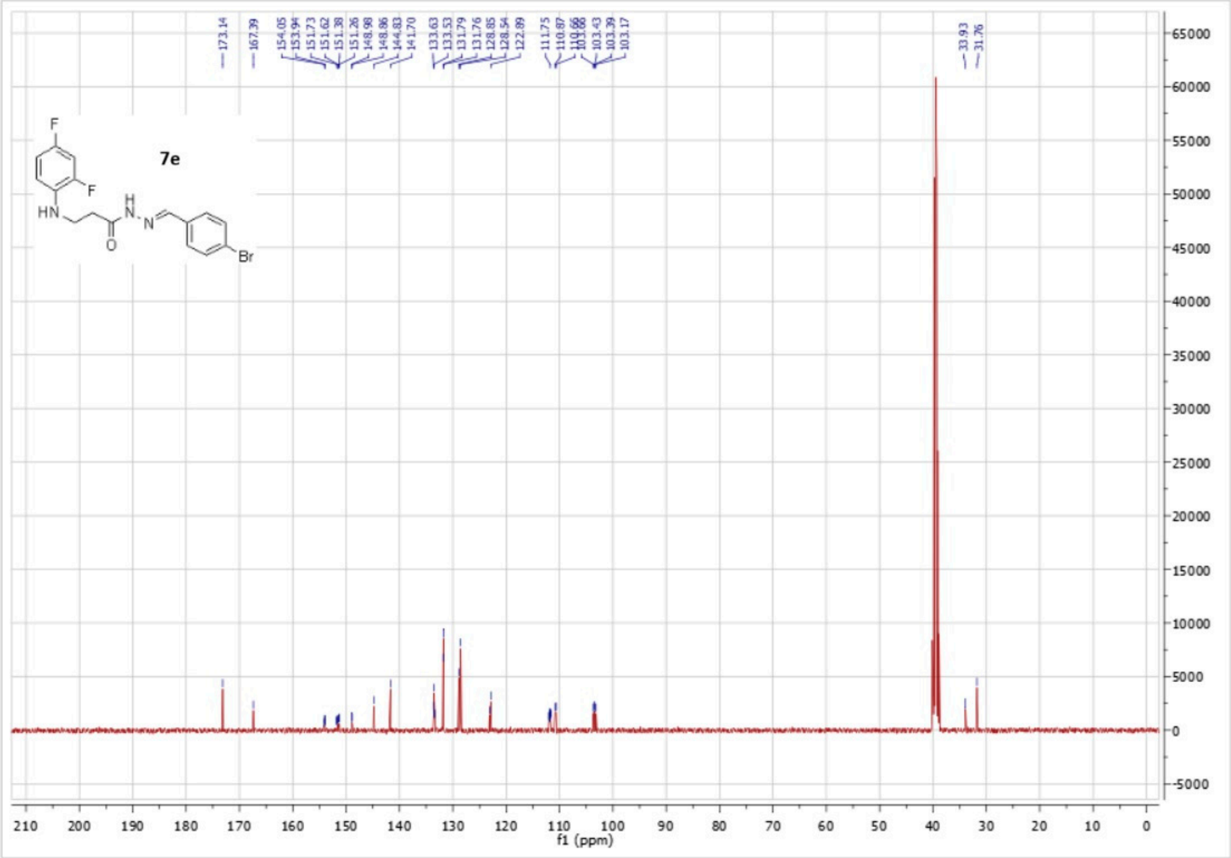

*3-[(2,4-Difluorophenyl)amino]-N'-(4-methylbenzylidene)propanehydrazide (7**f**)*

Figure S29. ^{1}H NMR spectrum of compound **7f.**

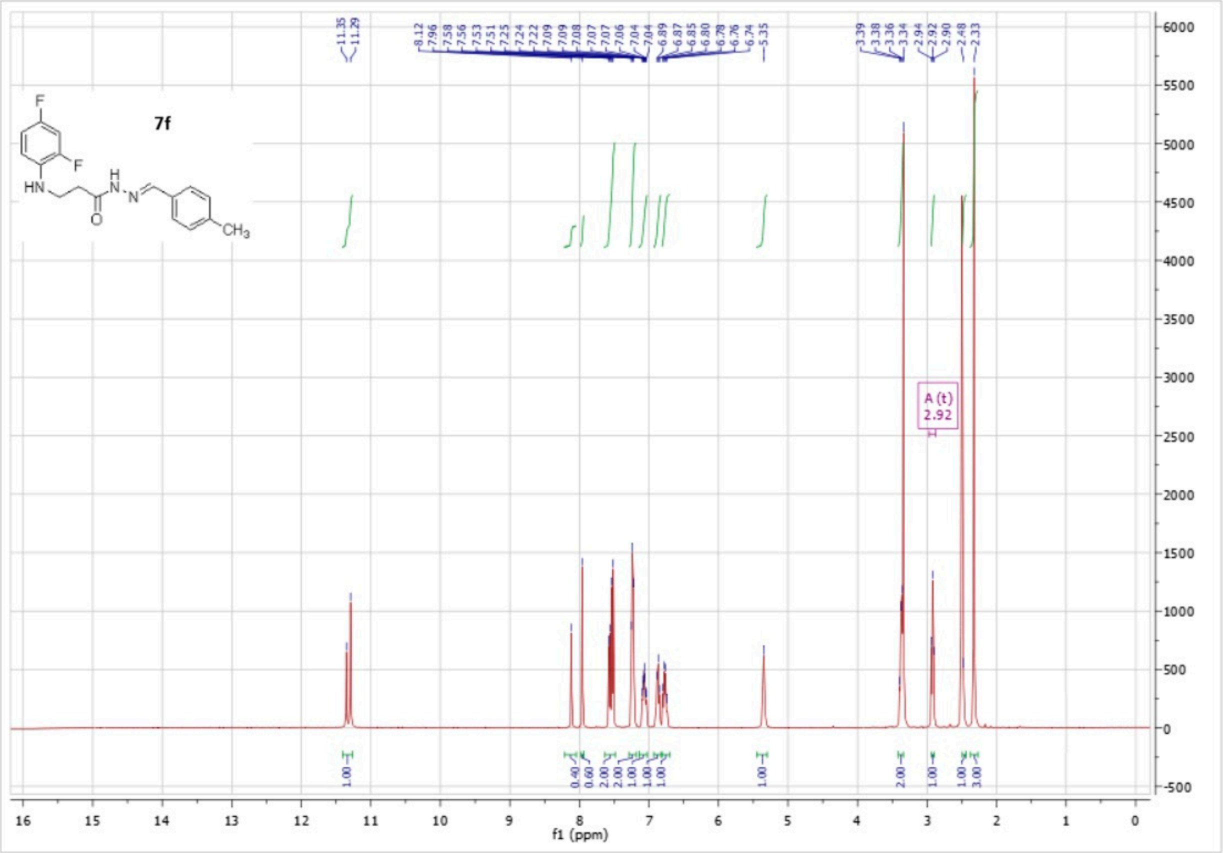

Figure S30. ^{13}C NMR spectrum of compound **7f.**

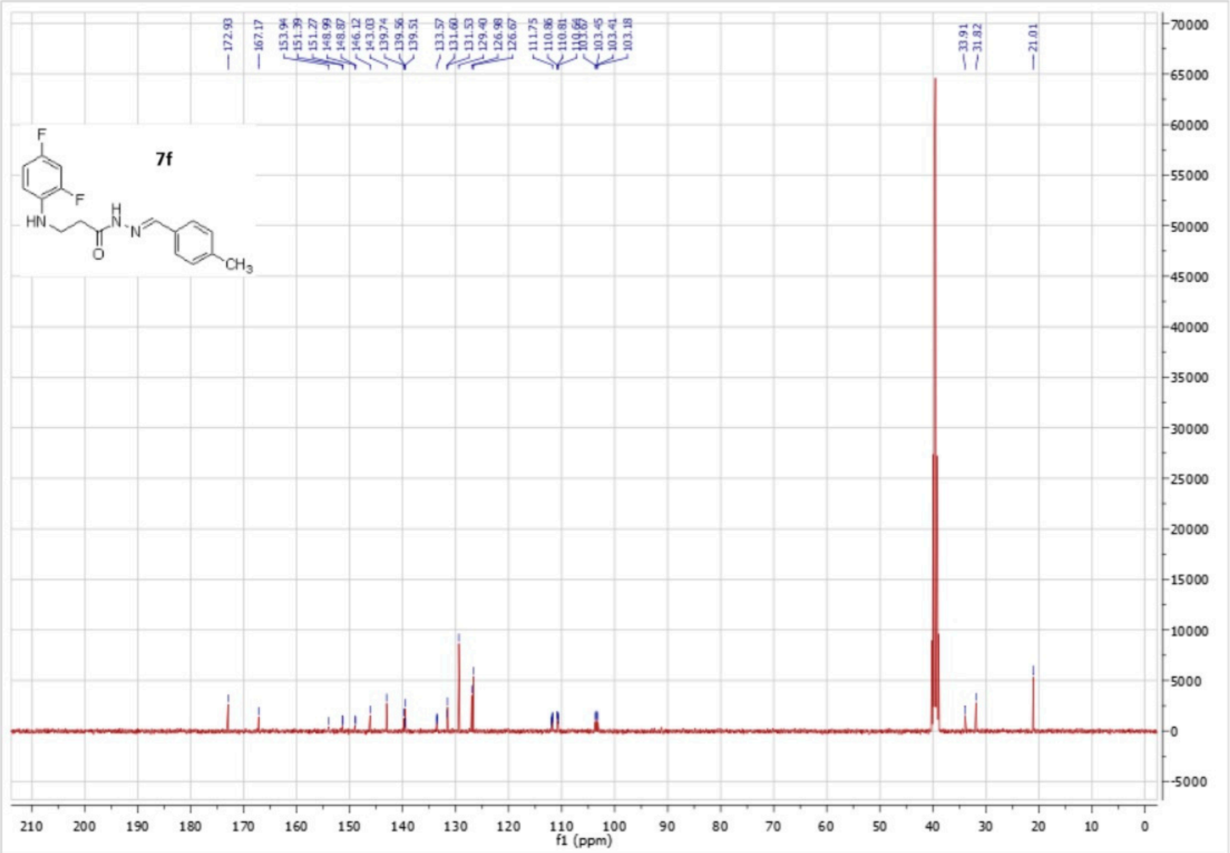

*3-[(2,4-Difluorophenyl)amino]-N'-(4-methoxybenzylidene)propanehydrazide (**7g**)*

Figure S31. ^{1}H NMR spectrum of compound **7g.**

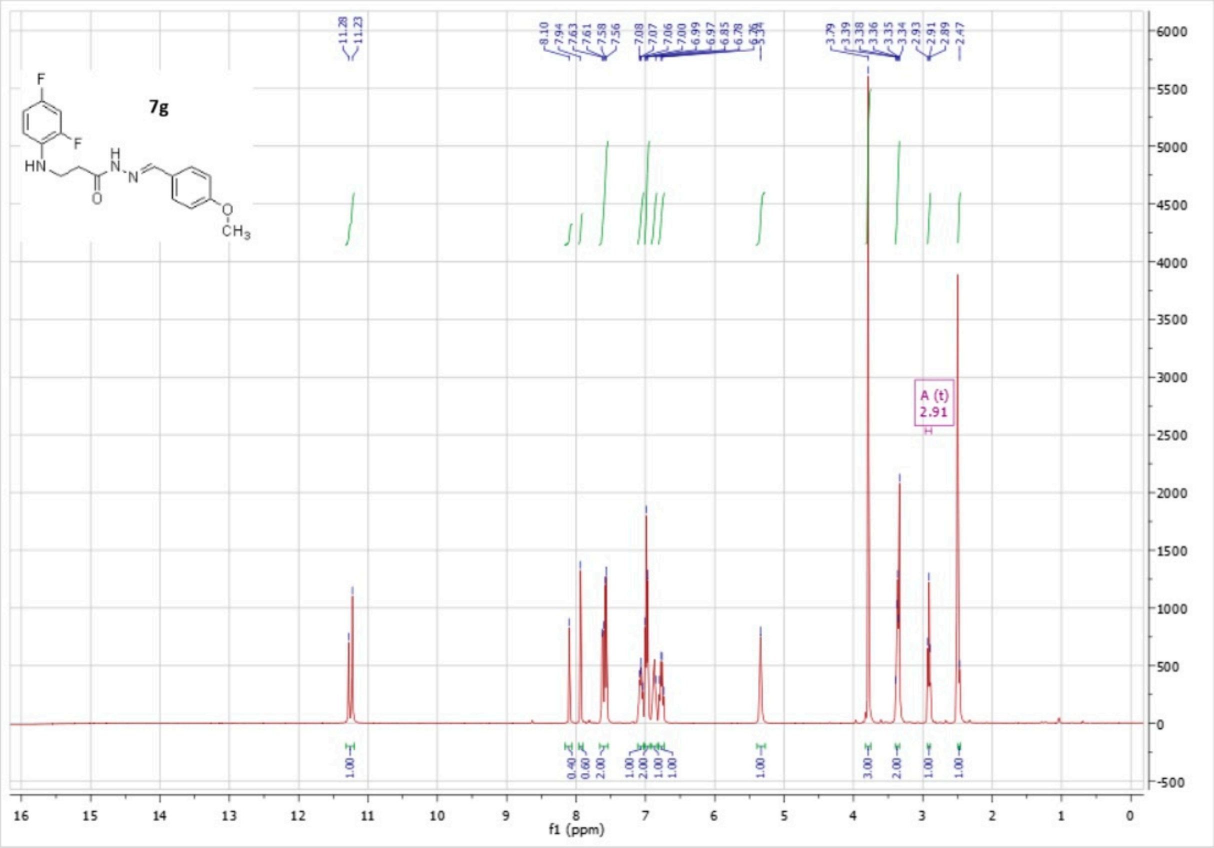

Figure S32. ^{13}C NMR spectrum of compound **7g.**

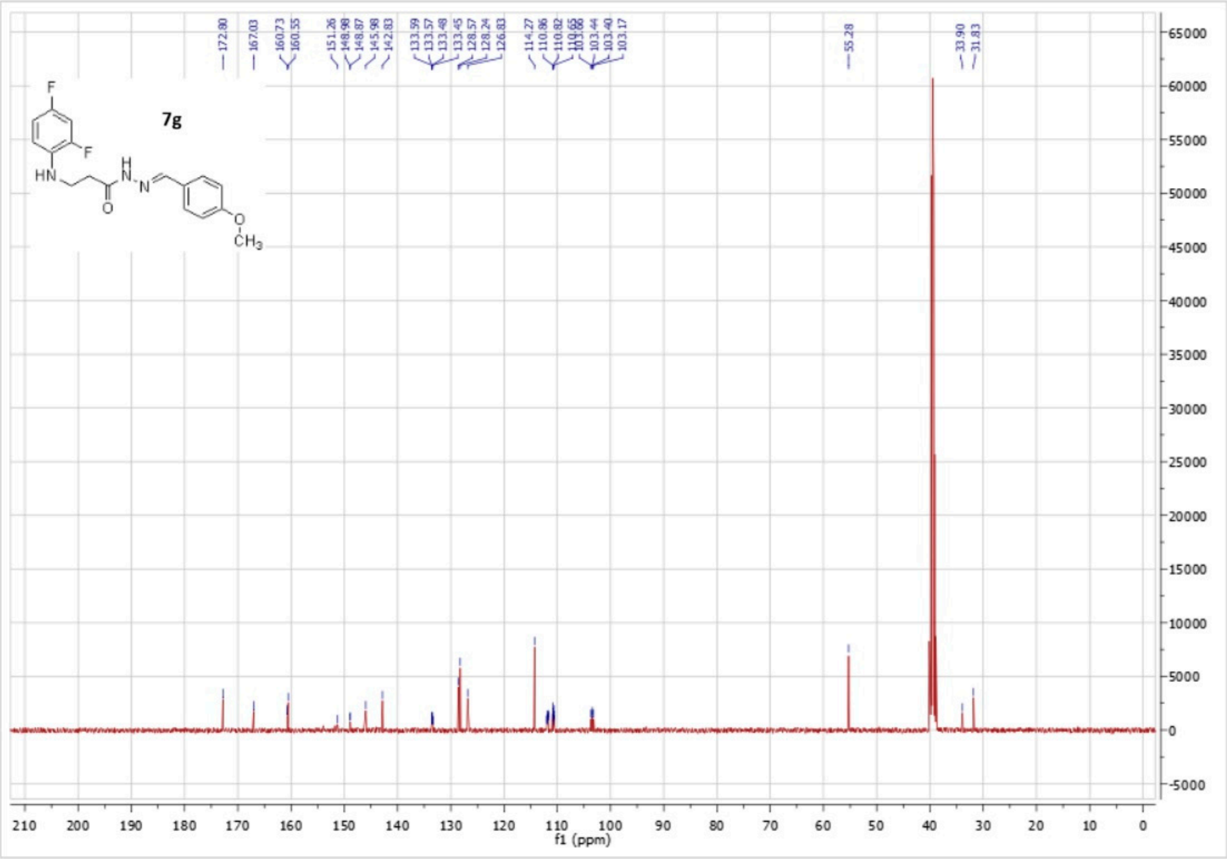

*3-[(2,4-Difluorophenyl)amino]-N'-(3,4,5-trimethoxybenzylidene)propanehydrazide (7**h**)*

Figure S33. ^{1}H NMR spectrum of compound **7h.**

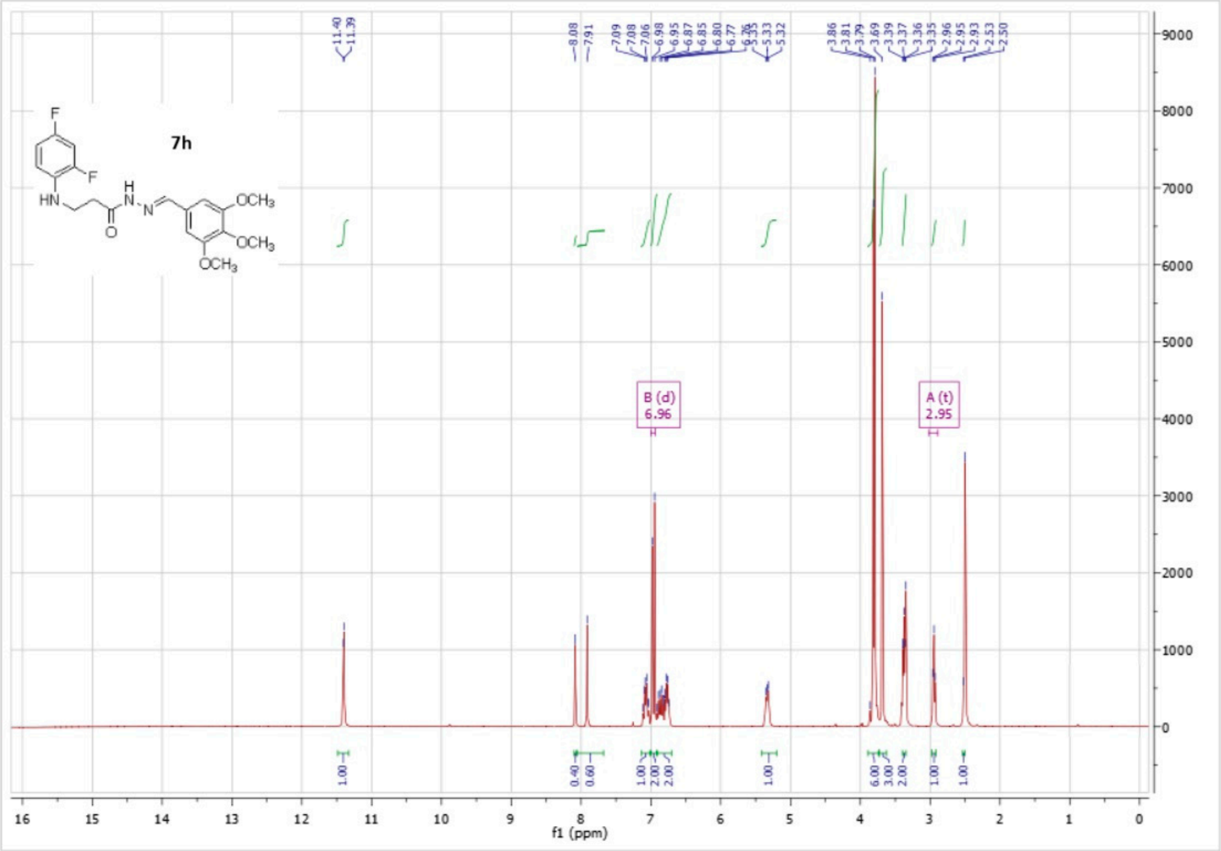

Figure S34. ^{13}C NMR spectrum of compound **7h.**

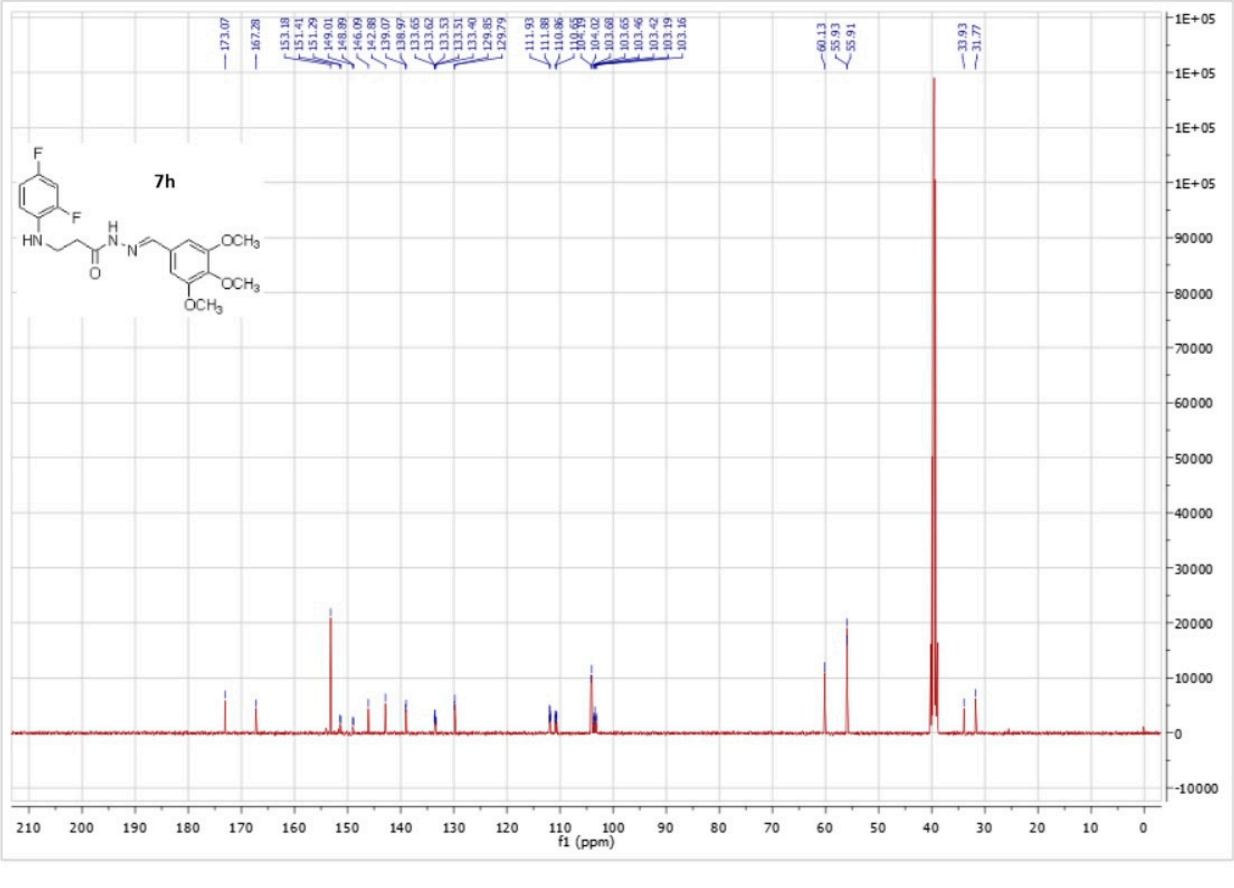

*3-[(2,4-Difluorophenyl)amino]-N'-[4-(dimethylamino)benzylidene]propanehydrazide (7**i**)*

Figure S35. ^{1}H NMR spectrum of compound **7i.**

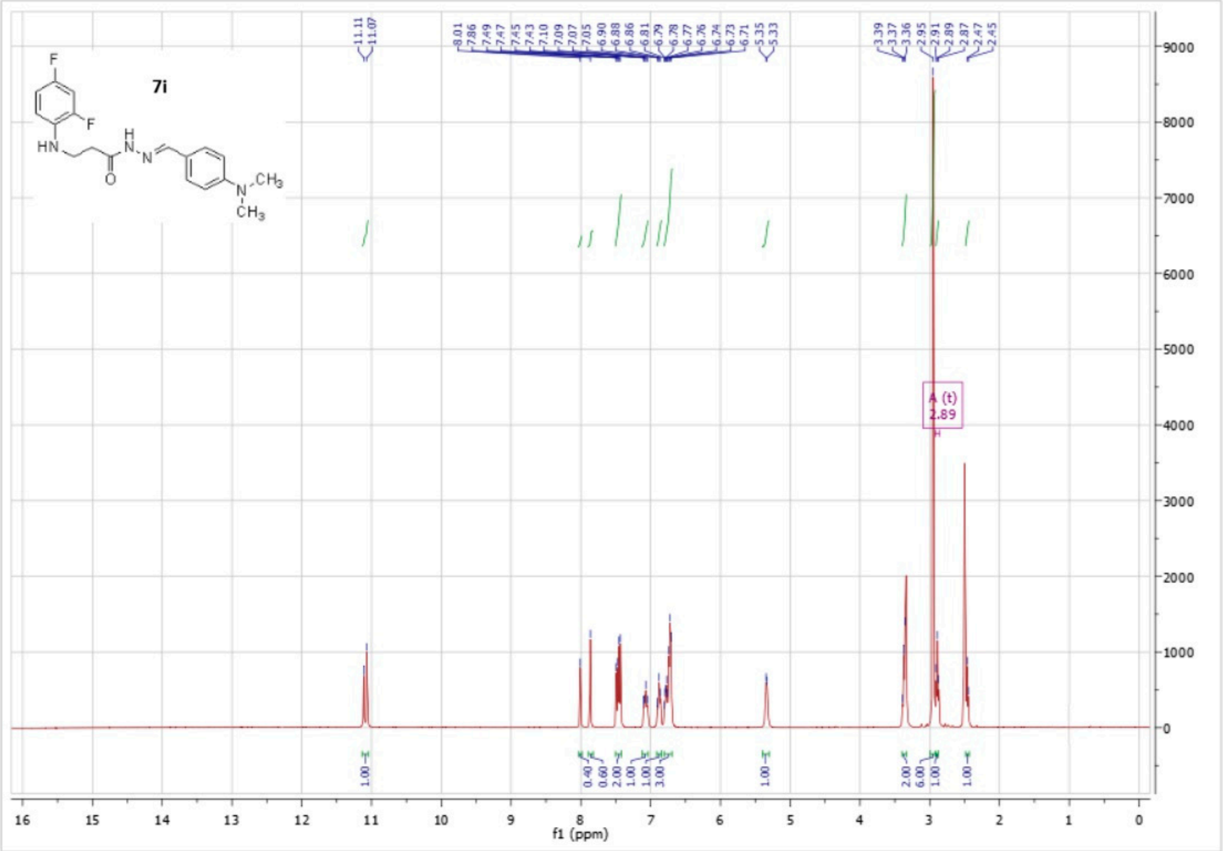

Figure S36. ^{13}C NMR spectrum of compound **7i.**

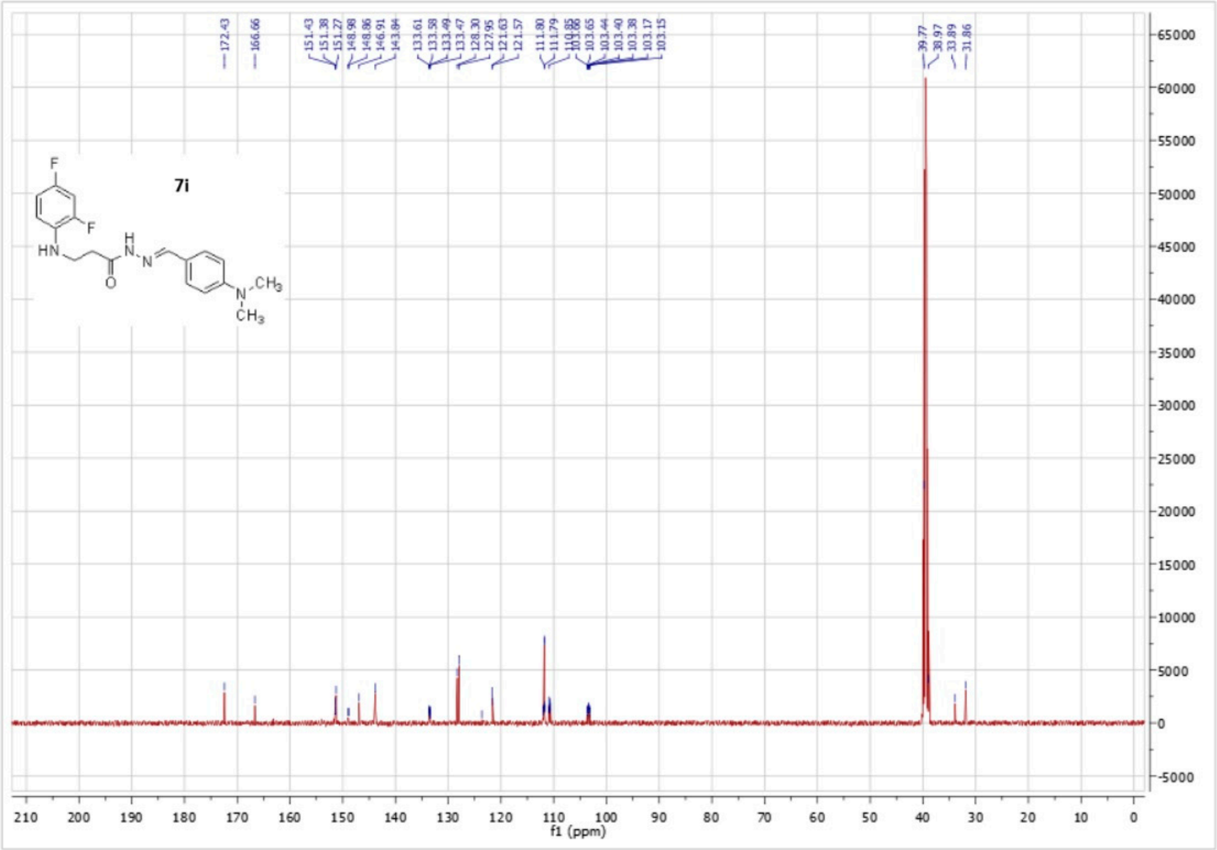

*3-[(2,4-Difluorophenyl)amino]-N'-(4-nitrobenzylidene)-propanehydrazide (7**j**)*

Figure S37. ^{1}H NMR spectrum of compound **7j.**

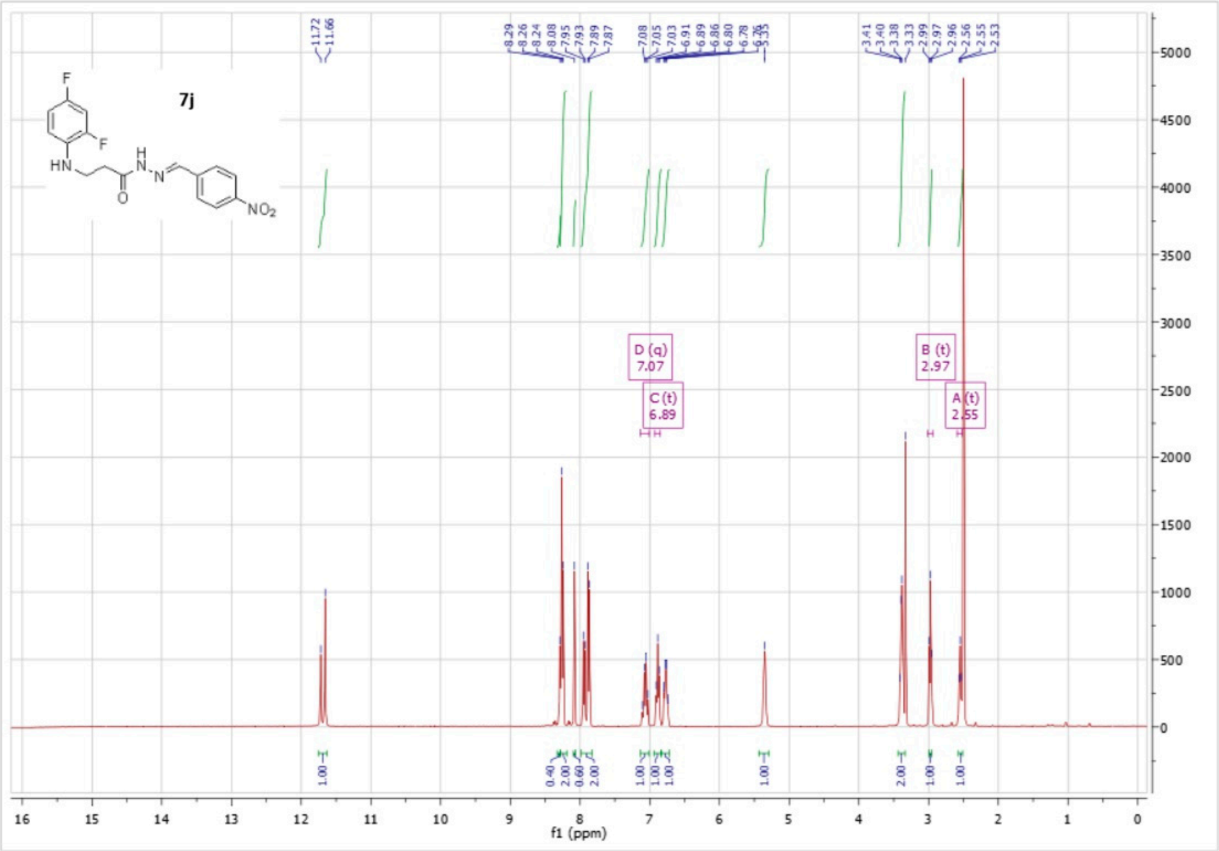

Figure S38. ^{13}C NMR spectrum of compound **7j.**

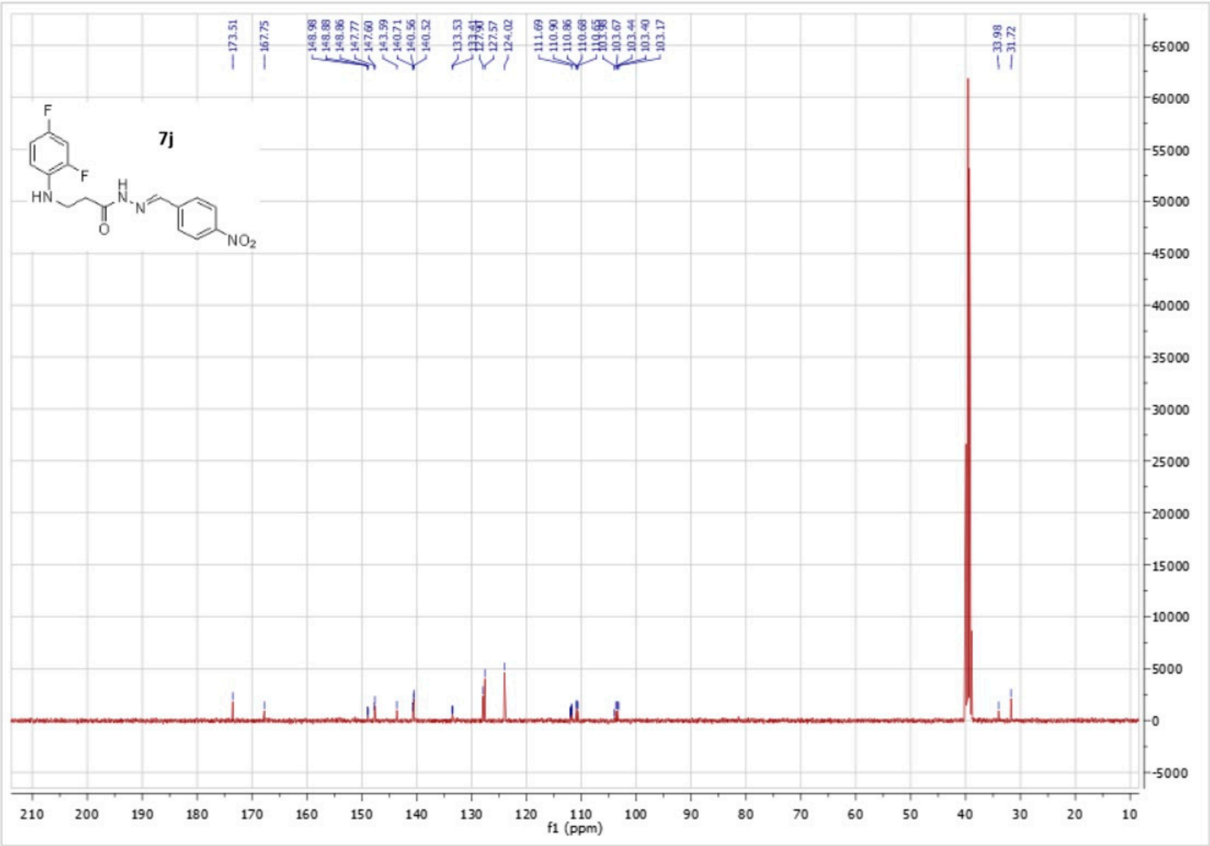

*N'-(2-Chloro-4-nitrobenzylidene)-3-[(2,4-difluorophenyl)amino]propanehydrazide (7**k**)*

Figure S39. ^{1}H NMR spectrum of compound **7k.**

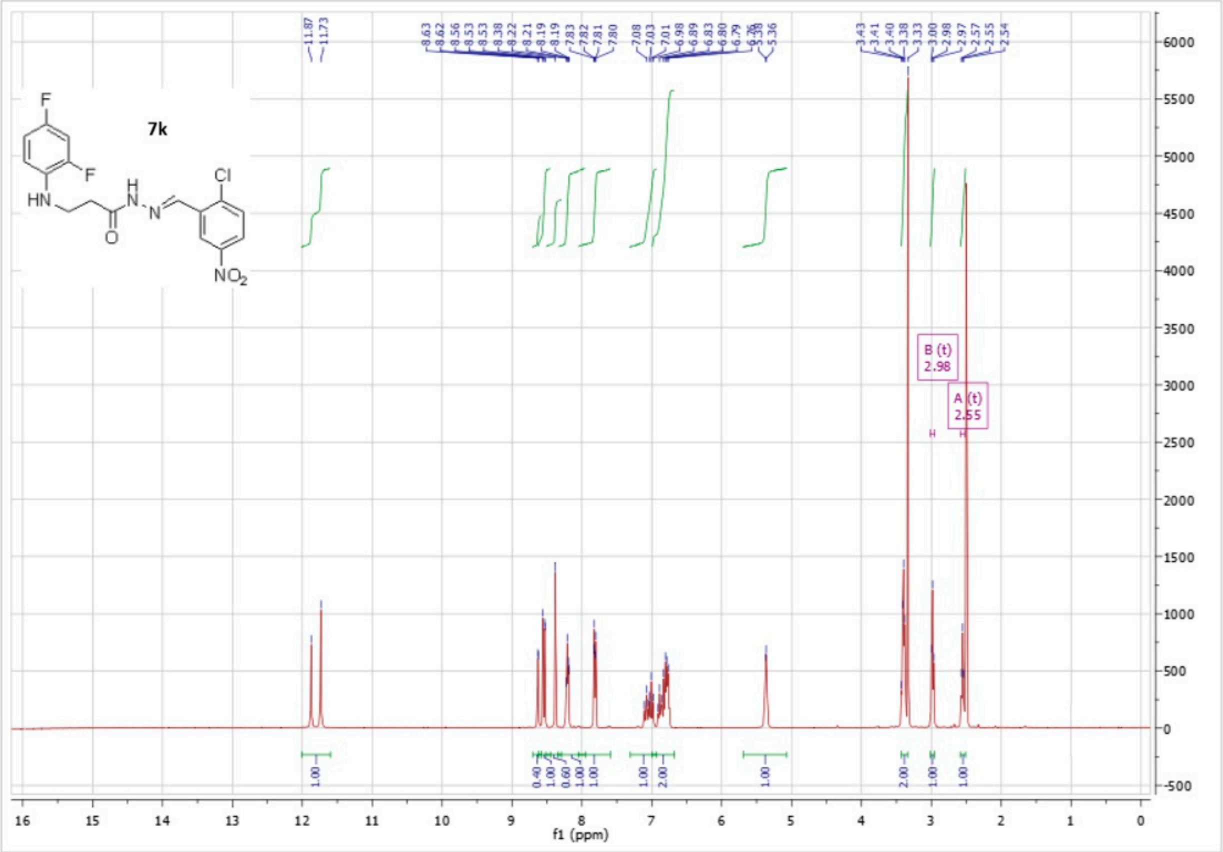

Figure S40. ^{13}C NMR spectrum of compound **7k.**

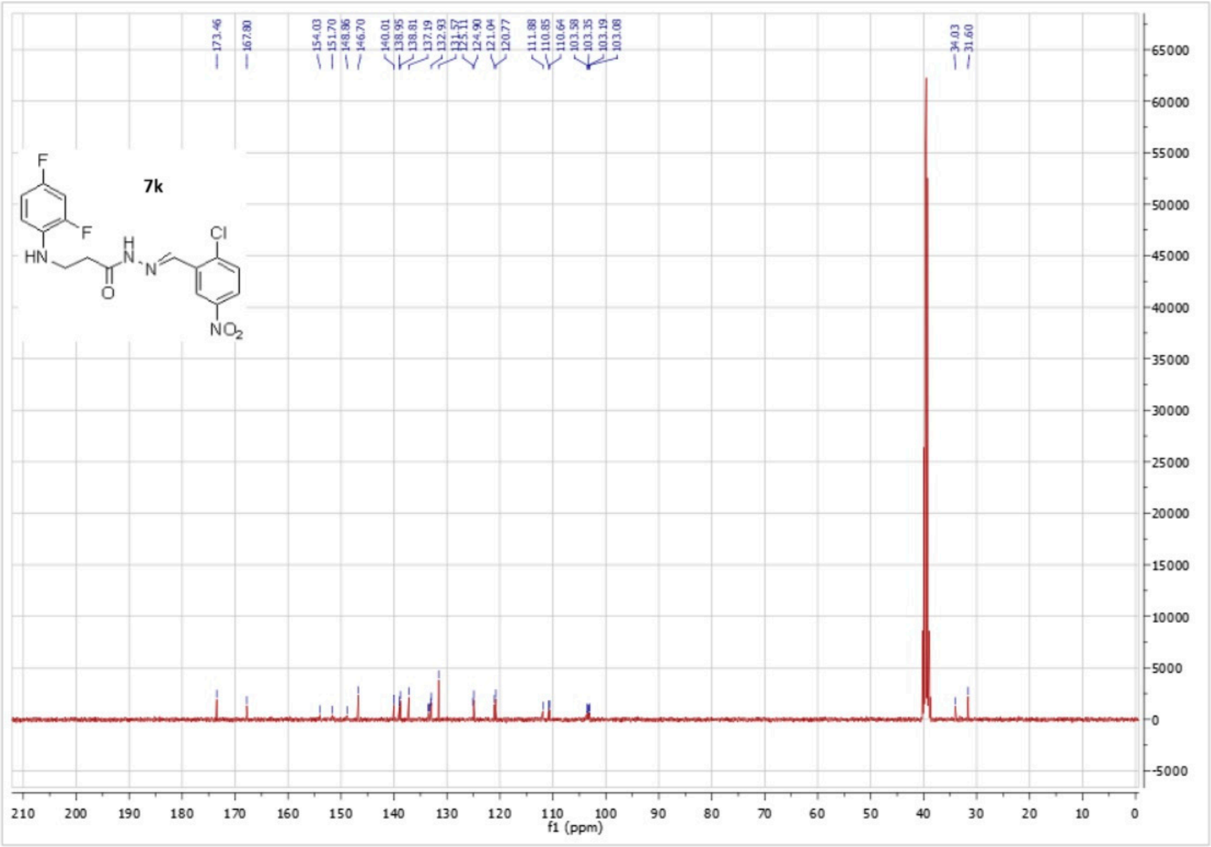

*3-[(2,4-Difluorophenyl)amino]-N'-(naphthalen-1-ylmethylene)propanehydrazide (**8**)*

Figure S41. ^{1}H NMR spectrum of compound **8.**

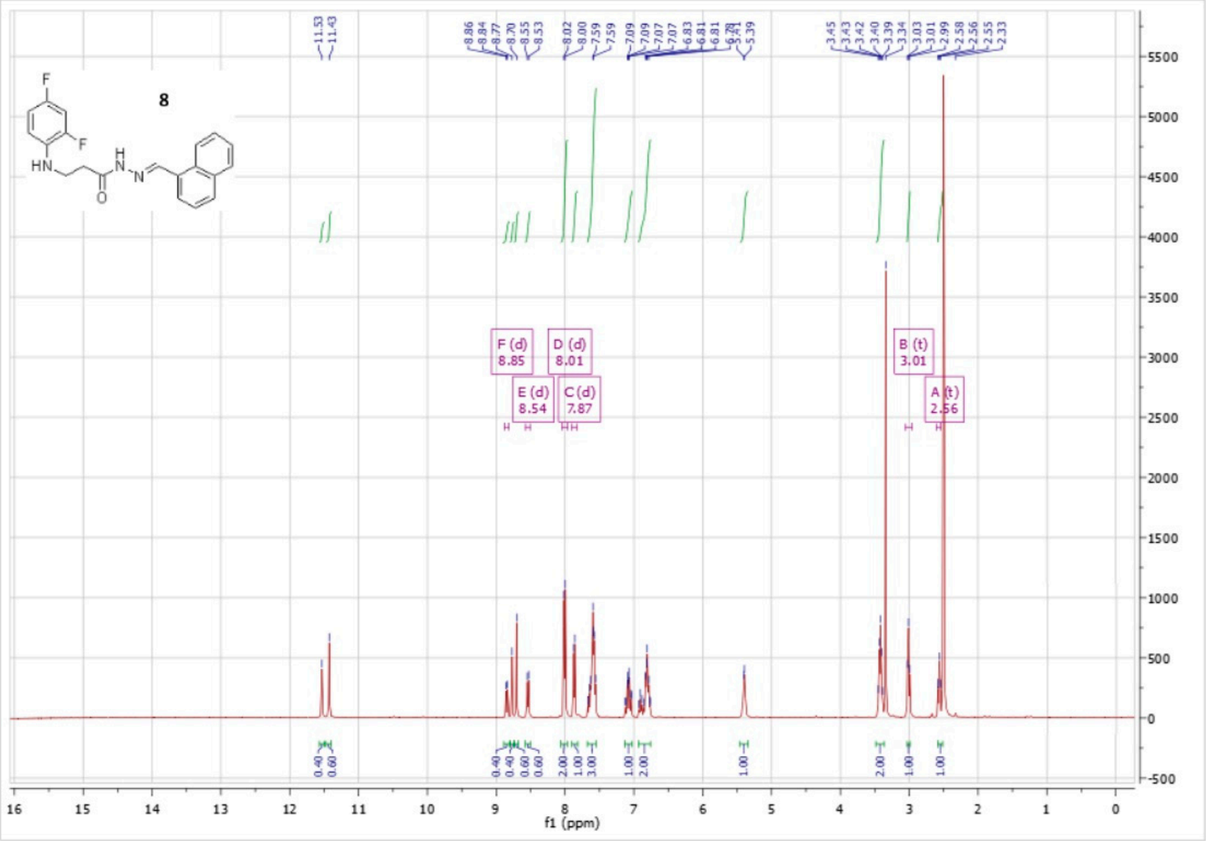

Figure S42. ^{13}C NMR spectrum of compound **8.**

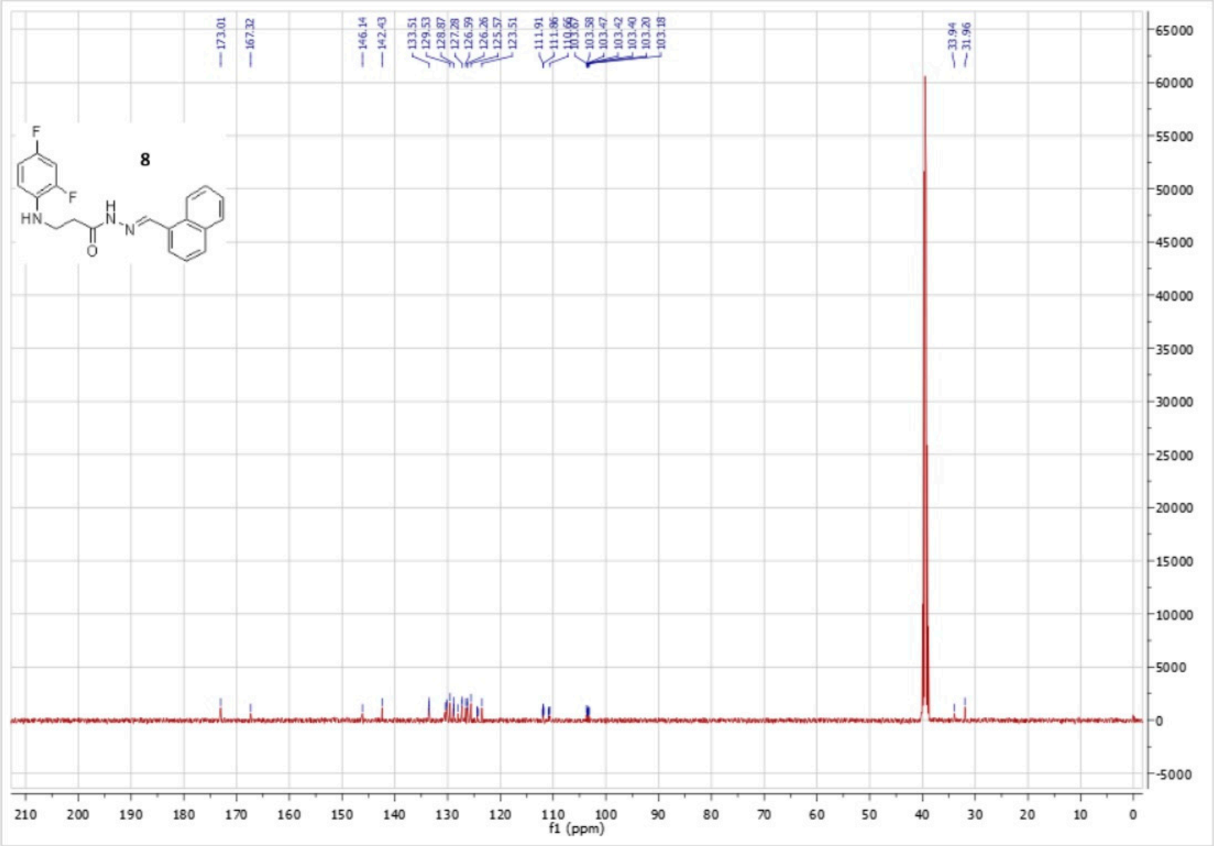

*3-[(2,4-Difluorophenyl)amino]-N'-(naphthalen-1-ylmethylene)propanehydrazide (**9**)*

Figure S43. ^{1}H NMR spectrum of compound **9.**

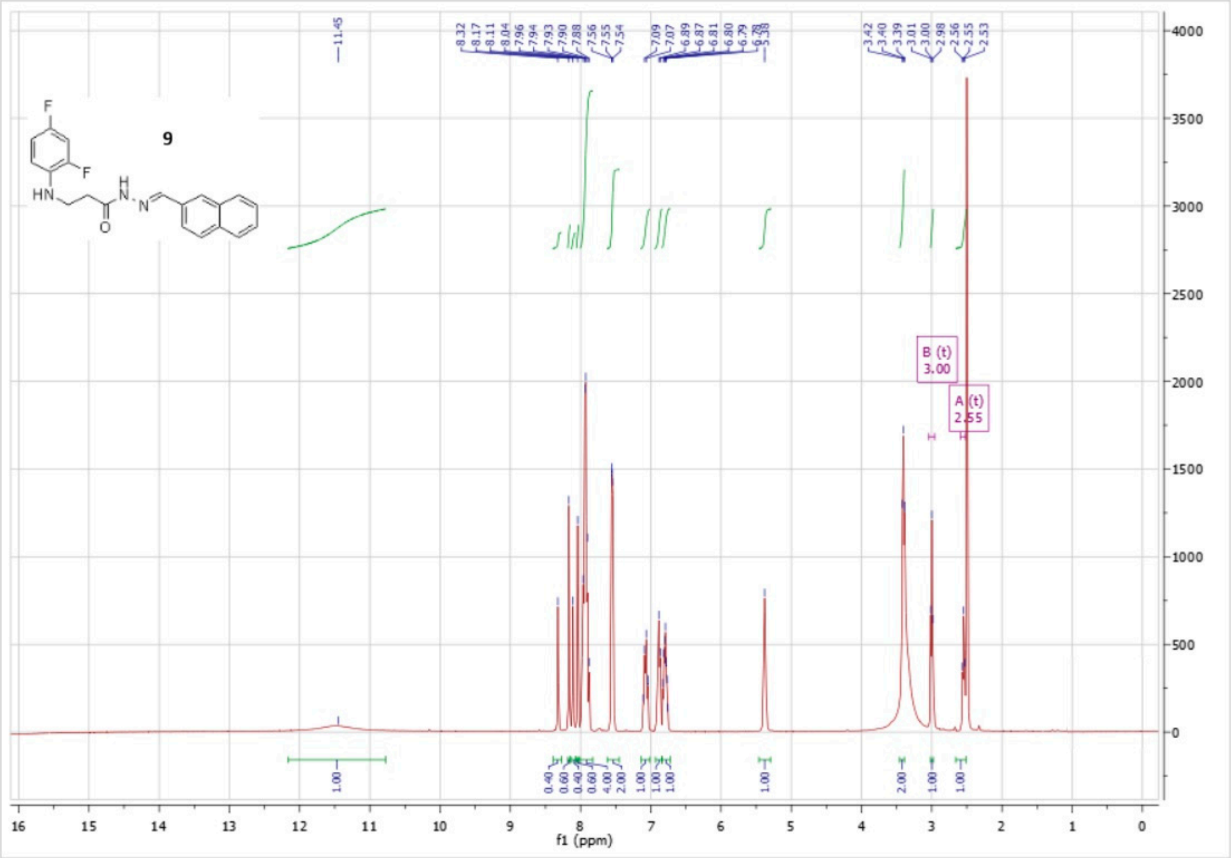

Figure S44. ^{13}C NMR spectrum of compound **9.**

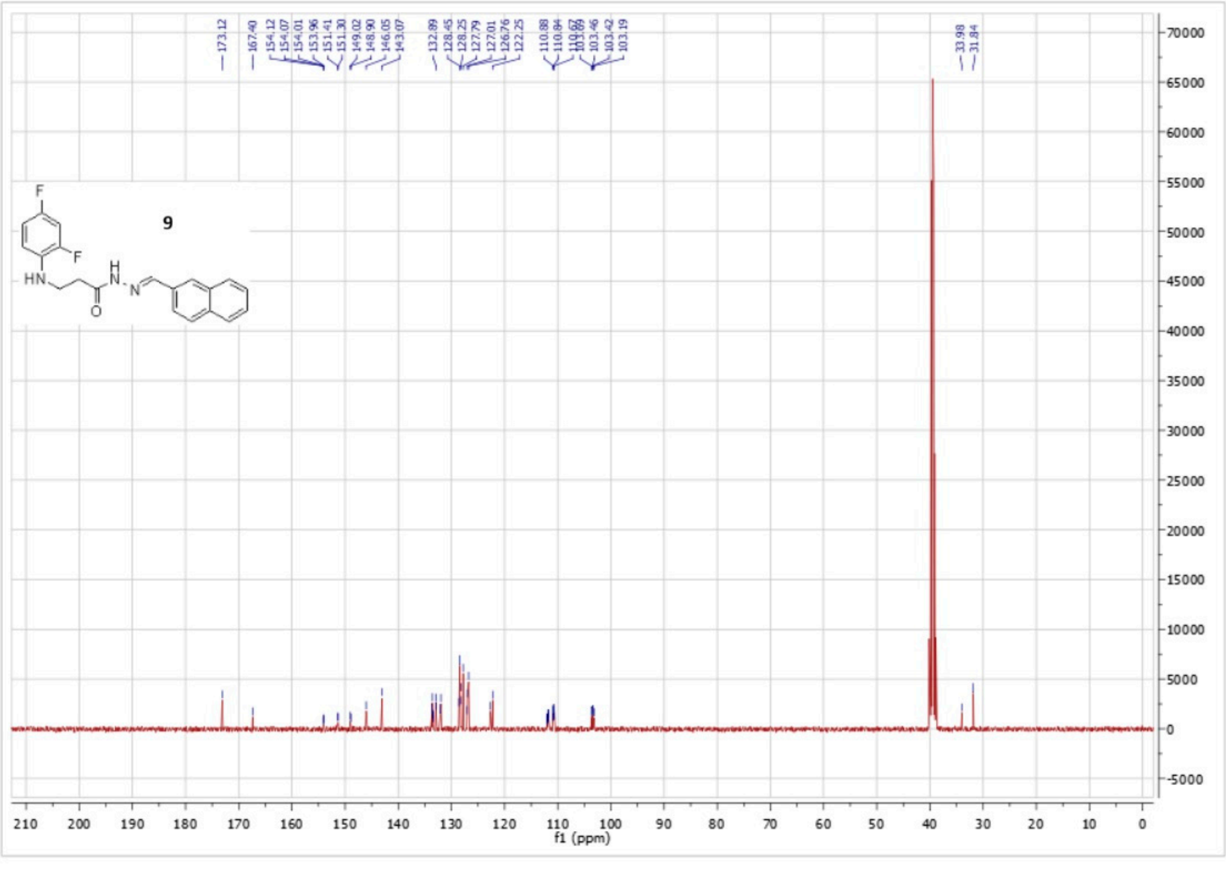

*N'-Benzylidene-3-[(2,4-difluorophenyl)amino]-N-ethylpropanehydrazide (**10**)*

Figure S45. ^{1}H NMR spectrum of compound **10.**

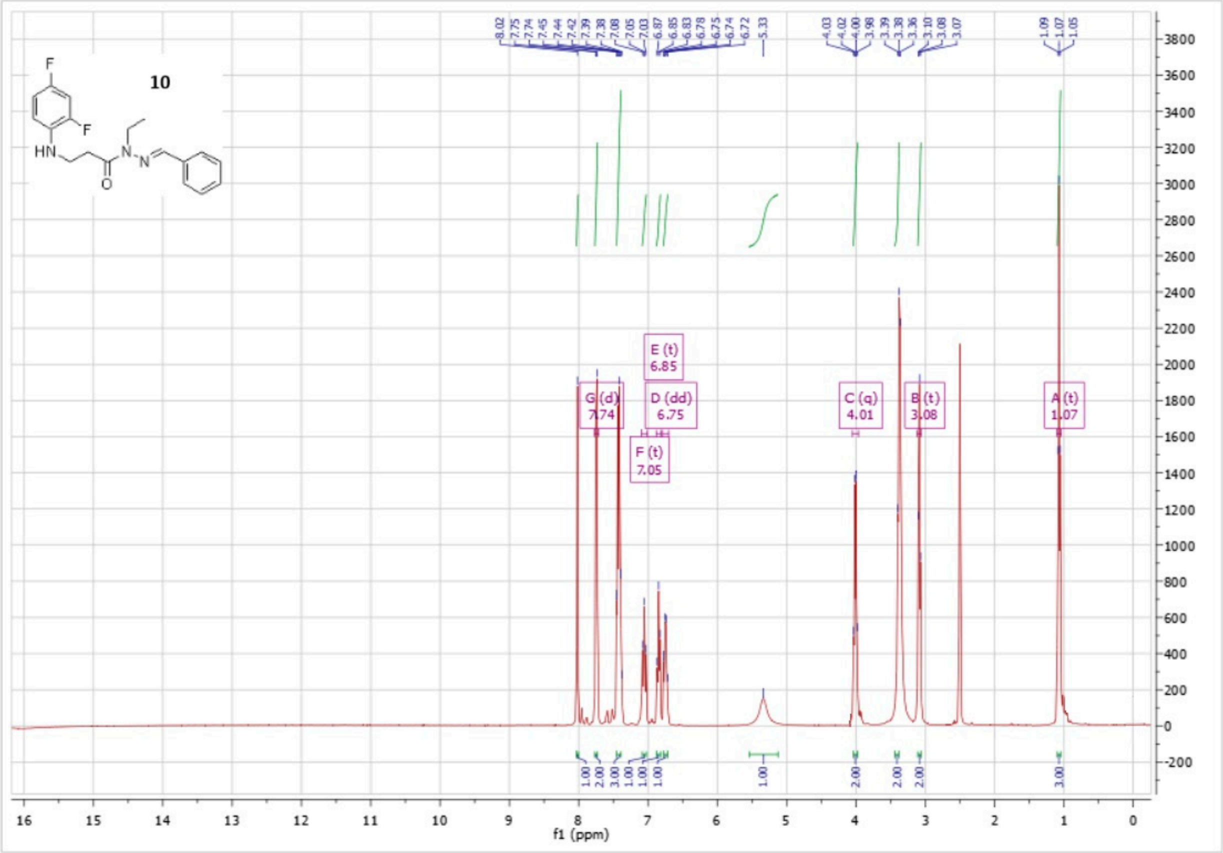

Figure S46. ^{13}C NMR spectrum of compound **10.**

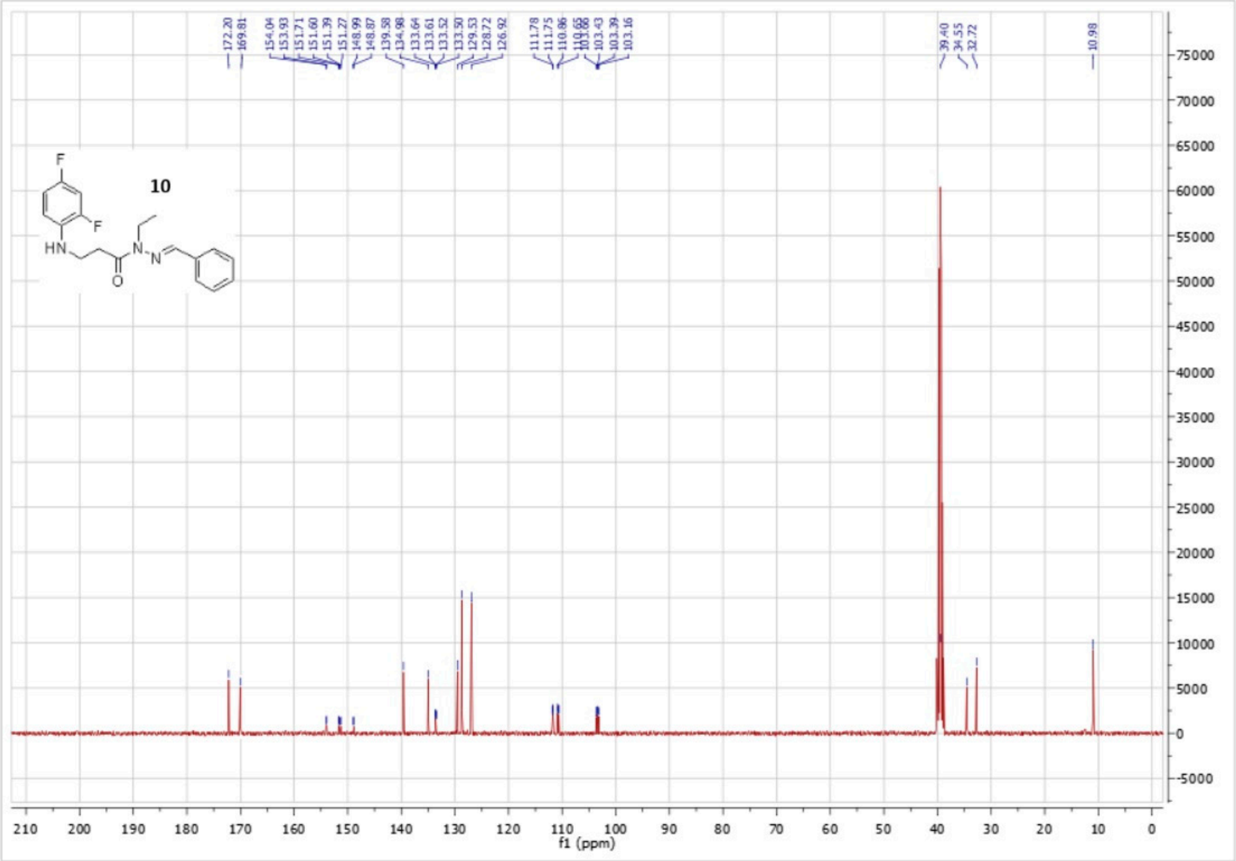

*N'-(4-Chlorobenzylidene)-3-[(2,4-difluorophenyl)amino]-N-ethylpropanehydrazide (**11**)*

Figure S47. ^{1}H NMR spectrum of compound **11.**

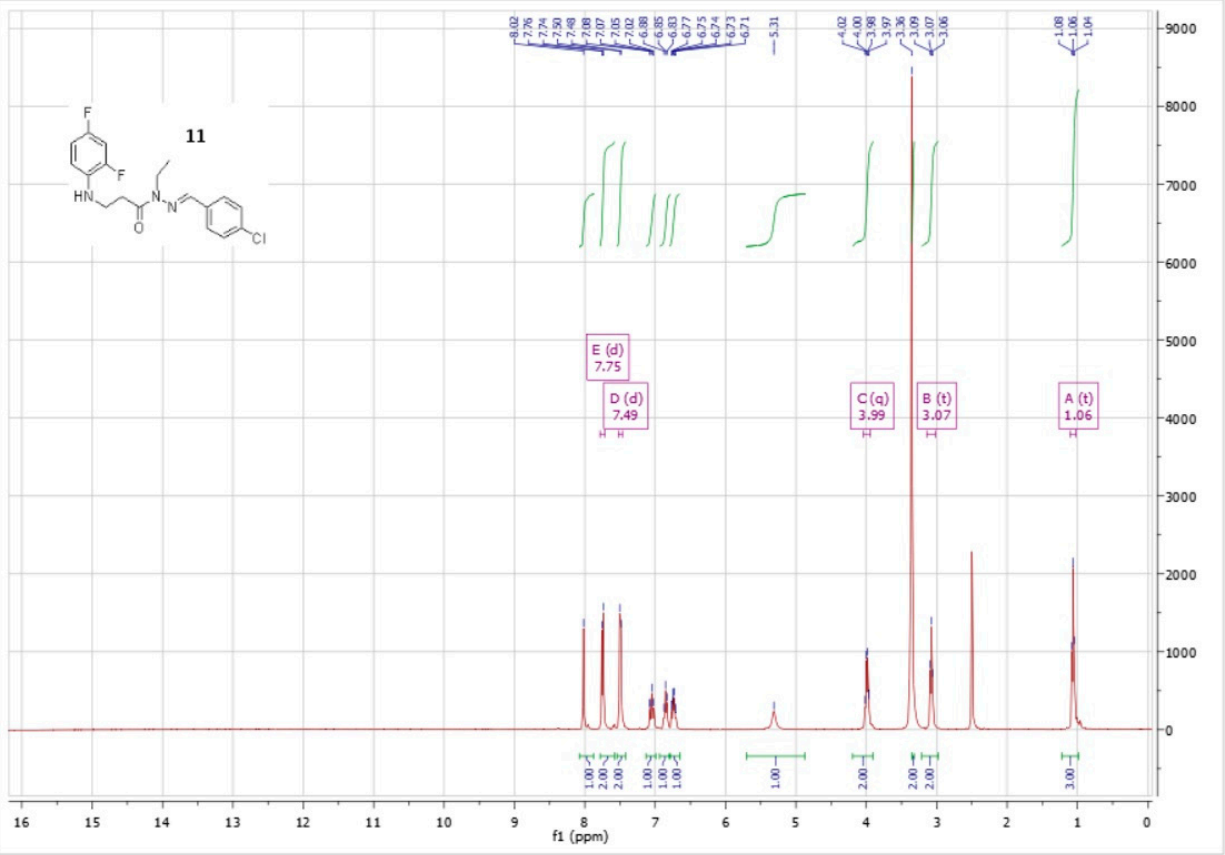

Figure S48. ^{13}C NMR spectrum of compound **11.**

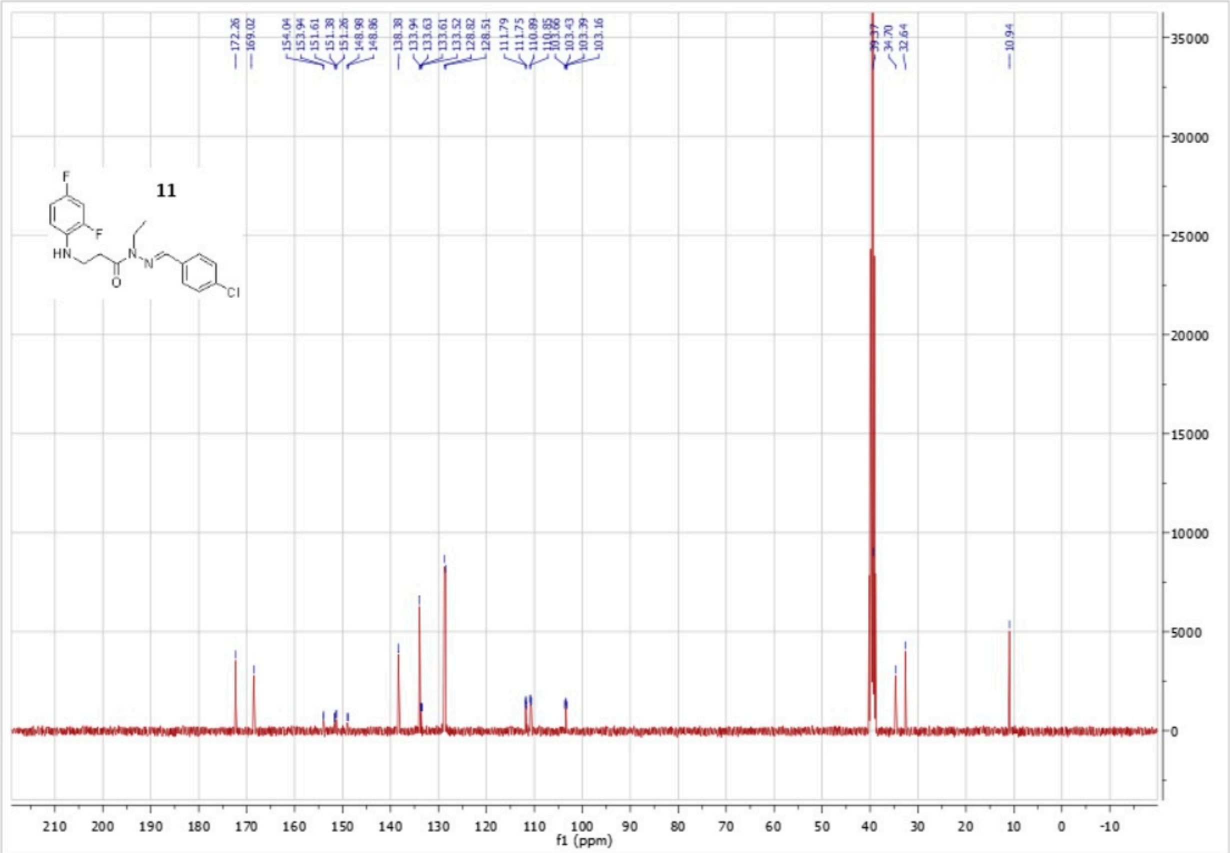

*3-[(2,4-Difluorophenyl)amino]-N-ethyl-N'-(4-methylbenzylidene)propanehydrazide (**12**)*

Figure S49. ^{1}H NMR spectrum of compound **12.**

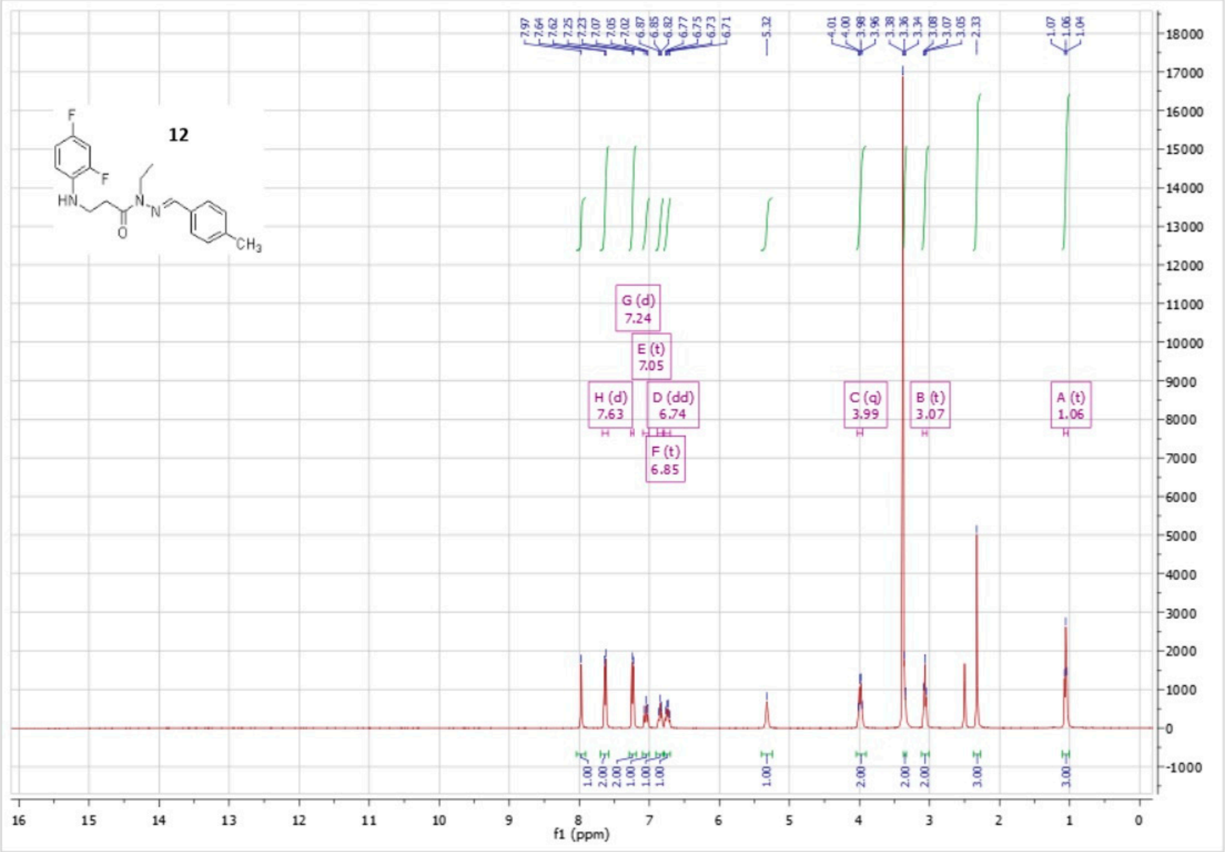

Figure S50. ^{13}C NMR spectrum of compound **12**

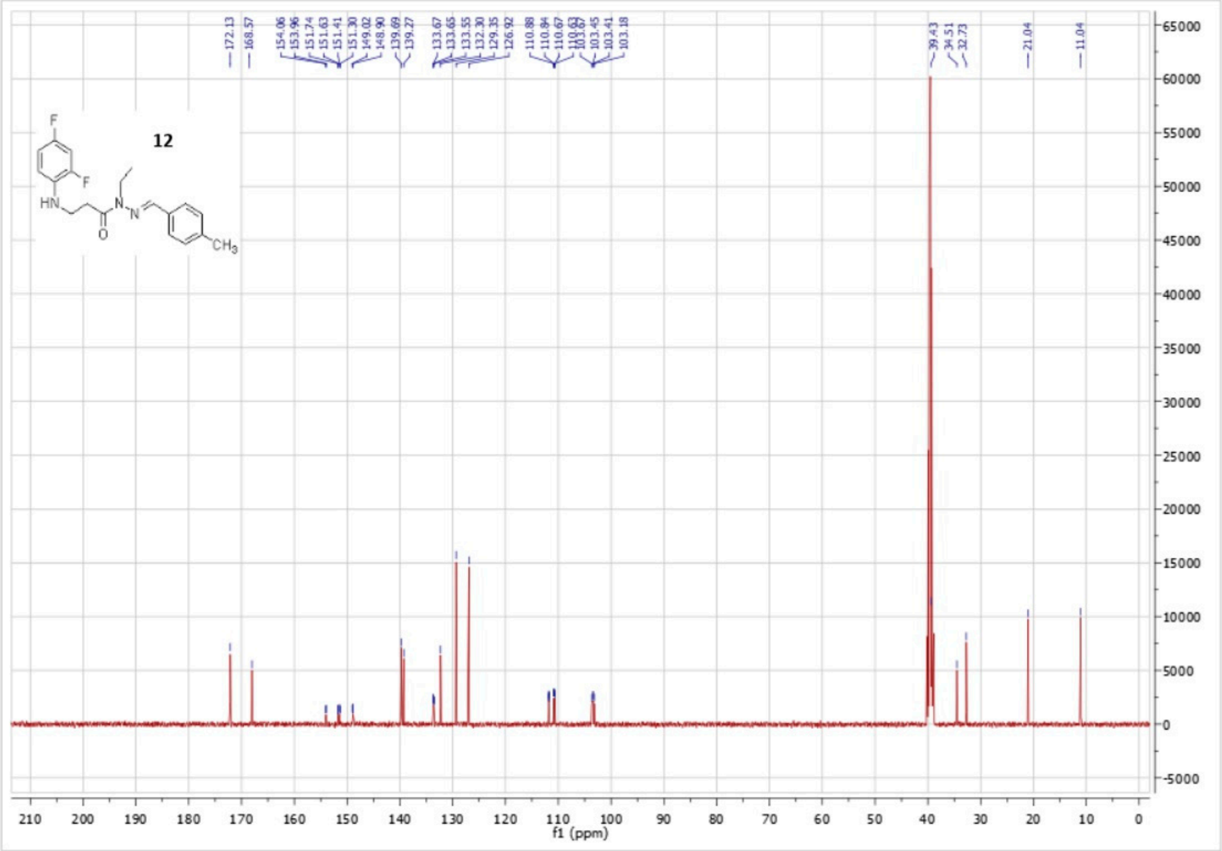

*3-[(2,4-Difluorophenyl)amino]-N'-(propan-2-ylidene)propanehydrazide (**13**)*

Figure S51. ^{1}H NMR spectrum of compound **13.**

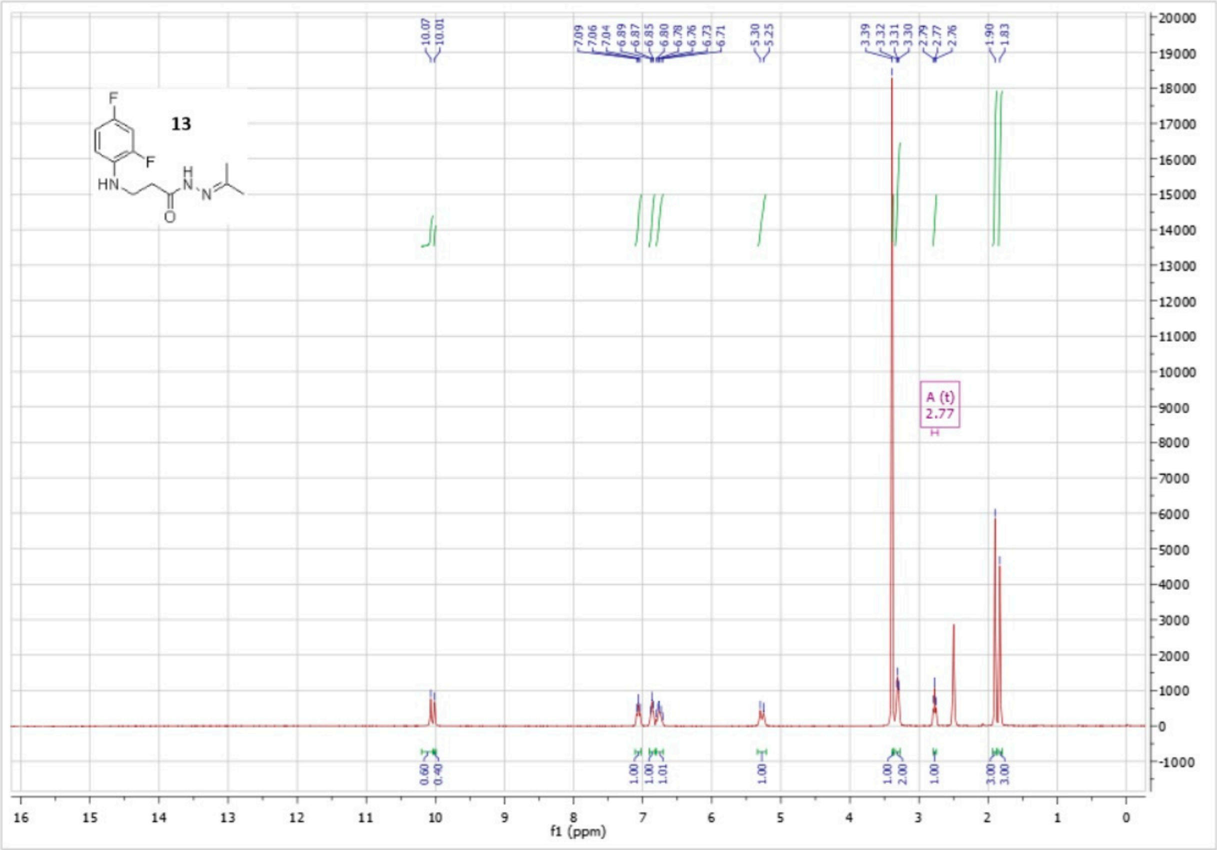

Figure S52. ^{13}C NMR spectrum of compound **13.**

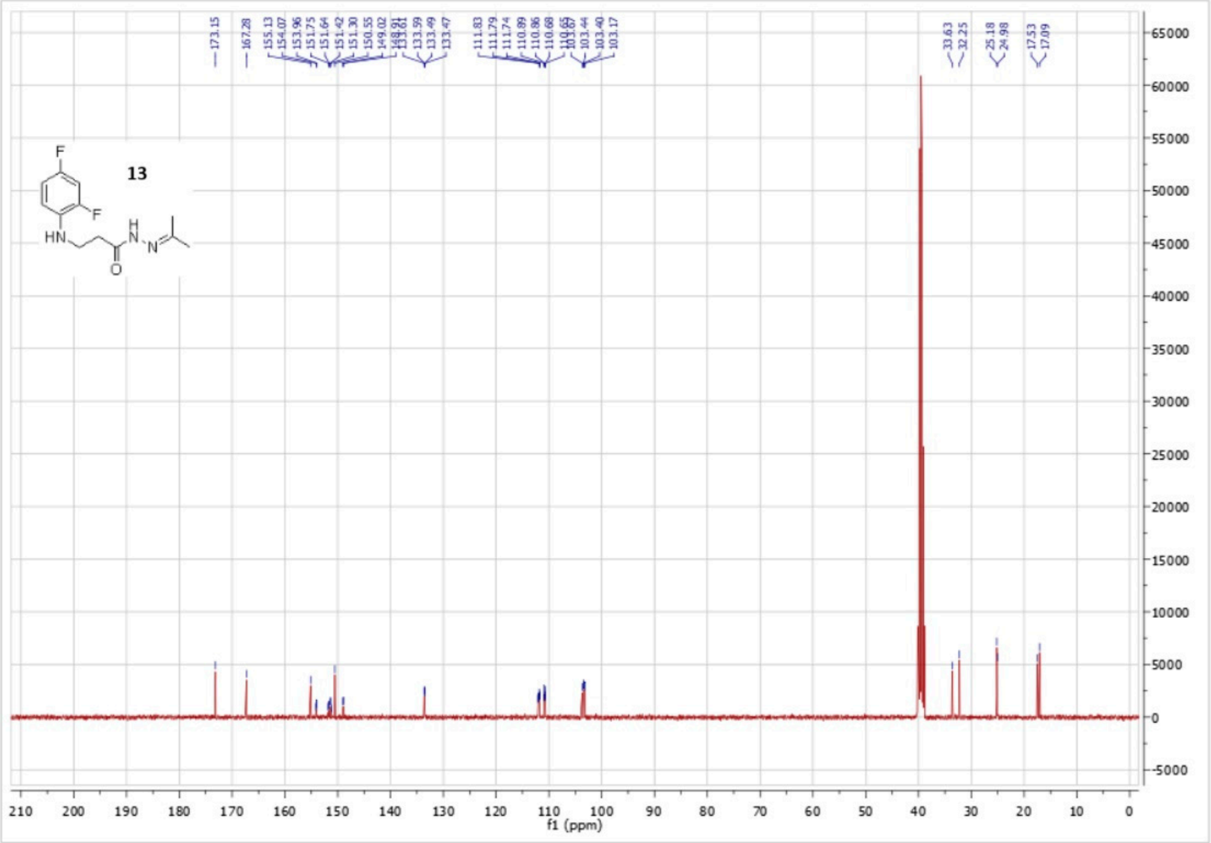

*N'-(Butan-2-ylidene)-3-[(2,4-difluorophenyl)amino]propanehydrazide (**14**)*

Figure S53. ^{1}H NMR spectrum of compound **14.**

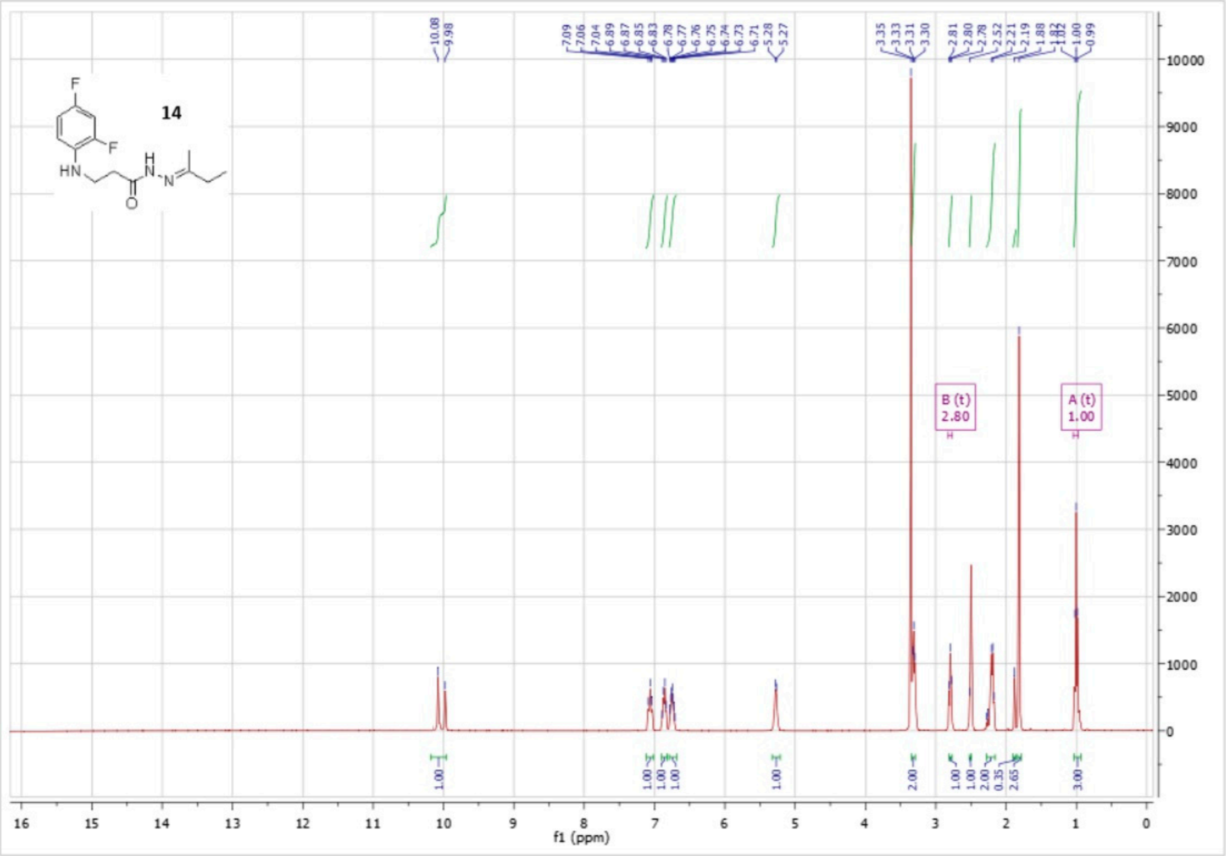

Figure S54. ^{13}C NMR spectrum of compound **14.**

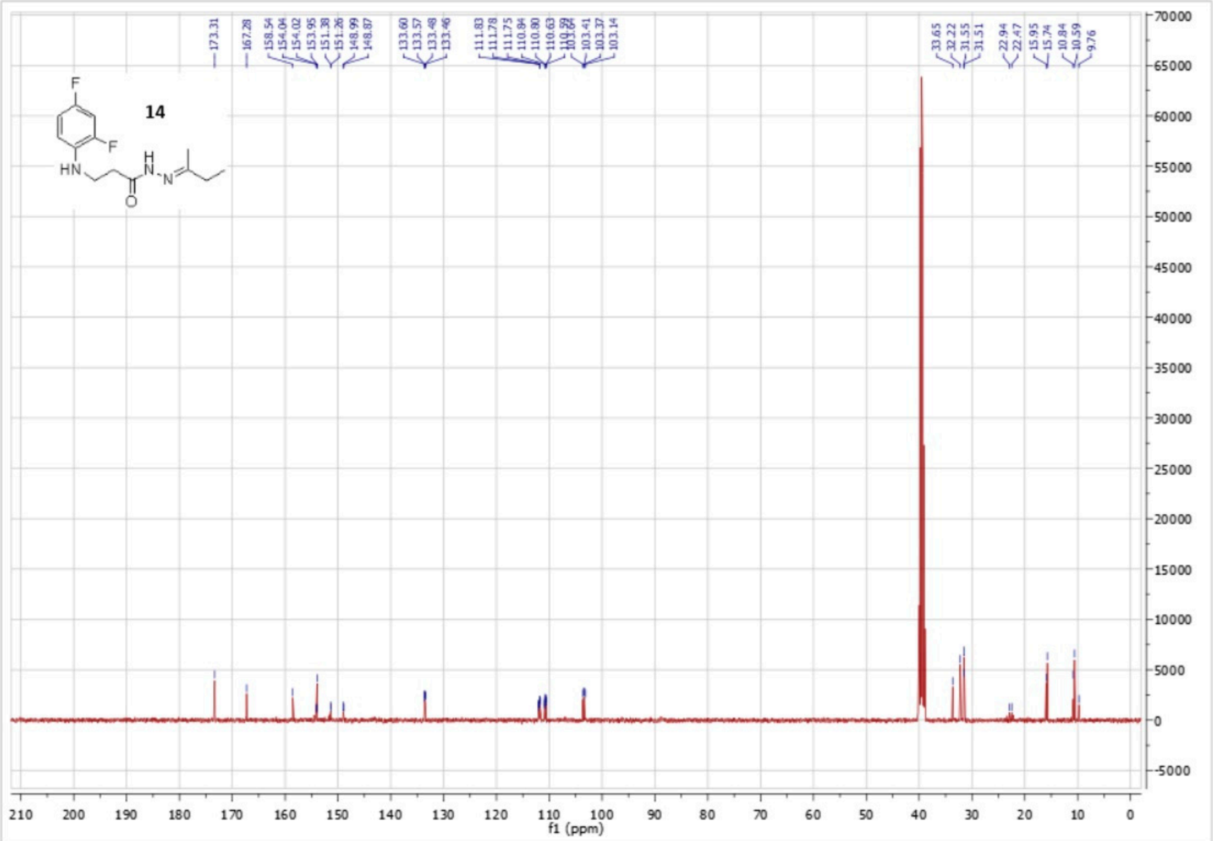

*N'-[1-(4-Aminophenyl)ethylidene]-3-[(2,4-difluorophenyl)amino]propanehydrazide (**15**)*

Figure S55. ^{1}H NMR spectrum of compound **15.**

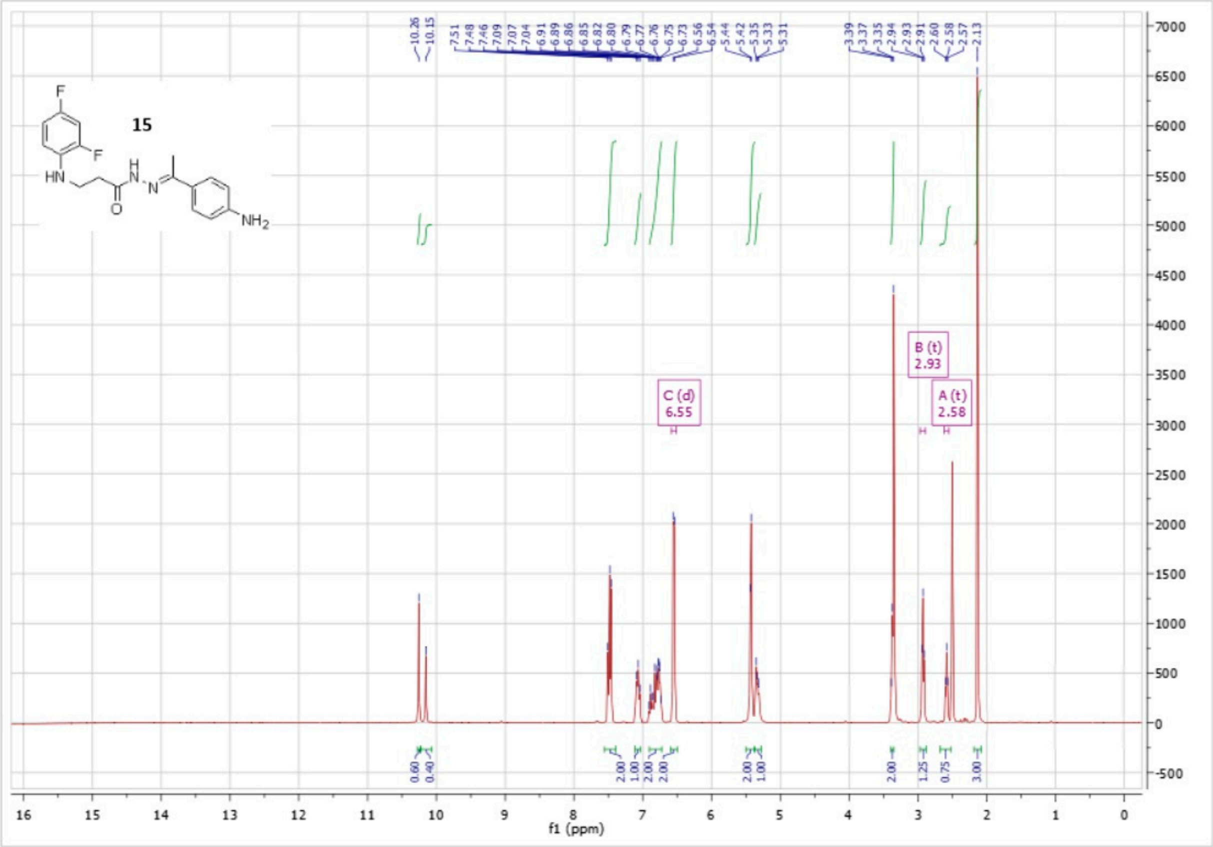

Figure S56. ^{13}C NMR spectrum of compound **15.**

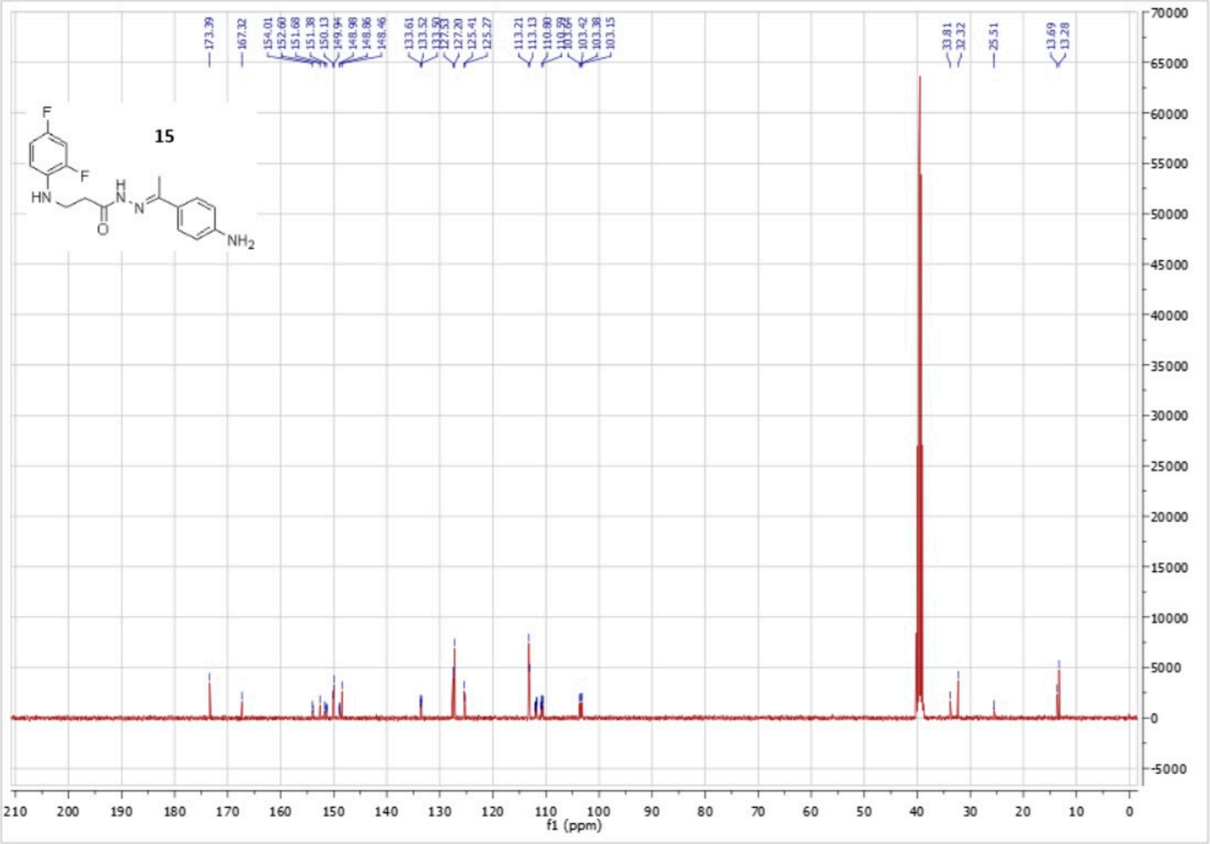

*3-[(2,4-Difluorophenyl)amino]-1-(3,5-dimethyl-1H-pyrazol-1-yl)propan-1-one (**16**)*

Figure S57. ^{1}H NMR spectrum of compound **16.**

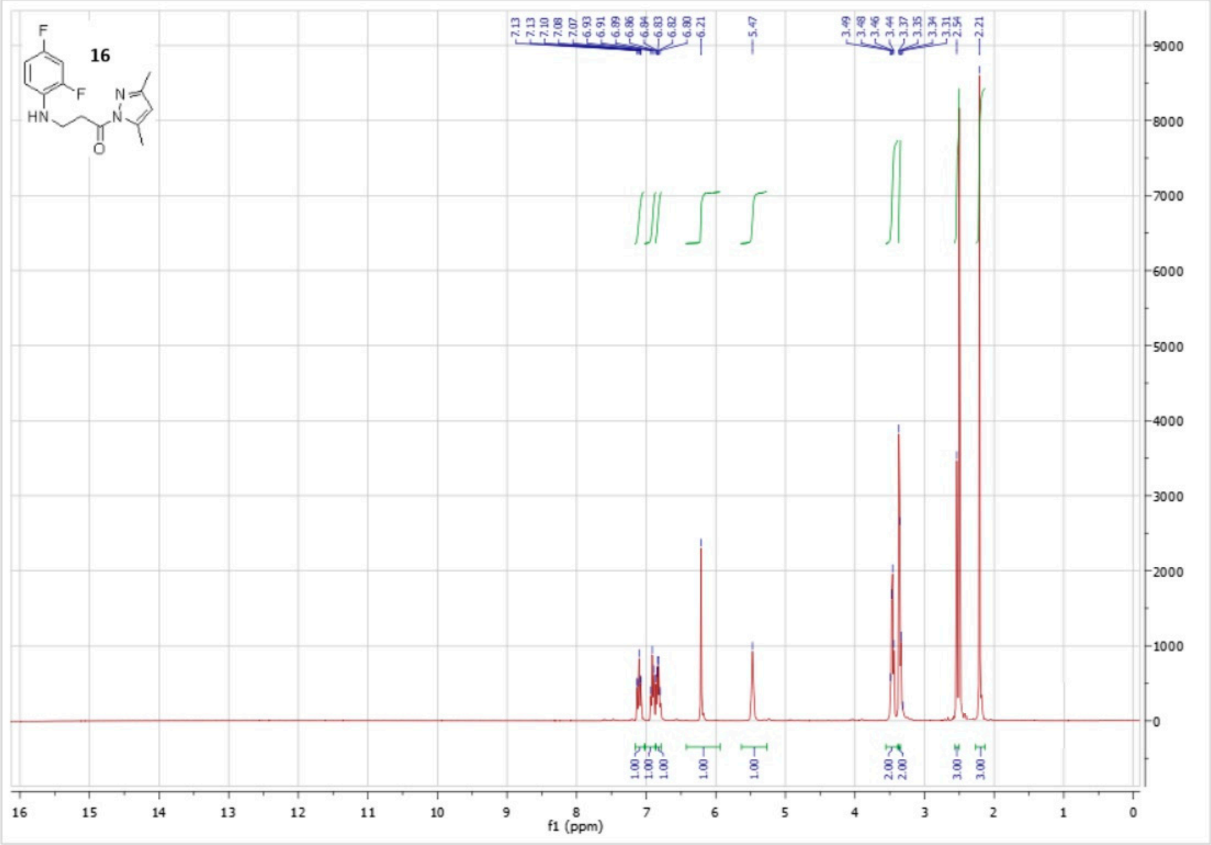

Figure S58. ^{13}C NMR spectrum of compound **16.**

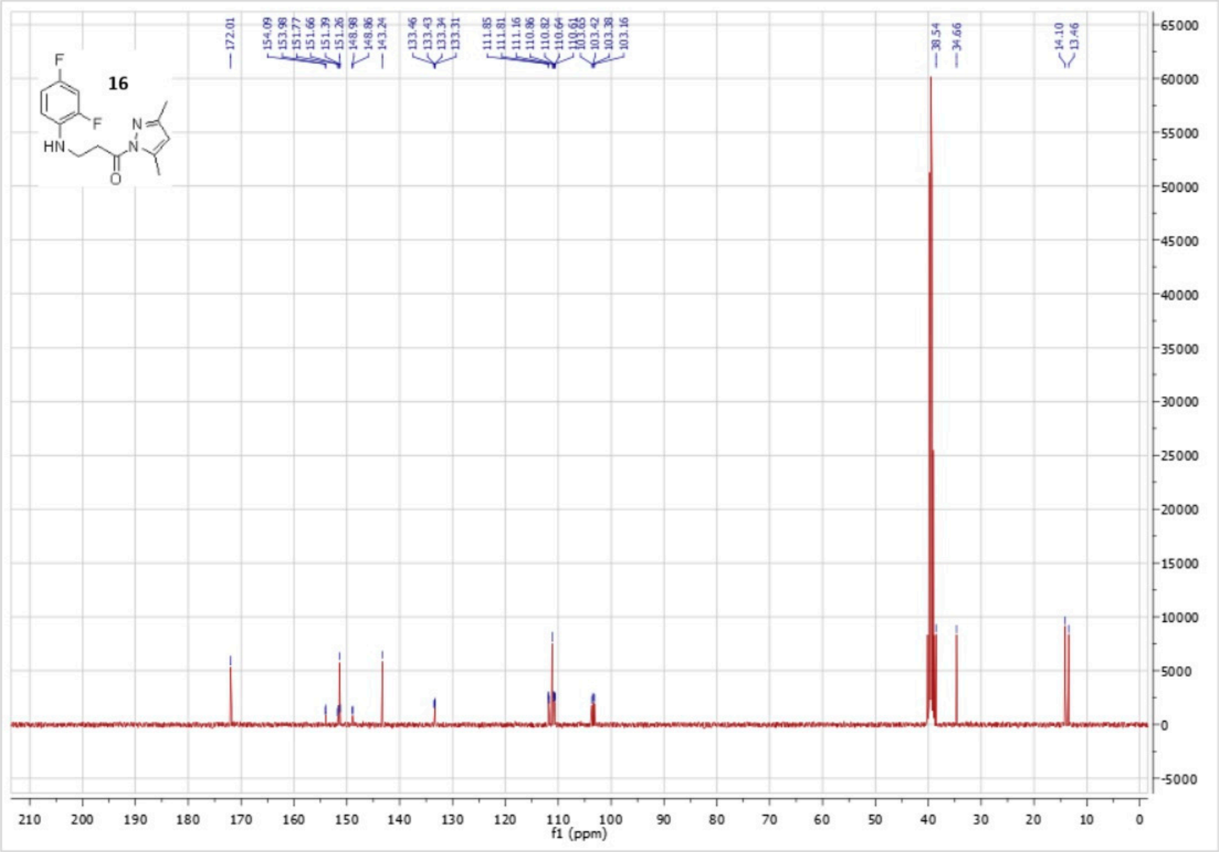

*3-[(2,4-Difluorophenyl)amino]-N-(2,5-dimethyl-1H-pyrrol-1-yl)propanamide (**17**)*

Figure S59. ^{1}H NMR spectrum of compound **17.**

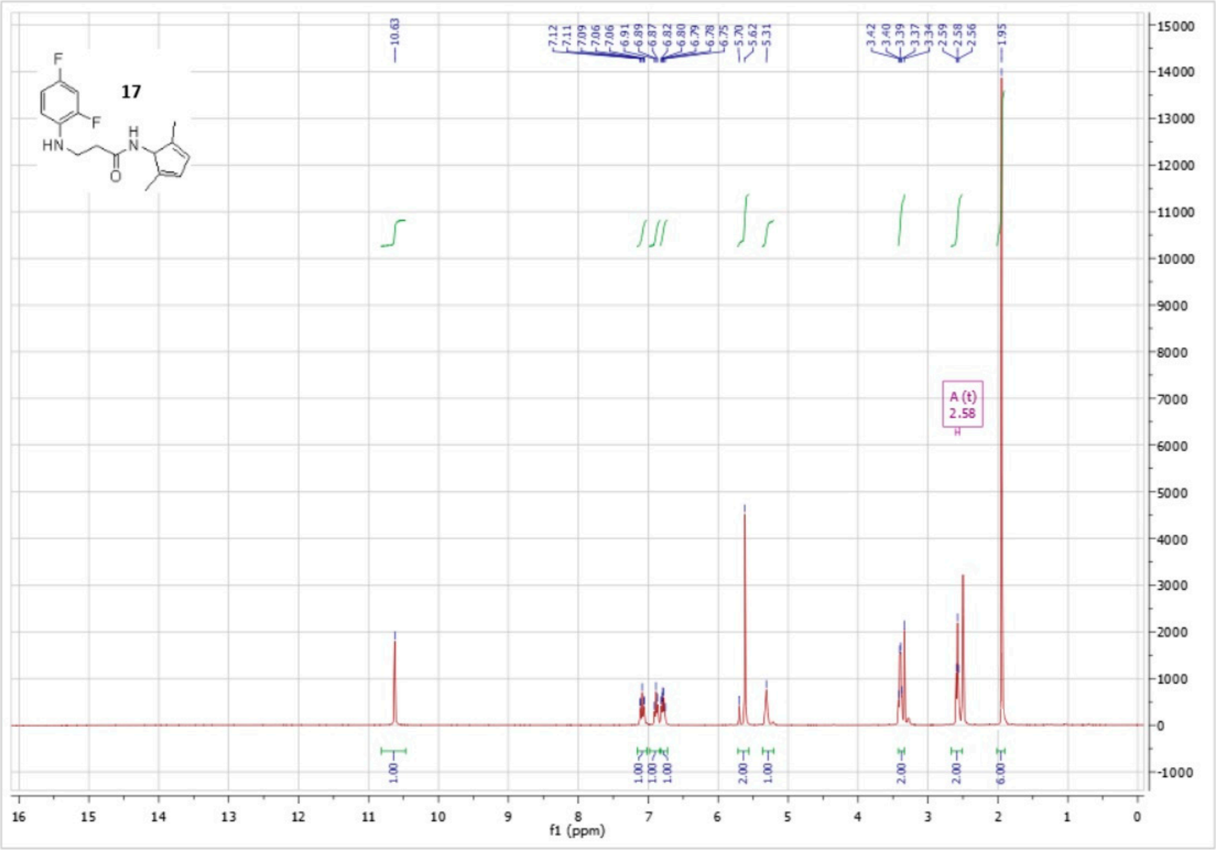

Figure S60. ^{13}C NMR spectrum of compound **17.**

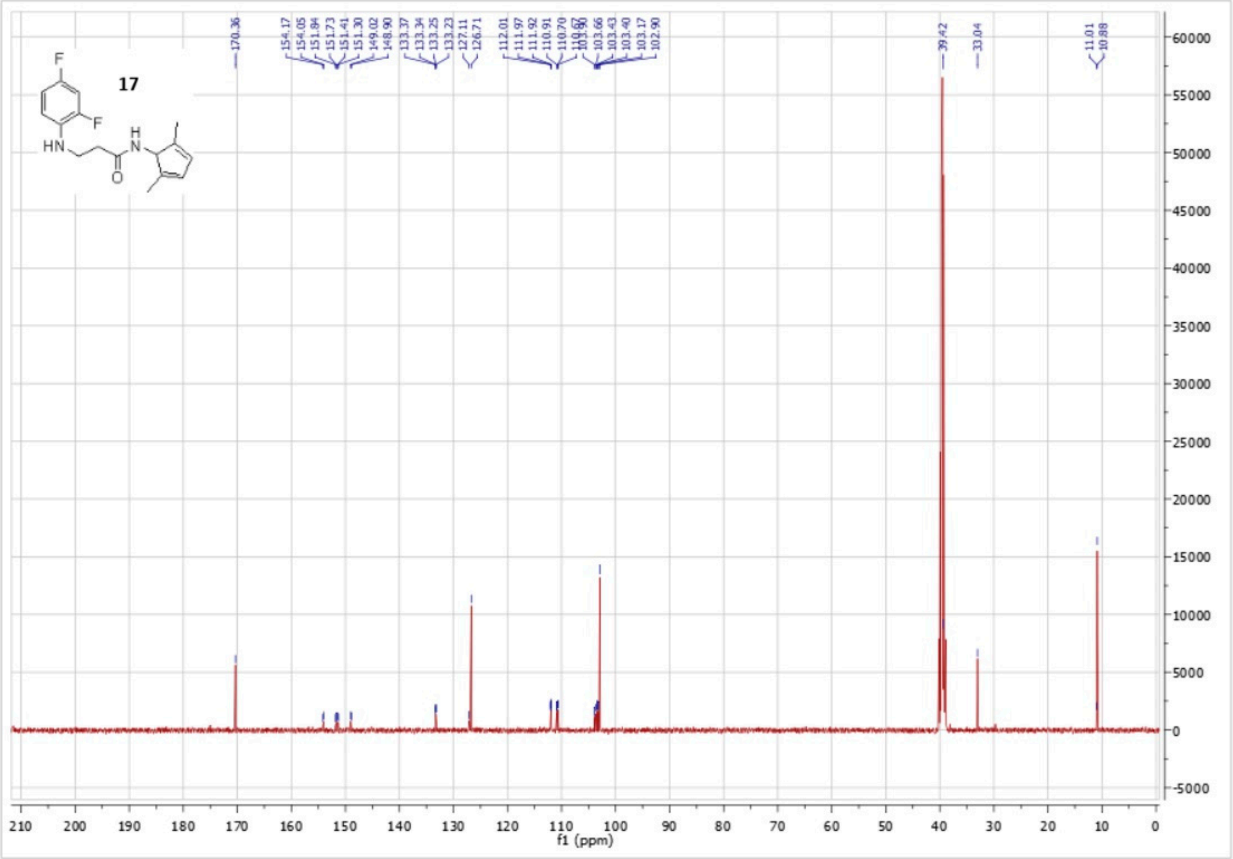

*2-{3-[(2,4-Difluorophenyl)amino]propanoyl}-N-phenylhydrazine-1-carboxamide (**18**)*

Figure S61. ^{1}H NMR spectrum of compound **18.**

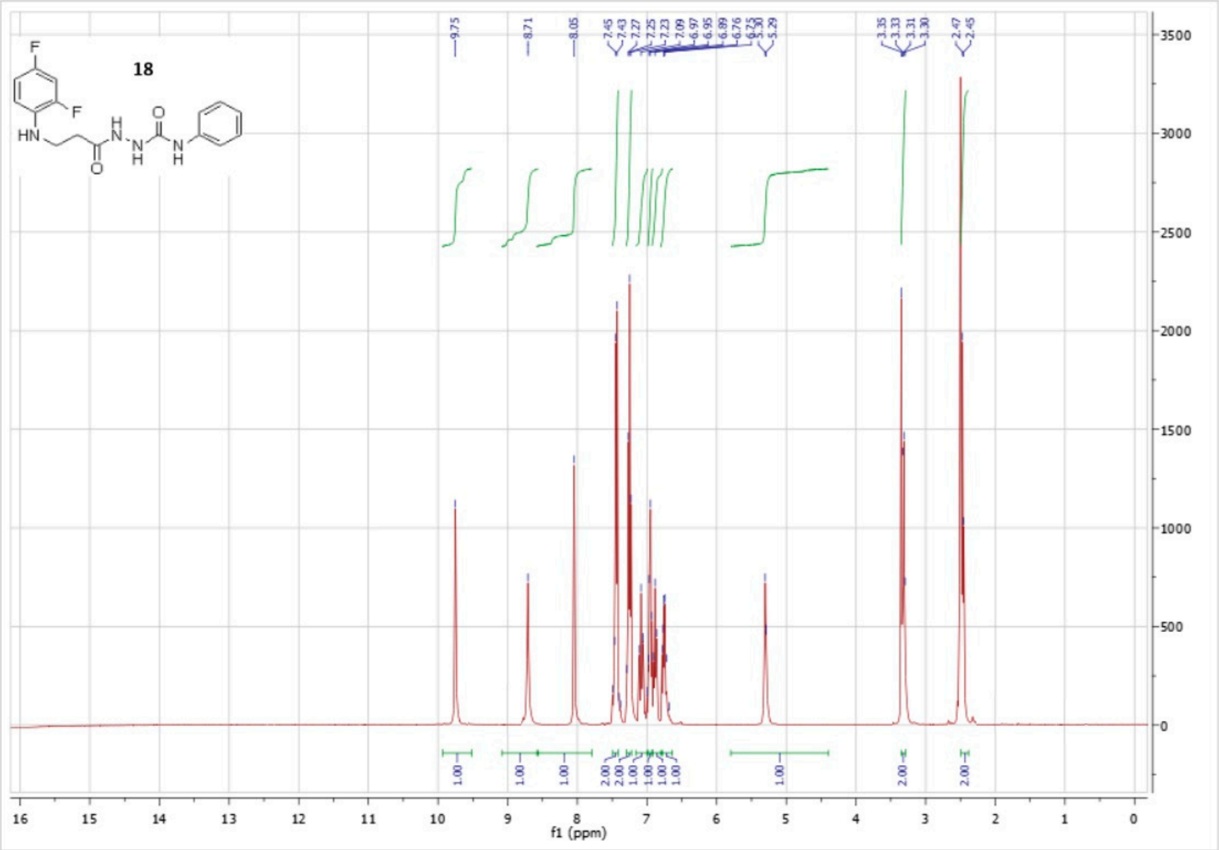

Figure S62. ^{13}C NMR spectrum of compound **18.**

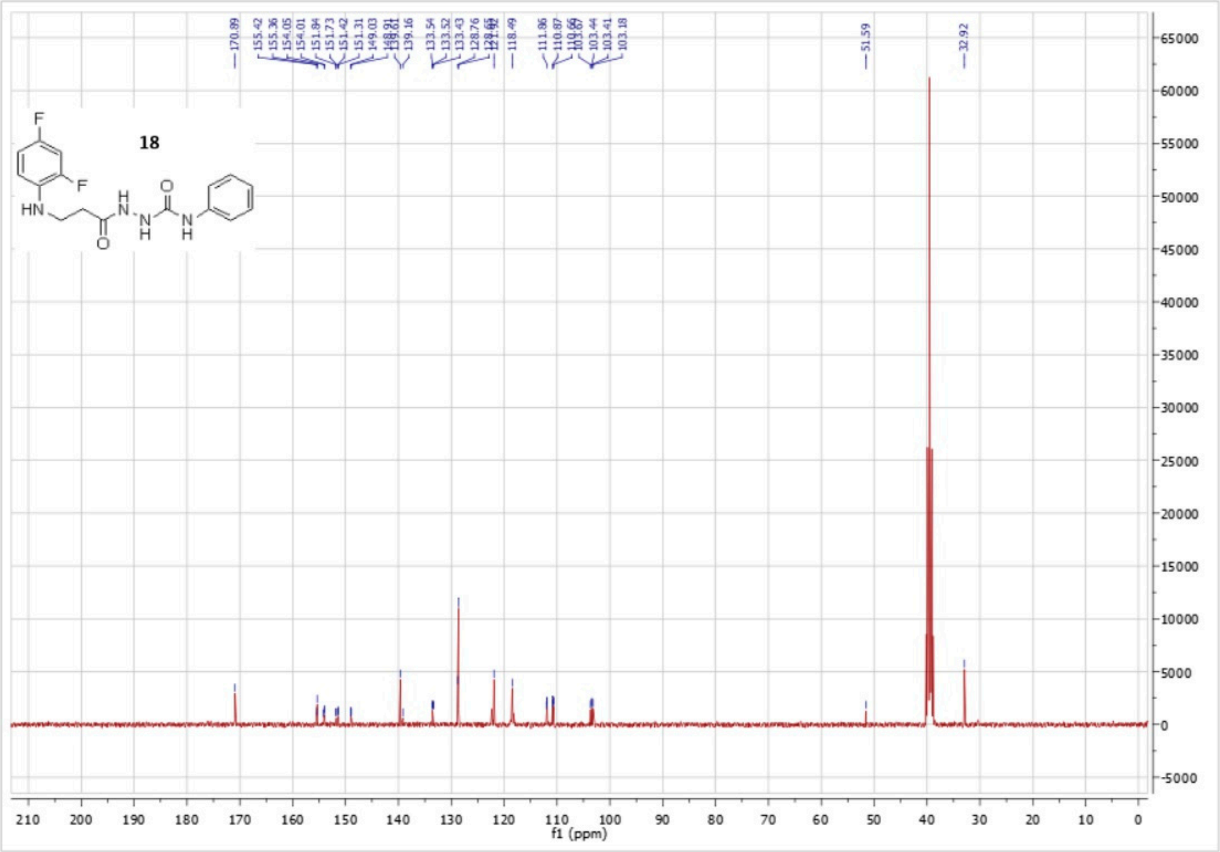

*2-{3-[(2,4-Difluorophenyl)amino]propanoyl}-N-phenylhydrazine-1-carbothioamide (**19**)*

Figure S63. ^{1}H NMR spectrum of compound **19.**

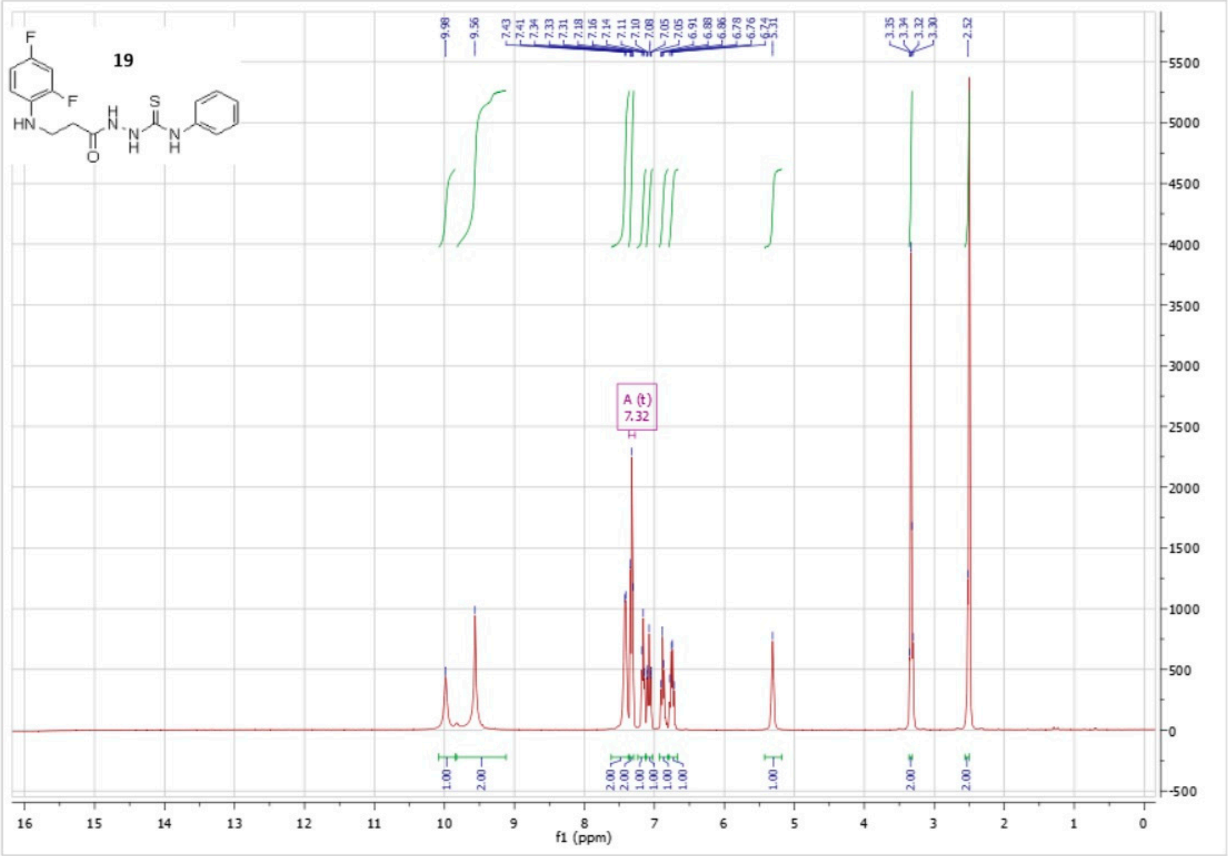

Figure S64. ^{13}C NMR spectrum of compound **19.**

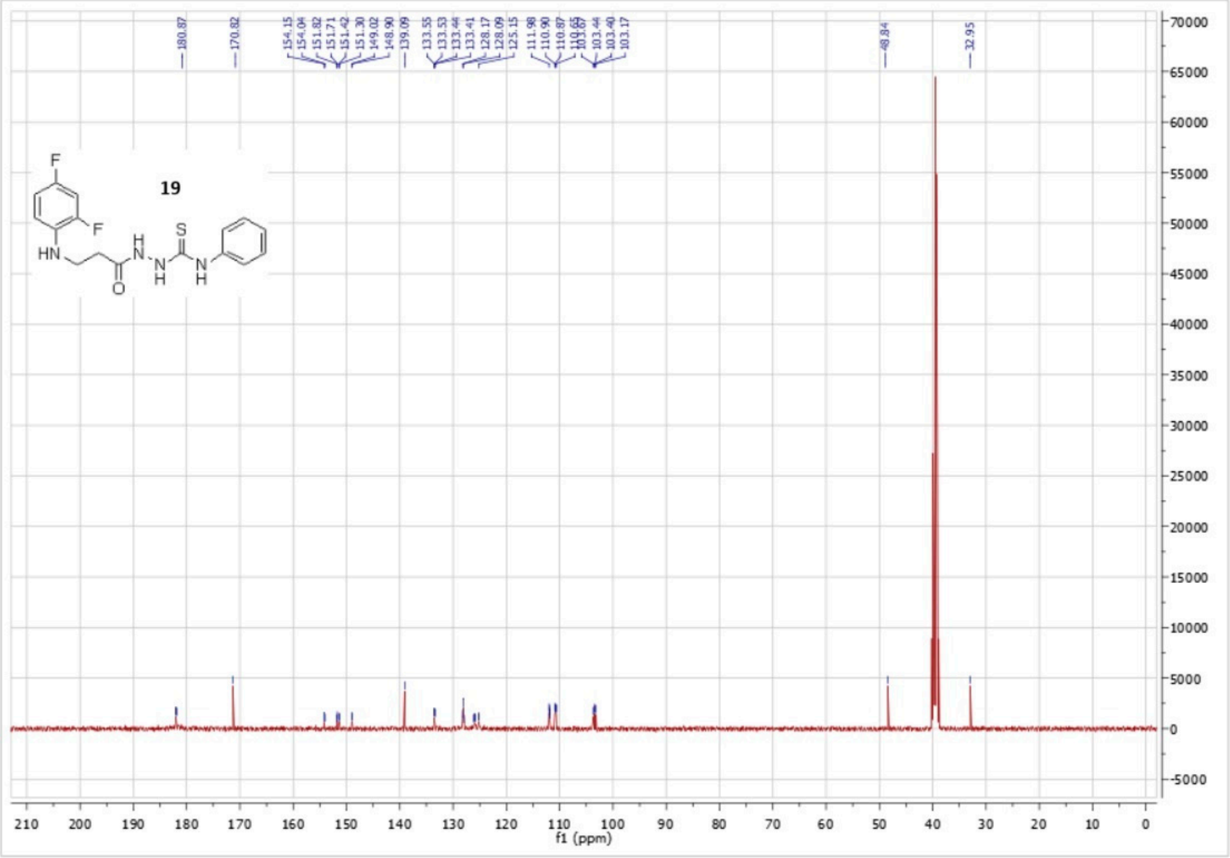

*5-{2-[(2,4-Difluorophenyl)amino]ethyl}-4-phenyl-2,4-dihydro-3H-1,2,4-triazol-3-one (**20**)*

Figure S65. ^{1}H NMR spectrum of compound **20.**

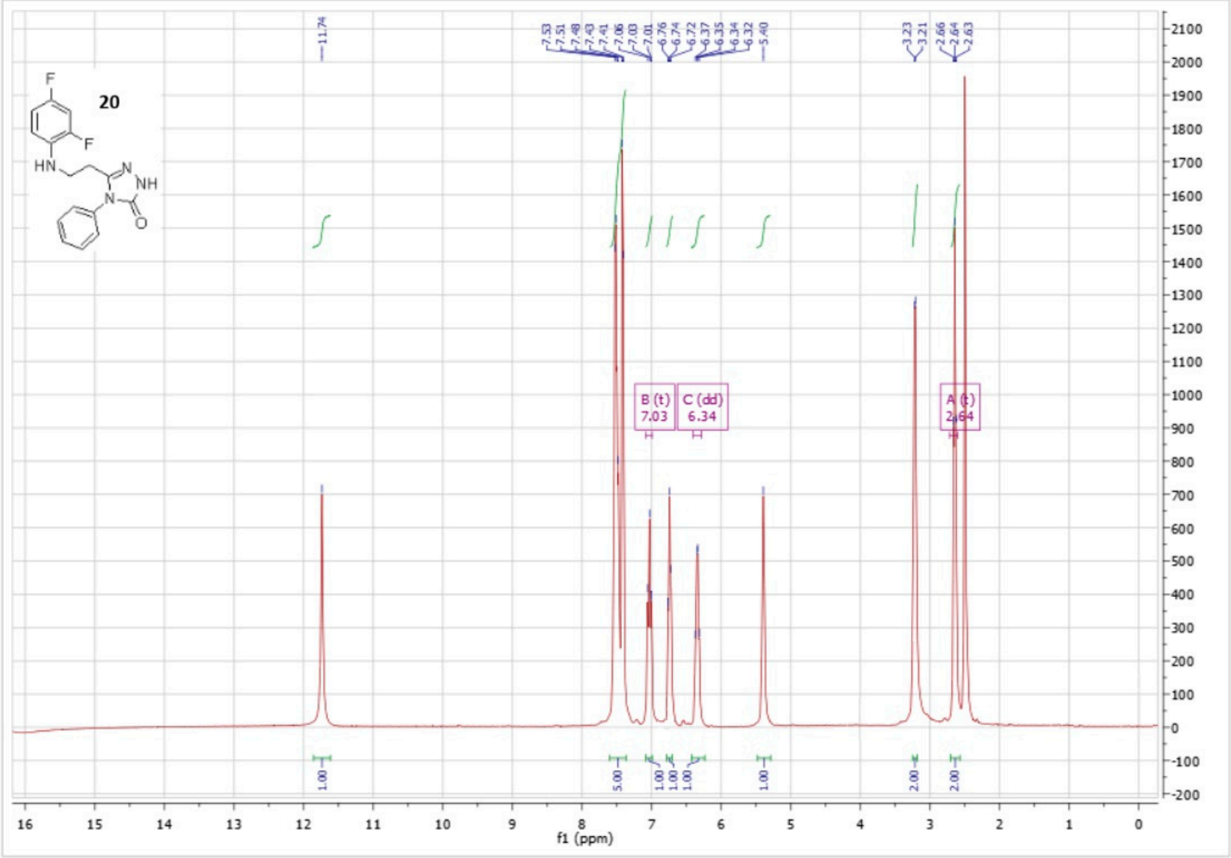

Figure S66. ^{13}C NMR spectrum of compound **20.**

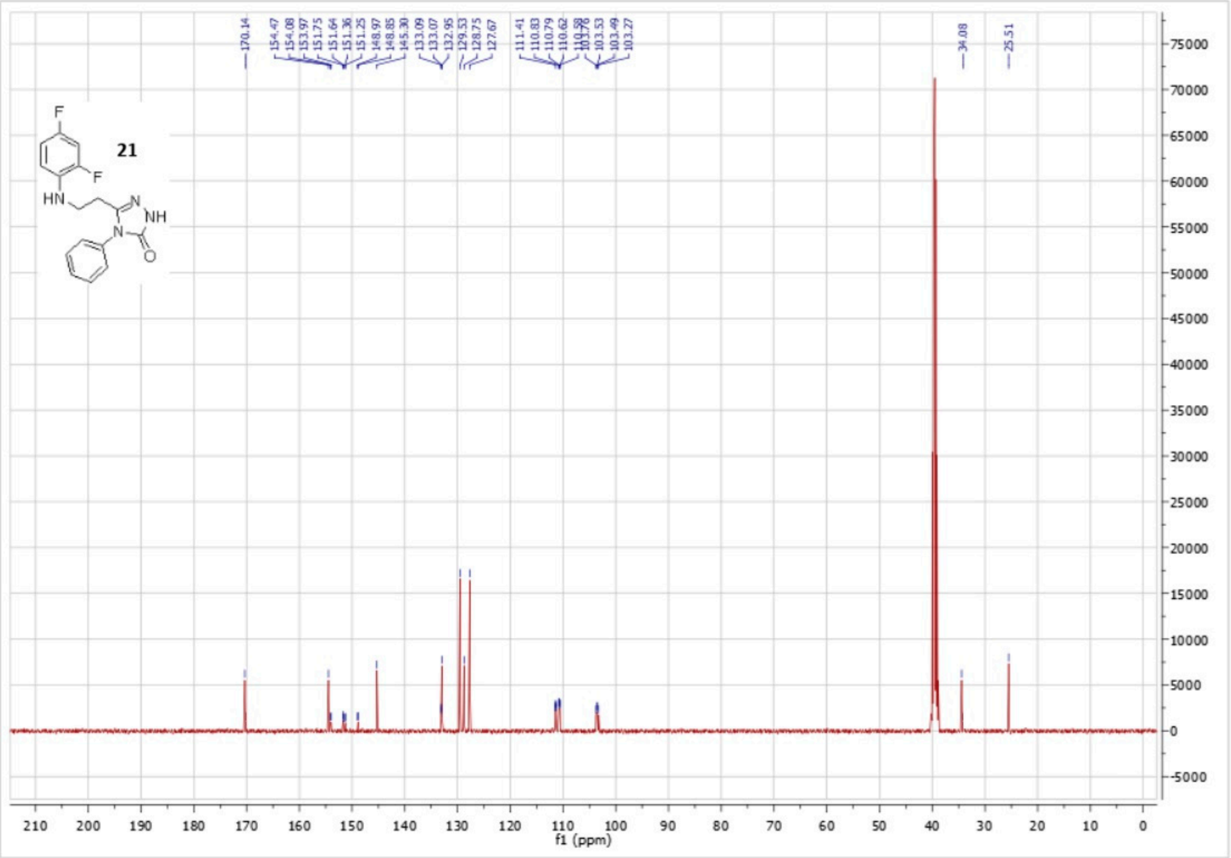

*5-{2-[(2,4-Difluorophenyl)amino]ethyl}-4-phenyl-2,4-dihydro-3H-1,2,4-triazole-3-thione (**21**)*

Figure S67. ^{1}H NMR spectrum of compound **21.**

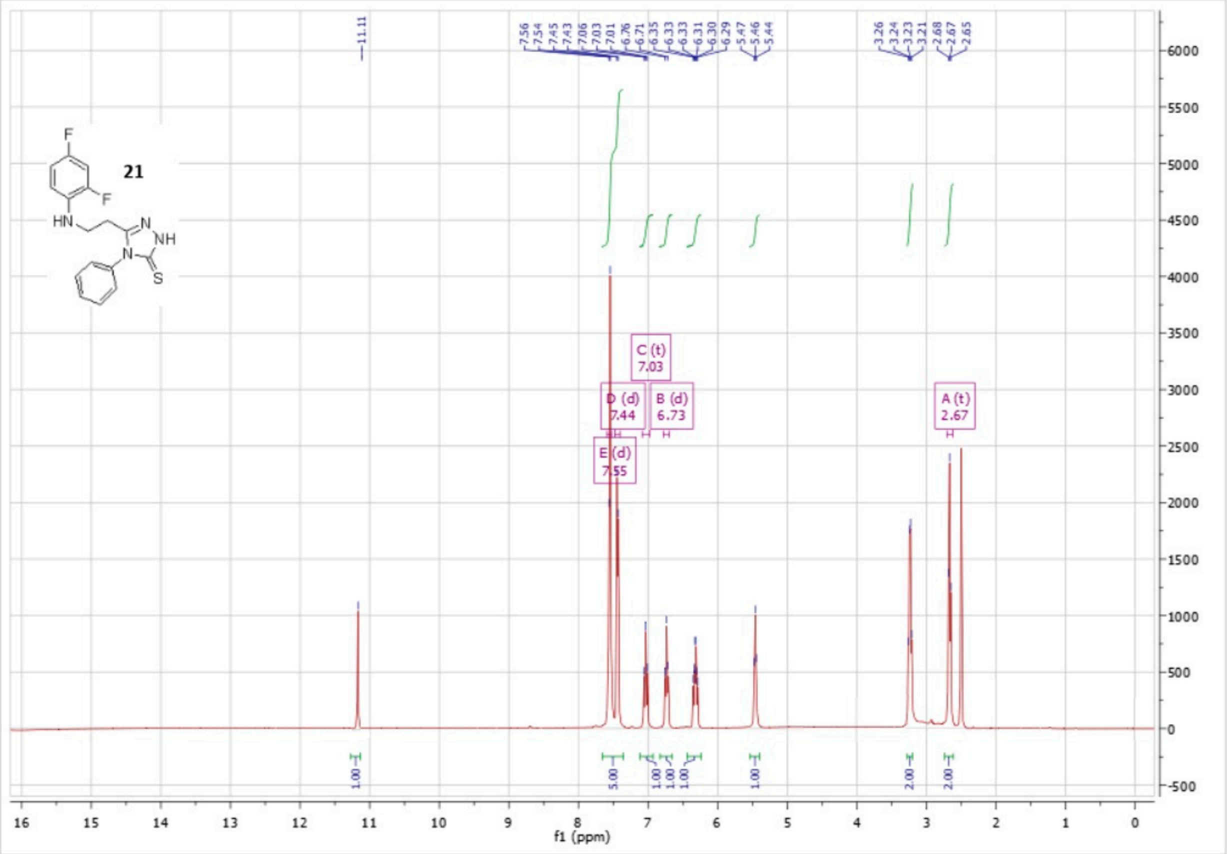

Figure S68. ^{13}C NMR spectrum of compound **21.**

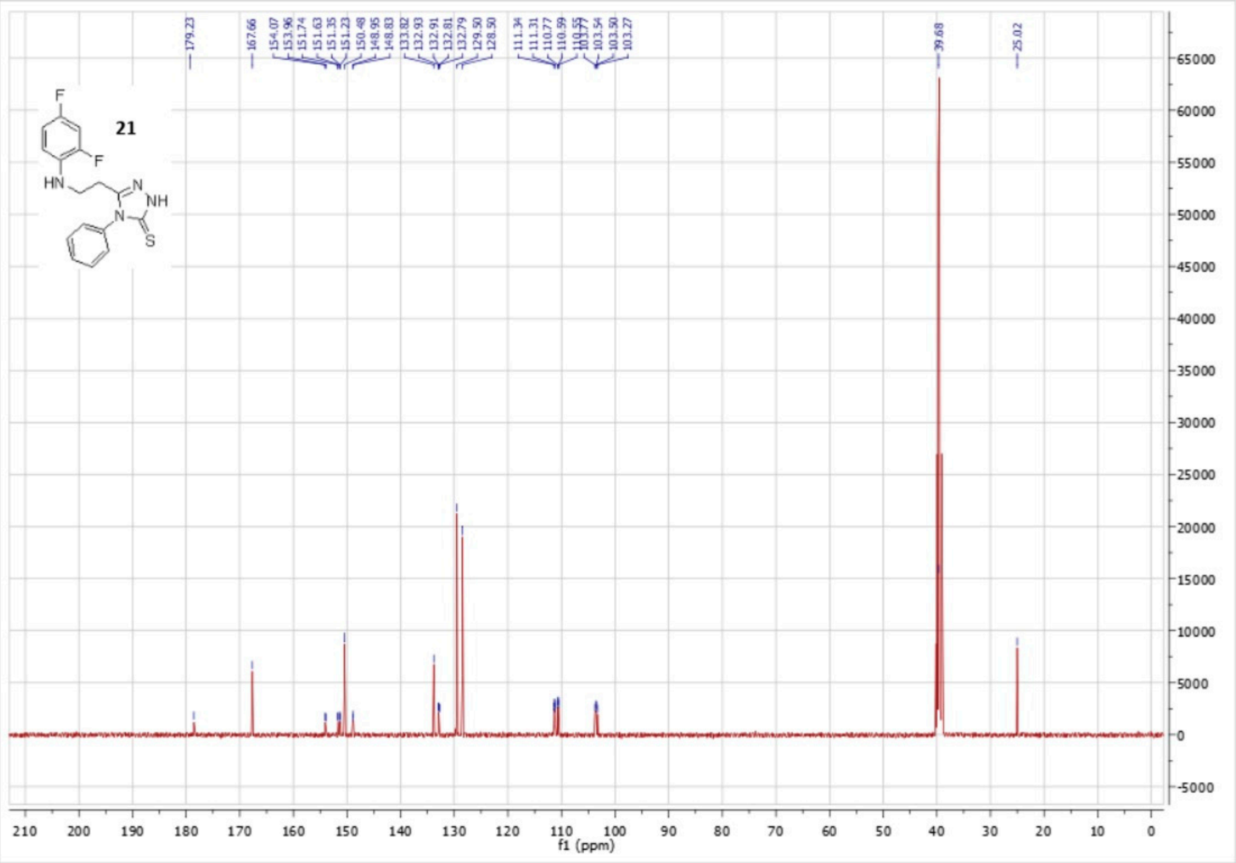

Table S1. Calculated IC_{50} values of target compounds **6b**, **7f**, **7g** and **9.**

**Compound**	**A549**	**Caco-2**
	**IC_{50} (µM)**	**IC_{50} (µM)**
**6b**	18,62	17,84
**7f**	13,47	13,06
**7g**	21,18	18,92
**9**	12,86	15,41
**CP**	4,93	4,67
**DOX**	1,94	2,21
